# Quassinoids from the Roots of *Eurycoma longifolia* and Their Anti-Proliferation Activities

**DOI:** 10.3390/molecules26195939

**Published:** 2021-09-30

**Authors:** Wei-Qun Yang, Wei Tang, Xiao-Jun Huang, Jian-Guo Song, Yue-Yue Li, Yu Xiong, Chun-Lin Fan, Zhen-Long Wu, Ying Wang, Wen-Cai Ye

**Affiliations:** 1 School of Pharmaceutical Sciences, Guangzhou University of Chinese Medicine, Guangzhou 510006, China; yangwq_zzy@gzucm.edu.cn; 2 Center for Bioactive Natural Molecules and Innovative Drugs Research, College of Pharmacy, Jinan University, Guangzhou 510632, China; tangv163@163.com (W.T.); zhyxiaohuang@163.com (X.-J.H.); songjianguof403@126.com (J.-G.S.); liyueyue7991@163.com (Y.-Y.L.); xyu0218@163.com (Y.X.); jnuchunlin@163.com (C.-L.F.); chywc@aliyun.com (W.-C.Y.); 3 Guangdong Province Key Laboratory of Pharmacodynamic Constituents of TCM and New Drugs Research, College of Pharmacy, Jinan University, Guangzhou 510632, China

**Keywords:** *Eurycoma longifolia*, Simaroubaceae, quassinoids, natural products, anti-proliferation activities

## Abstract

A phytochemical investigation on the roots of medicinal plant *Eurycoma longifolia* resulted in the isolation of 10 new highly oxygenated C_20_ quassinoids longifolactones G‒P (**1**–**10**), along with four known ones (**11**–**14**). Their chemical structures and absolute configurations were unambiguously elucidated on the basis of comprehensive spectroscopic analysis and X-ray crystallographic data. Notably, compound **1** is a rare pentacyclic C_20_ quassinoid featuring a densely functionalized 2,5-dioxatricyclo[5.2.2.0^4,8^]undecane core. Compound **4** represents the first example of quassinoids containing a 14,15-epoxy functionality, and **7** features an unusual α-oriented hydroxyl group at C-14. All isolated compounds were evaluated for their anti-proliferation activities on human leukemia cells. Among the isolates, compounds **5**, **12**, **13**, and **14** potently inhibited the in vitro proliferation of K562 and HL-60 cells with IC_50_ values ranging from 2.90 to 8.20 μM.

## 1. Introduction

Quassinoids are a class of highly oxygenated degraded triterpenoids mainly distributed in plant family Simaroubaceae [1]. Based on the number of carbon atoms involving the construction of their basic scaffolds, quassinoids are commonly categorized into six distinct groups: C_26_, C_25_, C_22_, C_20_, C_19_, and C_18_ types [2]. Quassinoids have been reported to display a wide range of biological activities, including antitumor, antimalarial, anti-inflammatory, antiviral, neuroprotective, and antifeedant activities [2,3]. Especially since the discovery of bruceantin, a C_20_ quassinoid isolated from *Brucea antidysenteria* (Simaroubaceae) in the early 1970s that showed remarkable antileukemic activity, the antitumor activities of quassinoids have attracted extensive attention from both chemical and biological communities [4,5,6,7].

*Eurycoma longifolia* Jack (Simaroubaceae), commonly known as “Tongkat Ali”, is a flowering shrub plant that widely distributed in Southeast Asia [8]. The roots of *E. longifolia* were traditionally used by local people for the treatment of malaria, dysentery, glandular swelling, persistent fever, aches, and sexual insufficiency [8]. Besides, the antitumor activities of the crude extract of *E. longifolia* roots were reported in 2005 [9]. Previous phytochemical investigations on the roots of *E. longifolia* have afforded a wide variety of chemical components, including quassinoids, canthin-6-one alkaloids, *β*-carboline alkaloids, tirucallane-type triterpenes, squalene derivatives, and biphenyl neolignans [10]. Among them, quassinoids are the most characteristic chemical constituents of this plant [11,12,13].

Previously, our group had reported the isolation and characterization of six novelquassinoids (longifolactones A‒F) with unprecedented C_26_ or C_20_ scaffolds from the petroleum ether-soluble fraction of the ethanol extract of *E. longifolia* roots [14]. Among them, longifolactione F is the first example of quassinoids containing an unprecedented densely functionalized 2,5-dioxatricyclo [5.2.2.0^4,8^]undecane ring system. In our continuing studies on searching structurally unique and biologically interesting metabolites from medicinal plants, the ethyl acetate-soluble fraction of the title plant was further investigated. As a result, longifolactones G‒P (**1**–**10**), 10 new C_20_ quassinoids, together with four known ones were isolated. Their structures and absolute configurations were unambiguously established by extensive spectroscopic data analysis and single-crystal X-ray diffraction experiment. Notably, compound **1** is the second member of the rare class of quassinoids featuring densely functionalized 2,5-dioxatricyclo[5.2.2.0^4,8^]undecane core. Besides, compound **4** represents the first example of quassinoids containing a 14,15-epoxy functionality, and compound **7** features an unusual 14α-OH substituent that makes **7** the second member of this rare class of quassinoids so far. Herein, we reported the isolation and structure elucidation of these new quassinoids. In addition, the in vitro anti-proliferation activities of all isolates on two human leukemia cell lines (K562 and HL-60 cells) were also described.

## 2. Results and Discussions

### 2.1. Quassinoids Isolated from E. longifolia

**Figure 1:**
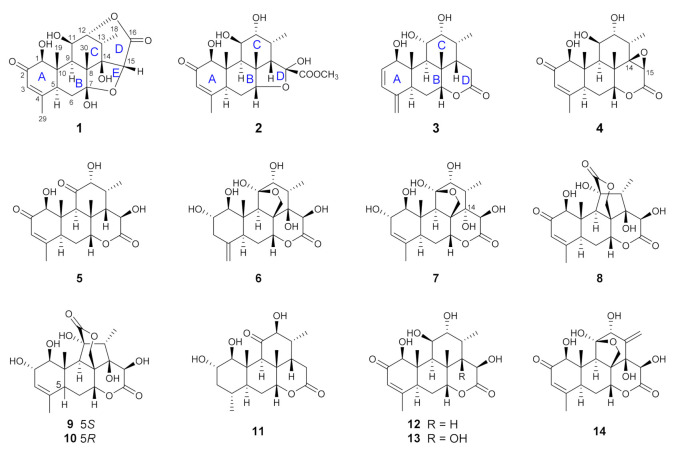
Chemical structures of quassinoids isolated from the roots of *E. longifolia*.

The air-dried and powdered roots of *E. longifolia* (10 kg) were extracted with 95% ethanol under room temperature for five times. The ethanol extract (270 g) was suspended in water and partitioned successively with petroleum ether, ethyl acetate, and *n*-butanol. The ethyl acetate-soluble fraction was investigated in present study. By performing a series of charomatographic procedures on the aforementioned fraction, 10 new C_20_ quassinoids (longifolactones G‒P, **1**‒**10**), along with four known C_20_ quassinoids, chaparrolide (**11**) [15], 15*β*-hydroxyklaineanone (**12**) [16], 14,15*β*-dihydroxyklaineanone (**13**) [17], and eurycomanone (**14**) [18], were isolated (Figure 1).

### 2.2. Structure Elucidation of the New Quassinoids

Compound **1** was obtained as colorless needles. Its molecular formula was determined as C_2__0_H_2__6_O_8_ by the HR-ESI-MS ion peak at *m/z* 395.1696 [M + H]^+^ (calcd for C_20_H_27_O_8_, 395.1700) and ^13^C NMR data. The UV spectrum of **1** displayed absorption maxima at 241 nm. Its IR spectrum revealed the characteristic absorptions for hydroxyl (3455 cm^−1^) and carbonyl (1730 and 1667 cm^−1^) functional groups. In the ^1^H and ^13^C NMR spectra of **1**, signals corresponding to two hydroxyl groups [*δ*_H_ 7.05 (1H, s) and 6.89 (1H, d, *J* = 5.7 Hz)], a ketone carbonyl (*δ*_C_ 199.7), a ester carbonyl (*δ*_C_ 171.5), a trisubstituted double bond [*δ*_H_ 6.07 (1H, br s); *δ*_C_ 163.3 and 125.6], a hemiketal carbon (*δ*_C_ 111.0), an oxygenated quaternary carbon (*δ*_C_ 82.9), four oxygenated methines [*δ*_H_ 5.45 (1H, m), 5.13 (1H, s), 4.72 (1H, dd, *J* = 4.6, 1.8 Hz), and 4.00 (1H, s); *δ*_C_ 85.5, 83.5, 82.7, and 69.5], three methines, a methylene, two quaternary carbons, and four methyl groups [*δ*_H_ 1.96 (3H, s), 1.70 (3H, s), 1.39 (3H, s), and 1.34 (3H, d, *J* = 7.2 Hz); *δ*_C_ 21.6, 13.0, 12.9, and 12.8] were observed, indicative of a C_20_ quassinoid skeleton for **1**. With the aid of 2D NMR spectroscopic data, all proton and carbon resonances of **1** were assigned (Tables S1 and S2).

**Figure 2:**
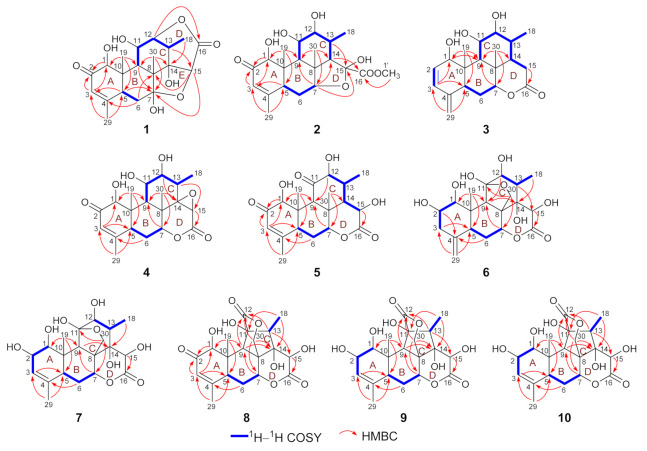
Key ^1^H–^1^H COSY and HMBC correlations of compounds **1**–**10**.

The above NMR spectroscopic data of **1** were closely similar to those of longifolactone F [14], suggesting the structural similarity of these two compounds. Different from longifolactone F, the NMR signals corresponding to a methylene group (CH_2_-3) and a methine group (CH-4) were replaced by resonances of a double bond [*δ*_H_ 6.07 (1H, br s); *δ*_C_ 163.3 and 125.6] in **1**. In the HMBC spectrum, key correlations between H_3_-29 and C-3, H-5 and C-3, H_2_-6 and C-4 were observed, suggesting the presence of an *α*,*β*-unsaturated ketone motif in ring A of **1**, which was also confirmed by the characteristic chemical shift values of C-2‒C-4 (*δ*_C_ 199.7, 125.6, and 163.3). After a comprehensive interpretation of its ^1^H–^1^H COSY and HMBC spectra, the gross structure of **1** was established as a C_20_ quassinoid with a rare 2,5-dioxatricyclo[5.2.2.0^4,8^]undecane core (Figure 2).

**Figure 3:**
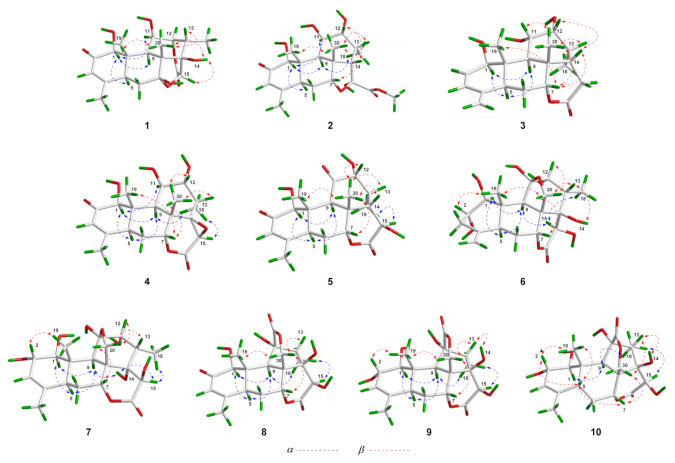
Key NOESY correlations of compounds **1**–**10**.

**Figure 4:**
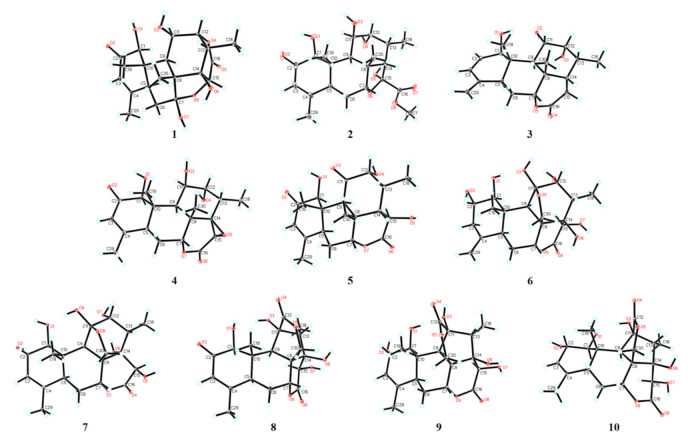
X-ray ORTEP drawings of compounds **1**–**10**.

In the NOESY spectrum of **1**, key NOE correlations between H-1 and H-5/H-11, H-5 and H-9 were observed, indicating that these protons had the same orientation. Meanwhile, the NOE correlations between OH-14 and H-15/H_3_-30, H-13 and H_3_-30, H_3_-19 and H_3_-30 were observed, suggesting that these protons were located on the same face of the molecule (Figure 3). Finally, suitable crystals of **1** were acquired. The following X-ray diffraction analysis with Cu Kα radiation resulted in an excellent Flack parameter of 0.06 (4), which not only allowed the verification of the planar structure of **1** but also led to the establishment of its absolute configuration as 1*S*,5*S*,7*R*,8*R*,9*R*,10*S*,11*R*,12*R*,13*S*,14*R*,15*R* (Figure 4).

The molecular formula of **2** was deduced as C_21_H_30_O_8_ on the basis of its HR-ESI-MS data (*m/z* 433.1832 [M + Na]^+^; calcd for C_21_H_30_O_8_Na, 433.1833), and the ^13^C NMR data analysis. The ^1^H and ^13^C NMR spectroscopic data of **2** (Tables S1 and S2) highly resembled those of 6-dehydroxylongilactone [19], except for the presence of additional signals due to a hemiketal carbon (*δ*_C_ 102.5) and an oxygenated methyl group [*δ*_H_ 3.83 (3H, s); *δ*_C_ 52.7] in **2**. Subsequently, detailed analysis of its ^1^H–^1^H COSY and HMBC spectra allowed the establishment of a 6/6/6/5 ring system for **2** that was identical to 6-dehydroxylongilactone. Besides, the HMBC correlation between 1′-OCH_3_ and C-16 indicated the presence of an extra methoxycarbonyl group in **2**. Based on the molecular formula information, as well as the obvious down-field shift of C-15 (*δ*_C_ 102.5), the remaining methoxycarbonyl and hydroxyl groups were both assigned to attach to C-15, which was also confirmed by the HMBC correlation between H-14 and C-16 (Figure 2). Thus, the planar structure of **2** was established. Finally, the structure of **2** was fully resolved by an X-ray diffraction experiment. With an excellent Flack parameter of 0.09 (8), the absolute configuration of **2** was assigned as 1*S*,5*S*,7*R*,8*R*,9*R*,10*S*,11*R*,12*R*,13*R*,14*S*,15*S* (Figure 4).

The molecular formula of **3** was determined to be C_20_H_28_O_5_ on the basis of its sodiated molecular ion peak at *m/z* 371.1831 [M + Na]^+^ (calcd for C_20_H_28_O_5_Na, 371.1829) and ^13^C NMR data. The ^1^H and ^13^C NMR spectral data of **3** were similar to those of the co-isolated known compound chaparrolide (**11**), which indicated that **3** was also a C_20_ quassinoid. Compared with those of **11**, the ^1^H and ^13^C NMR spectra of **3** showed additional signals for a *cis*-disubstituted double bond [*δ*_H_ 6.31 (1H, d, *J* = 9.6 Hz) and 5.76 (1H, d, *J* = 9.6 Hz); *δ*_C_ 132.7 and 130.2] and an *exo*-olefin group [*δ*_H_ 5.00 (1H, s) and 4.84 (1H, s); *δ*_C_ 145.8 and 111.3], while the signals corresponding to a ketone carbonyl, a methylene group, a methine group, and a methyl group were absent. In the ^1^H–^1^H COSY spectrum of **3**, the correlation between H-11 and H-12 indicated that the C-11 in **3** was an oxygenated-substituted methine instead of the ketone carbonyl in **11** (Figure 2). Moreover, the observed HMBC cross-peaks between H_2_-29 and C-3/C-5, H-3 and C-5, H-3 and C-1 indicated the presence of two conjugated double bonds in ring A of **3** (Figure 2). Similarly, an X-ray diffraction experiment using Cu K*α* radiation was performed, which led to the full assignment of planar structure and absolute configuration for **3** (1*R*,5*S*,7*R*,8*S*,9*R*,10*S*,11*S*,12*R*,13*R*,14*S*, Figure 4).

The molecular formula of **4** was assigned as C_20_H_26_O_7_ based on its HR-ESI-MS data (*m/z* 401.1574 [M + Na]^+^; calcd for C_20_H_26_O_7_Na, 401.1571) and ^13^C NMR spectroscopic data, 18 mass units less than the co-isolated known C_20_ quassinoid, 14,15*β*-dihydroxyklaineanone (**13**). The NMR spectra of **4** showed characteristic signals similar to those of **13**, except for the presence of one oxygen-bearing methine [*δ*_H_ 3.36 (1H, s); *δ*_C_ 52.9] and one oxygenated bearing quaternary carbon (*δ*_C_ 67.9). Considering the molecular formula information, the two hydroxyl groups at C-14 and C-15 in **13** were replaced by an epoxide ring in **4**. Furthermore, the NOE correlation between H-15 and H_3_-18 in the NOESY spectrum suggested that the epoxide ring had the *β*-orientation (Figure 3). Similar to **1**–**3**, the structure with absolute configuration (1*S*,5*S*,7*R*,8*S*,9*R*,10*S*,11*R*,12*R*,13*S*,14*R*,15*R*) of **4** was definitively assigned by an X-ray diffraction experiment (Figure 4).

The molecular formula of **5** was determined as C_20_H_26_O_7_ by the HR-ESI-MS ion peak at *m/z* 401.1574 [M + Na]^+^ (calcd for C_20_H_26_O_7_Na, 401.1571) and ^13^C NMR data. The ^1^H and ^13^C NMR spectral data of **5** (Tables S1 and S2) were very similar to those of 11-dehydroklaineanone [20]. The main differences were that the signals corresponding to a methylene group [*δ*_H_ 3.69 (1H, dd, *J* = 19.4, 12.7 Hz) and 2.70 (1H, dd, *J* = 19.4, 6.6 Hz); *δ*_C_ 29.1] in the known compound were replaced by the signals due to an oxygenated methine [*δ*_H_ 5.42 (1H, d, *J* = 10.1 Hz); *δ*_C_ 67.3] in **5**, suggesting the presence of an additional hydroxyl group at C-15 in **5**. This assumption was further confirmed by the HMBC cross-peak between H-15 and C-16 (Figure 2). Subsequently, the planar structure and absolute configuration (1*S*,5*S*,7*R*,8*S*,9*R*,10*S*,12*R*,13*R*,14*S*,15*R*) of **5** were completely deduced by a single-crystal X-ray diffraction experiment (Figure 4).

The HR-ESI-MS of **6** displayed a sodiated molecular ion peak at *m/z* 435.1626 [M + Na]^+^, corresponding to a molecular formula of C_20_H_28_O_9_. Comparison of the ^1^H and ^13^C NMR spectral data of **6** (Tables S1 and S3) with those of Δ^4,5^,14-hydroxyglaucarubol [21] revealed that they were closely similar, except for signals for the *endo*-olefin (*δ*_C_ 130.1 and 127.5) and a methyl [*δ*_H_ 1.75 (3H, s); *δ*_C_ 20.2] in Δ^4,5^,14-hydroxyglaucarubol were replaced by signals of an *exo*-olefin [*δ*_H_ 4.98 (1H, s) and 4.74 (1H, s); *δ*_C_ 147.4 and 110.1] and a methine [*δ*_H_ 2.74 (1H, overlapped); *δ*_C_ 42.5] in **6**. In the HMBC spectrum, correlations between H_2_-29 and C-3/C-5 indicated that the *exo*-olefin was located at C-4 (29) (Figure 2). Thus, the planar structure of **6** was established. Similarly, the relative stereostructure and absolute configuration (1*S*,2*S*,5*S*,7*R*,8*R*,9*R*,10*S*,11*R*,12*R*,13*S*,14*R*,15*R*) of **6** were established by a single-crystal X-ray diffraction experiment (Figure 4).

The molecular formula of **7** was deduced as C_20_H_28_O_9_ by its HR-ESI-MS data (*m/z* 435.1621 [M + Na]^+^; calcd for C_20_H_28_O_9_Na, 435.1626) and ^13^C NMR data. The NMR spectroscopic features of **7** were similar to those of 14-*epi*-13,21-dihydroeurycomanone [22], except for the presence of signals assigned to an oxygenated methine [*δ*_H_ 4.63 (1H, br s); *δ*_C_ 72.3] in 7, while the signal corresponding to a ketone carbonyl carbon was absent. The above data suggested that **7** was the C-2 hydroxylated derivative of 14-*epi*-13,21-dihydroeurycomanone. This assumption was confirmed by the spin system deduced from H-1 to H-3 in the ^1^H–^1^H COSY spectrum of **7** (Figure 2). Furthermore, the NOE correlation between H-2 and H_3_-19 in the NOESY spectrum suggested that the H-2 and H_3_-19 had the same orientation (Figure 3). Finally, with a Flack parameter of −0.12 (9), the absolute structure of **7** (1*S*,2*S*,5*S*,7*R*,8*R*,9*R*,10*S*,11*R*,12*R*,13*S*,14*S*,15*R*) was unambiguously established (Figure 4).

The HR-ESI-MS of compound **8** displayed a sodiated molecular ion peak at *m/z* 431.1311 [M + Na]^+^ (calcd for C_20_H_24_O_9_Na, 431.1313), allowing the determination of a molecular formula of C_20_H_2__4_O_9_ that was identical to the known C_20_ quassinoid 13-*epi*-eurycomadilactone [21]. The ^1^H and ^13^C NMR spectral data of **8** (Tables S1 and S3) closely resembled those of 13-*epi*-eurycomadilactone, combined with its molecular formula information, suggesting that **8** was a stereoisomer of the known compound. Further analysis of the 2D NMR data of **8** confirmed that 8 had the same planar structure as 13-*epi*-eurycomadilactone. Different from 13-*epi*-eurycomadilactone, the NOESY spectrum of **8** showed the correlation between H-13 and H_2_-30, indicating the α-orientation for the H_3_-18 in **8** (Figure 3). The structure with absolute configuration (1*S*,5*S*,7*R*,8*R*,9*R*,10*S*,11*S*,13*R*,14*R*,15*R*) of **8** was finally determined on the basis of an X-ray crystallography study by using the anomalous dispersion of Cu Kα radiation (Figure 4).

Compound **9** was assigned to possess a molecular formula of C_20_H_26_O_9_ by the HR-ESI-MS ion peak at *m/z* 433.1472 [M + Na]^+^ (calcd for C_20_H_26_O_9_Na, 433.1469) and 1D NMR spectral data analysis, which was two mass units more than that of **8**. The ^1^H and ^13^C NMR spectra of **9** exhibited similar signals to those of **8** (Tables S1 and S3), except for the signal assigned to a ketone carbonyl (*δ*_C_ 197.0, C-2 in **8**) was replaced by the signals of an oxygenated methine [*δ*_H_ 4.55 (1H, overlapped); *δ*_C_ 72.5] in **9**. Thus, compound **9** was assumed to be a C-2 hydroxylated derivative of **8**. This deduction was further verified by the spin system from H-1 to H-3 in the ^1^H–^1^H COSY spectrum of **9** (Figure 2). Furthermore, the α-orientation of the 2-OH was determined on the basis of key NOE correlation between H-2 and H_3_-19 (Figure 3). A further crystallographic analysis led to the unambiguous establishment of the structure and absolute configuration (1*S*,2*S*,5*S*,7*R*,8*R*,9*R*,10*S*,11*S*,13*R*,14*R*,15*R*) of **9** (Figure 4).

The molecular formula of **10** was deduced to be identical to that of **9** on the basis of its HR-ESI-MS data (*m/z* 433.1470 [M + Na]^+^; calcd for C_20_H_26_O_9_Na, 433.1469) and ^13^C NMR data. Comparison of the NMR data of **10** with those of **9** (Tables S1 and S3) indicated that **10** possessed the identical gross structure to **9**. The main differences of the NMR spectral data between **10** and **9** were the obvious down-field shifts of C-5 (Δ*δ* +5.1) and C-6 (Δ*δ* +5.2) in **10**, suggesting that **10** might be a C-5 epimer of **9**. Further analysis of its 2D NMR spectroscopic data verified that **10** possessed the identical planar structure to **9**. In the NOESY spectrum, NOE correlation between H-5 and H_3_-19 was observed, suggesting the *β*-orientation for H-5 in **10** (Figure 3). Similar to **1**–**9**, the single-crystal X-ray diffraction study (Cu Kα) allowed the assignment of the complete stereochemistry of **10**. As a result, the absolute configuration of **10** was definitively assigned to be 1*S*,2*S*,5*R*,7*R*,8*R*,9*R*,10*S*,11*S*,13*R*,14*R*,15*R* (Figure 4).

### 2.3. Anti-proliferation Activities of Isolated Quassinoids

The isolated compounds were tested for their anti-proliferation activities on two human leukemia cell lines, K562 and HL-60. As shown in Table S5, compounds **5**, **12**, **13**, and **14** exhibited potent inhibitory effects on the proliferation of both K562 and HL-60 cells with IC_50_ values ranging from 2.90 to 8.20 μM.

## 3. Materials and Methods

### 3.1. General Methods

Melting points were measured on an X-5 melting point instrument (Fukai, Beijing, China) without correction. Optical rotations were determined in MeOH on a P-1020 polarimeter (JASCO, Tokyo, Japan) with a 1 cm cell at room temperature. UV spectra were acquired on a JASCO V-500 UV/vis spectrometer. IR spectra were obtained with a JASCO FT/IR-480 plus infrared spectrometer using KBr pellets. HR-ESI-MS data were collected using an Agilent 6210 TOF-MS spectrometer (Agilent Technologies, Santa Clara, CA, USA). Other experimental procedures were performed as described previously [14]. The human leukemia cell lines, HL-60 and K562, were purchased from the American Type Culture Collection (ATCC) and cultured in RPMI-1640 medium supplemented with 10% fetal bovine serum (FBS) and 2 mM L-glutamine.

### 3.2. Plant Material

The roots of *Eurycoma longifolia* were collected from Malacca, Malaysia, in June 2014 and authenticated by Prof. Guang-Xiong Zhou (College of Pharmacy, Jinan University). A voucher specimen (No. 20140501) was deposited in the Center for Bioactive Natural Molecules and Innovative Drugs Research, College of Pharmacy, Jinan University.

### 3.3. Extraction and Isolation

The air-dried and powdered roots of *E. longifolia* (10 kg) were extracted with 95% (*v/v*) EtOH five times at room temperature. The combined EtOH extract was concentrated under vacuum to yield a crude extract (270 g), which was suspended in water and then partitioned successively with petroleum ether, ethyl acetate, and *n*-BuOH.

The ethyl acetate-soluble fraction (95 g) was subjected to silica gel column chromatography using gradient mixture of CHCl_3_-MeOH (100:0 → 0:100, *v/v*) as eluent to afford 10 major fractions (Fr.1–Fr.10).

Fr.2 (30.5 g) was further separated on a silica gel column (petroleum ether-EtOAc, 100:0 → 0:100, *v/v*) to give six subfractions Fr.2A–Fr.2F. Fr.2B (1.2 g) was purified by a Sephadex LH-20 column (CHCl_3_-MeOH, 1:1) followed by semipreparative HPLC (CH_3_CN-H_2_O, 35:65, *v/v*) to yield compounds **2** (5.0 mg) and **4** (8.0 mg). Then, Fr.2D (15.0 g) was separated over an ODS column (MeOH-H_2_O, 20:80 → 100:0) to afford six subfractions (Fr.2D-1–Fr.2D-6). Fr.2D-2 (3.0 g) was subsequently purified by semipreparative HPLC (MeOH-H_2_O, 30:70, *v/v*) to give compounds **1** (14.5 mg) and **11** (8.4 mg), and Fr.2D-4 (1.5 g) was also purified by preparative HPLC (MeOH-H_2_O, 35:65, *v/v*) to afford compound **3** (6.0 mg).

Fr.3 (5.0 g) was applied to a Sephadex LH-20 column (MeOH) and gave five subfractions Fr.3A–Fr.3E. Furthermore, Fr.3B was further subjected to preparative HPLC (MeOH-H_2_O, 32:68, *v*/*v*) to give compound **12** (45.0 mg).

Fr. 6 (20.0 g) was purified over an ODS column using MeOH-H_2_O (20:80 → 100:0, *v/v*) as eluent to afford eight subfractions (Fr.6A–Fr.6H). Fr.6B (8.0 g) was subjected to a Sephadex LH-20 column (MeOH) to yield five subfractions (Fr.6B-1–Fr.6B-5). Fr.6B-2 (3.0 g) was purified by semipreparative HPLC (CH_3_CN-H_2_O, 18:82, *v/v*) to give compounds **5** (7.4 mg), **6** (10.0 mg), **7** (40.5 mg), and **13** (1.5 g), respectively. Fr.6B-4 (500 mg) was purified by preparative HPLC (CH_3_CN-H_2_O, 18:82, *v/v*) to give compounds **8** (25.3 mg), **9** (5.2 mg), and **10** (5.1 mg).

Fr.9 (3.5 g) was applied to a Sephadex LH-20 column (MeOH) to obtain four subfractions Fr.9A–Fr.9D. Then, Fr.9C (1.2 g) was further subjected to preparative HPLC separation (CH_3_CN-H_2_O, 12:88, *v/v*) to afford compound **14** (80 mg).

### 3.4. Compounds Characterization

Longifolactone G (**1**): colorless needles (MeOH); mp 290–291 °C; [*α*${]}_{D}^{25}$ +58.0 (*c* 0.55, MeOH); UV (CH_3_CN) *λ*_max_ (log *ε*): 241 (3.81) nm; IR (KBr) *v*_max_ 3455, 2957, 1730, 1667, 1432, 1379, 1348, 1229, 1198, 1115, 1017, 973, 878 cm^−^^1^; ^1^H and ^13^C NMR spectral data, see Tables S1 and S2; HR-ESI-MS *m/z* 395.1696 [M + H]^+^ (calcd for C_20_H_27_O_8_, 395.1700).

Longifolactone H (**2**): colorless needles (MeOH); mp 225–226 °C; [*α*${]}_{D}^{25}$ −6.2 (*c* 0.34, MeOH); UV (CH_3_CN) *λ*_max_ (log *ε*): 241 (4.01) nm; IR (KBr) *v*_max_ 3432, 2941, 1725, 1662, 1380, 1262, 1122, 998, 819, 564 cm^−^^1^; ^1^H and ^13^C NMR spectral data, see Tables S1 and S2; HR-ESI-MS *m/z* 433.1832 [M + Na]^+^ (calcd for C_21_H_30_O_8_Na, 433.1833).

Longifolactone I (**3**): colorless needles (MeOH); mp 178–179 °C; [*α*${]}_{D}^{25}$ +56.0 (*c* 0.46, MeOH); UV (CH_3_CN) *λ*_max_ (log *ε*); 230 (3.97) nm; IR (KBr) *v*_max_: 3468, 3346, 2953, 2904, 2577, 1727, 1496, 1411, 1316, 1226, 1128, 1057, 1015, 963, 813, 712, 638 cm^−^^1^; ^1^H and ^13^C NMR spectral data, see Tables S1 and S2; HR-ESI-MS: *m/z* 371.1831 [M + Na]^+^ (calcd for C_20_H_28_O_5_Na, 371.1829).

Longifolactone J (**4**): colorless needles (MeOH); mp 255–256 °C; [α${]}_{D}^{25}$ +18.2 (c 0.29, MeOH); UV (CH_3_CN) λ_max_ (log *ε*): 241 (3.75) nm; IR (KBr) *v*_max_ 3450, 2943, 1726, 1656, 1434, 1379, 1347, 1261, 1196, 1123, 1063, 998, 959, 589, 523 cm^−1^; ^1^H and ^13^C NMR spectral data, see Tables S1 and S2; HR-ESI-MS *m/z* 401.1574 [M + Na]^+^ (calcd for C_20_H_26_O_7_Na, 401.1571).

Longifolactone K (**5**): colorless needles (MeOH-C_5_H_5_N); mp 266–267 °C; [α${]}_{D}^{25}$ −7.2 (c 0.31, MeOH); UV (CH_3_CN) λ_max_ (log ε): 240 (4.01) nm; IR (KBr) v_max_ 3429, 2944, 1726, 1660, 1435, 1381, 1261, 1122, 1064, 997, 964, 902, 819, 698, 449 cm^−1^; ^1^H and ^13^C NMR spectral data, see Tables S1 and S2; HR-ESI-MS m/z 401.1574 [M + Na]^+^ (calcd for C_20_H_26_O_7_Na, 401.1571).

Longifolactone L (**6**): colorless needles (MeOH); mp 265–266 °C, [*α*${]}_{D}^{25}$ +22.2 (*c* 0.92, MeOH); UV (CH_3_CN) *λ*_max_ (log *ε*): 195 (3.80) nm; IR (KBr) *v*_max_: 3468, 3354, 2951, 2904, 2719, 1729, 1499, 1389, 1314, 1226, 1125, 1055, 964, 814 cm^−^^1^; ^1^H and ^13^C NMR spectral data, see Tables S1 and S3; HR-ESI-MS *m/z* 435.1626 [M + Na]^+^ (calcd for C_20_H_28_O_9_Na, 435.1626).

Longifolactone M (**7**): colorless needles (MeOH); mp 300–301 °C; [*α*${]}_{D}^{25}$ +10.1 (*c* 0.70, MeOH); UV (CH_3_CN) *λ*_max_ (log *ε*): 198 (3.60) nm; IR (KBr) *v*_max_ 3304, 2886, 1737, 1654, 1507, 1456, 1426, 1332, 1281, 1234, 1081, 993, 917 cm^−1^; ^1^H and ^13^C NMR spectral data, see Tables S1 and S3; HR-ESI-MS *m/z* 435.1621 [M + Na]^+^ (calcd for C_20_H_28_O_9_Na, 435.1626).

Longifolactone N (**8**): colorless needles (MeOH); mp 248–249 °C, [*α*${]}_{D}^{25}$ +44.6 (*c* 0.67, MeOH); UV (CH_3_CN) *λ*_max_ (log *ε*): 240 (3.46) nm; IR (KBr) *v*_max_: 3495, 3324, 1739, 1664, 1491, 1394, 1252, 1155, 1108, 1066, 973, 823 cm^−^^1^; ^1^H and ^13^C NMR spectral data, see Tables S1 and S3; HR-ESI-MS *m/z* 431.1311 [M + Na]^+^ (calcd for C_20_H_24_O_9_Na, 431.1313).

Longifolactone O (**9**): colorless needles (MeOH); mp 245–246 °C, [*α*${]}_{D}^{25}$ +4.2 (*c* 3.07, MeOH); UV (CH_3_CN) *λ*_max_ (log *ε*): 198 (3.63) nm; IR (KBr) *v*_max_: 3307, 2915, 1737, 1659, 1489, 1434, 1389, 1192, 1156, 1104, 974 cm^−^^1^; ^1^H and ^13^C NMR spectral data, see Tables S1 and S3; HR-ESI-MS: *m/z* 433.1472 [M + Na]^+^ (calcd for C_20_H_26_O_9_Na, 433.1469).

Longifolactone P (**10**): colorless needles (MeOH); mp 265–266 °C; [*α*${]}_{D}^{25}$ −1.3 (*c* 0.15, MeOH); UV (CH_3_CN) *λ*_max_ (log *ε*): 196 (3.70) nm; IR (KBr) *v*_max_: 3491, 3305, 1739, 1661, 1389, 1250, 1154, 1108, 1064, 975, 835 cm^−^^1^; ^1^H and ^13^C NMR spectral data, see Tables S1 and S3; HR-ESI-MS *m/z* 433.1470 [M + Na]^+^ (calcd for C_20_H_26_O_9_Na, 433.1469).

Chaparrolide (**11**): colorless needles (MeOH); mp 184–185 °C; [*α*${]}_{D}^{25}$ +15.6 (*c* 0.11, MeOH); UV (CH_3_CN) *λ*_max_ (log *ε*): 196 (3.94) nm; IR (KBr) *v*_max_ 3484, 3369, 2961, 1720, 1381, 1242, 1098, 1054, 982, 810 cm^−^^1^; ^1^H and ^13^C NMR spectral data, see Table S4 in Supplementary Materials; HR-ESI-MS *m/z* 389.1940 [M + Na]^+^ (calcd for C_20_H_30_O_6_Na, 389.1935).

15*β*-Hydroxyklaineanone (**12**): colorless needles (MeOH); mp 223–224 °C; [α${]}_{D}^{25}$ +6.3 (c 1.73, MeOH); UV (CH_3_CN) *λ*_max_ (log *ε*): 242 (4.05) nm; IR (KBr) *v*_max_ 3460, 2950, 1733, 1671, 1436, 1378, 1259, 1124, 1068, 1000, 958, 902, 814, 697, 633 cm^−1^; ^1^H and ^13^C NMR spectral data, see Table S4 in Supplementary Materials; HR-ESI-MS *m/z* 403.1734 [M + Na]^+^ (calcd for C_20_H_28_O_7_Na, 403.1727).

14,15*β*-Dihydroxyklaineanone (**13**): colorless needles (MeOH); mp 266–267 °C; [*α*${]}_{D}^{25}$ +54.3 (*c* 0.51, MeOH); UV (CH_3_CN) *λ*_max_ (log *ε*): 241 (4.05) nm; IR (KBr) *v*_max_ 3425, 2945, 1726, 1660, 1435, 1381, 1344, 1262, 1122, 1064, 998, 964, 902, 818, 698 cm^−1^; ^1^H and ^13^C NMR spectral data, see Table S4 in Supplementary Materials; HR-ESI-MS *m/z* 419.1674 [M + Na]^+^ (calcd for C_20_H_28_O_8_Na, 419.1676).

Eurycomanone (**14**): colorless needles (MeOH); mp 285–286 °C; [*α*${]}_{D}^{25}$ +39.5 (*c* 0.65, MeOH); UV (CH_3_CN) *λ*_max_ (log *ε*): 241 (3.46) nm; IR (KBr) *v*_max_ 3402, 2981, 2880, 1736, 1676, 1622, 1504, 1435, 1312, 1231, 1121, 1056, 985, 826, 765 cm^−1^; ^1^H and ^13^C NMR spectral data, see Table S4 in Supplementary Materials; HR-ESI-MS *m/z* 431.1314 [M + Na]^+^ (calcd for C_20_H_24_O_9_Na, 431.1313).

### 3.5. X-ray Crystallographic Analyses

The crystal data of compounds **1**‒**10** were collected using an Oxford-Diffraction SuperNova diffractometer (Agilent Technologies, Yarnton, UK) with Cu K*α* radiation. The crystal structures were solved by direct methods using the SHELXS program (Sheldrick, 2019) [23], and refined by the SHELXL-2018 program (Sheldrick, 2019) [23] and full-matrix least-squares calculation. Crystal data of compounds **1**‒**10** in standard CIF format were deposited with the Cambridge Crystallographic Data Centre (CCDC 2,105,724 for **1**, CCDC 2,105,722 for **2**, CCDC 2,105,723 for **3**, CCDC 2,105,731 for **4**, and CCDC 2,105,730 for **5**, CCDC 2,105,729 for **6**, CCDC 2,105,727 for **7**, and CCDC 2,105,725 for **8**, CCDC 2,105,726 for **9**, and CCDC 2,105,728 for **10**).

Crystal data for compound **1** (M = 394.41 g/mol): orthorhombic, space group P2_1_2_1_2_1_, a = 7.17350(10) Å, b = 10.18810(10) Å, c = 24.5189(2) Å, β = 90°, V = 1791.95(3) Å^3^, Z = 4, T = 99.99(10) K, μ (Cu Kα) = 0.948 mm^−1^, D_c__alc_ = 1.462 g/cm^3^, 21,948 reflections measured (7.21° ≤ 2θ ≤ 147.054°), 3593 unique (R_int_ = 0.0301, R_sigma_ = 0.0140) which were used in all calculations. The final R_1_ was 0.0306 (I > 2σ (I)), and wR_2_ was 0.0807 (all data). Flack parameter = 0.06(4).

Crystal data for compound **2** (M = 410.45 g/mol): monoclinic, space group P2_1_, a = 8.0642(2) Å, b = 10.6239(3) Å, c = 11.9568(3) Å, β = 107.411(2)°, V = 977.44(5) Å^3^, Z = 2, T = 100.00(10) K, μ (Cu Kα) = 0.888 mm^−1^, D_c__alc_ = 1.395 g/cm^3^, 9576 reflections measured (7.75° ≤ 2θ ≤ 146.844°), 3595 unique (R_int_ = 0.0302, R_sigma_ = 0.0220) which were used in all calculations. The final R_1_ was 0.0353 (I > 2σ (I)), and wR_2_ was 0.0946 (all data). Flack parameter = 0.09(8).

Crystal data for compound **3** (M = 364.44 g/mol): monoclinic, space group P2_1_, a = 7.0067(2) Å, b = 13.4001(4) Å, c = 9.7581(3) Å, β = 90.421(2)°, V = 916.17(5) Å^3^, Z = 2, T = 293(2) K, μ (Cu Kα) = 0.795 mm^−1^, D_c__alc_ = 1.328 g/cm^3^, 8696 reflections measured (9.062° ≤ 2θ ≤ 147.176°), 3440 unique (R_int_ = 0.0639, R_sigma_ = 0.0435) which were used in all calculations. The final R_1_ was 0.0355 (I > 2σ (I)), and wR_2_ was 0.0908 (all data). Flack parameter = 0.09(9).

Crystal data for compound **4** (M = 378.41 g/mol): orthorhombic, space group P2_1_2_1_2_1_, a = 9.63490(10) Å, b = 12.0407(2) Å, c = 15.6703(2) Å, β = 90°, V = 1817.93(4) Å^3^, Z = 4, T = 100.00(10) K, μ (Cu Kα) = 0.868 mm^−1^, D_c__alc_ = 1.383 g/cm^3^, 14,343 reflections measured (9.262° ≤ 2θ ≤ 146.826°), 3607 unique (R_int_ = 0.0285, R_sigma_ = 0.0201) which were used in all calculations. The final R_1_ was 0.0284 (I > 2σ (I)), and wR_2_ was 0.0746 (all data). Flack parameter = −0.05(6).

Crystal data for compound **5** (M = 457.51 g/mol): monoclinic, space group I2, a = 7.84600(10) Å, b = 12.85380(10) Å, c = 22.0124(2) Å, β = 95.8390(10)°, V = 2208.45(4) Å^3^, Z = 4, T = 100.00(10) K, μ (Cu Kα) = 0.828 mm^−1^, D_c__alc_ = 1.376 g/cm^3^, 20,973 reflections measured (7.976° ≤ 2θ ≤ 147.044°), 4407 unique (R_int_ = 0.0497, R_sigma_ = 0.0283) which were used in all calculations. The final R_1_ was 0.0334 (I > 2σ (I)), and wR_2_ was 0.0884 (all data). Flack parameter = 0.05(7).

Crystal data for compound **6** (M = 466.47 g/mol): orthorhombic, space group P2_1_2_1_2_1_, a = 13.8037(6) Å, b = 12.1850(6) Å, c = 12.0498(5) Å, β = 90°, V = 2026.75(16) Å^3^, Z = 4, T = 113(20) K, μ (Cu Kα) = 1.079 mm^−1^, D_c__alc_ = 1.529 g/cm^3^, 7421 reflections measured (7.336° ≤ 2θ ≤ 147.508°), 3904 unique (R_int_ = 0.0401, R_sigma_ = 0.0517) which were used in all calculations. The final R_1_ was 0.0503 (I > 2σ (I)), and wR_2_ was 0.1464 (all data). Flack parameter = −0.04(9).

Crystal data for compound **7** (M = 430.44 g/mol): orthorhombic, space group P2_1_2_1_2_1_, a = 6.94750(10) Å, b = 9.86740(10) Å, c = 28.8229(4) Å, β = 90°, V = 1975.92(4) Å^3^, Z = 4, T = 100.00(10) K, μ (Cu Kα) = 0.983 mm^−1^, D_c__alc_ = 1.447 g/cm^3^, 22,539 reflections measured (6.132° ≤ 2θ ≤ 147.044°), 3946 unique (R_int_ = 0.0695, R_sigma_ = 0.0368) which were used in all calculations. The final R_1_ was 0.0372 (I > 2σ (I)), and wR_2_ was 0.1016 (all data). Flack parameter = −0.12(9).

Crystal data for compound **8** (M = 480.45 g/mol): orthorhombic, space group P2_1_2_1_2_1_, a = 7.02070(10) Å, b = 13.3311(2) Å, c = 22.4885(3) Å, β = 90°, V = 2104.78(5) Å^3^, Z = 4, T = 100.01(10) K, μ (Cu Kα) = 1.097 mm^−1^, D_c__alc_ = 1.516 g/cm^3^, 20,217 reflections measured (7.71° ≤ 2θ ≤ 147.848°), 4187 unique (R_int_ = 0.0429, R_sigma_ = 0.0296) which were used in all calculations. The final R_1_ was 0.0282 (I > 2σ (I)), and wR_2_ was 0.0734 (all data). Flack parameter = −0.01(6).

Crystal data for compound **9** (M = 410.41 g/mol): monoclinic, space group C2, a = 17.7027(6) Å, b = 8.4150(3) Å, c = 12.3274(3) Å, β = 99.070(3)°, V = 1813.43(10) Å^3^, Z = 4, T = 100.00(10) K, μ (Cu Kα) = 1.004 mm^−1^, D_c__alc_ = 1.503 g/cm^3^, 16,975 reflections measured (7.262° ≤ 2θ ≤ 147.362°), 3635 unique (R_int_ = 0.0514, R_sigma_ = 0.0313) which were used in all calculations. The final R_1_ was 0.0388 (I > 2σ (I)), and wR_2_ was 0.1080 (all data). Flack parameter = 0.10(9).

Crystal data for compound **10** (M = 428.42 g/mol): orthorhombic, space group P2_1_2_1_2_1_, a = 7.2511(2) Å, b = 10.6856(3) Å, c = 24.0611(9) Å, β = 90°, V = 1864.31(10) Å^3^, Z = 4, T = 100.00(10) K, μ (Cu Kα) = 1.042 mm^−1^, D_c__alc_ = 1.526 g/cm^3^, 17,396 reflections measured (7.348° ≤ 2θ ≤ 147.984°), 3693 unique (R_int_ = 0.0511, R_sigma_ = 0.0405) which were used in all calculations. The final R_1_ was 0.0375 (I > 2σ (I)), and wR_2_ was 0.0896 (all data). Flack parameter = 0.07(10).

### 3.6. Cell Proliferation Assay

HL-60 and K562 cells were cultured in 96-well plates and incubated at 37 °C, 5% CO_2_ incubator. After incubation for 24 h, the cell supernatants were discarded and supplemented with cell culture medium containing compounds at different concentrations. At 48 h after incubation, the cell supernatants in each well were removed and replaced with 100 μL culture medium containing 10 μL of cell counting kit-8 (Sigma-Aldrich, St. Louis, MO, USA), followed by incubation at 37 °C, 5% CO_2_ for 2 h. The absorbance at 450 nm of the cells was measured using a Plate Reader. Cell proliferation was calculated according to the OD_450_ value in cells that were treated with or without compounds.

## 4. Conclusions

In summary, a further phytochemical study on the roots of the medicinal plant *Eurycoma longifolia* resulted in the isolation and characterization of 14 highly oxygenated C_20_ quassinoids, including 10 new ones (longifolactones G‒P, **1**–**10**). Structurally, compound **1** is the second member of a rare class of quassinoids featuring an unusual 2,5-dioxatricyclo[5.2.2.0^4,8^]undecane ring system. Compound **4** possesses a 14,15-epoxy functionality that is unprecedented in quassinoids, and compound **7** features an unusual α-oriented hydroxyl group at C-14. In addition, compounds **5**, **12**, **13**, and **14** showed potent anti-proliferation activities on two human leukemia cell lines, K562 and HL-60.

## Data Availability

All data supporting this study is available in the manuscript and the Supplementary Materials.

## Supplementary Materials

The following are available online, Detailed UV, IR, HR-ESI-MS, and NMR spectra of compounds **1**–**1****4**, as well as crystallographic data of compounds **1**‒**1****0** are available as Supplementary Materials.

## Sample Availability

Samples of the compounds **1**–**1****4** are available from the authors.
