# Supplementary material for: Quassinoids from the Roots of Eurycoma longifolia and Their Anti-Proliferation Activities
Source: Molecules. 2021 Sep 30;26(19):5939. doi: 10.3390/molecules26195939 (PMC8512324; doi:10.3390/molecules26195939)
Supplement: Supplementary file 1 [file molecules-26-05939-s001.zip › molecules-1380734-supplementary.pdf]

Supplementary Materials

Quassinoids from the Roots of *Eurycoma longifolia* and Their Anti-proliferation Activities

Wei-Qun Yang [1,2,3,†], Wei Tang [2,3,†], Xiao-Jun Huang [2,3], Jian-Guo Song [2,3], Yue-Yue Li [2,3], Yu Xiong [2,3], Chun-Lin Fan [2,3], Zhen-Long Wu [2,3,*], Ying Wang [2,3,*] and Wen-Cai Ye [2,3]

[1] School of Pharmaceutical Sciences, Guangzhou University of Chinese Medicine, Guangzhou 510006, China; yangwq_zzy@gzucm.edu.cn

[2] Center for Bioactive Natural Molecules and Innovative Drugs Research, College of Pharmacy, Jinan
University, Guangzhou 510632, China; tangv163@163.com (W.T.); zhyxiaohuang@163.com (X.J.H.); songjianguof403@126.com (J.G.S.); liyueyue7991@163.com (Y.Y.L.); xyu0218@163.com (Y.X.); jnuchunlin@163.com (C.L.F.); chywc@aliyun.com (W.C.Y.)

[3] Guangdong Province Key Laboratory of Pharmacodynamic Constituents of TCM and New Drugs Research, College of Pharmacy, Jinan University, Guangzhou 510632, China.

* Correspondence: zhenlongwu@jnu.edu.cn (Z.L.W.); wangying_cpu@163.com (Y.W.); Tel: +86-20-8522-1559 (Y.W.)

† These authors contributed equally to this work.

List of Supplementary Materials

Table S1. ^{13}C NMR spectroscopic data of compounds **1–10** (*δ* in ppm). S3

Table S2. ^{1}H NMR spectroscopic data of compounds **1–5** (*δ* in ppm, *J* in Hz). S4

Table S3. ^{1}H NMR spectroscopic data of compounds **6–10** (*δ* in ppm, *J* in Hz) S5

Table S4. ^{1}H and ^{13}C NMR spectroscopic data for **11–14** (*δ* in ppm, *J* in Hz) S6

Table S5. Anti-proliferation activities of quassinoids on human leukemia cell lines..S7

1. X-ray crystallographic analysis of **1** S8

2. X-ray crystallographic analysis of **2** S10

3. X-ray crystallographic analysis of **3** S13

4. X-ray crystallographic analysis of **4** S15

5. X-ray crystallographic analysis of **5** S17

6. X-ray crystallographic analysis of **6** S18

7. X-ray crystallographic analysis of **7** S20

8. X-ray crystallographic analysis of **8** S23

9. X-ray crystallographic analysis of **9** S25

10. X-ray crystallographic analysis of **10** S27

11. UV, IR, HR-ESI-MS, and NMR spectra of **1–14**..S29

Table S1. ^{13}C NMR spectroscopic data of compounds 1–10 (δ in ppm).

No.	**1** [a]	**2** [b]	**3** [b]	**4** [c]	**5** [c]	**6** [d]	**7** [a]	**8** [e]	**9** [a]	**10** [a]
1	83.5	84.0	77.5	82.0	84.6	85.5	84.1	83.2	82.8	79.2
2	199.7	200.4	132.7	198.2	197.2	71.6	72.3	197.0	72.5	70.6
3	125.6	125.1	130.2	124.4	125.0	44.3	127.6	126.6	127.4	127.3
4	163.3	165.2	145.8	164.4	163.2	147.4	134.9	161.7	134.2	133.9
5	47.9	44.6	39.7	43.5	42.5	42.5	42.8	43.3	42.5	47.6
6	31.7	24.8	26.9	25.7	25.1	27.1	25.5	27.4	27.5	32.7
7	111.0	84.4	83.6	77.4	82.2	75.7	73.9	74.8	75.4	79.2
8	52.5	43.3	37.6	38.8	42.6	53.6	51.4	47.8	47.9	47.4
9	47.8	43.5	43.7	45.1	48.0	48.8	46.6	53.4	53.7	53.3
10	48.8	49.1	44.7	47.6	46.2	42.2	42.3	49.2	46.0	41.5
11	69.5	74.4	69.6	73.7	210.2	111.0	111.1	74.9	75.3	75.5
12	85.5	75.8	75.9	75.4	82.4	80.0	81.8	176.4	176.6	176.2
13	29.9	28.6	32.0	29.4	36.2	42.8	33.3	54.0	54.2	55.8
14	82.9	63.2	46.1	67.9	52.4	76.9	78.6	79.9	79.9	81.2
15	82.7	102.5	30.6	52.9	67.3	72.3	74.2	69.0	69.3	71.6
16	171.5	174.5	171.8	168.4	174.5	175.3	173.0	174.9	175.1	173.8
18	13.0	16.0	15.2	9.0	16.2	14.5	10.8	12.6	12.8	14.3
19	12.9	11.9	11.4	11.9	9.9	12.3	10.7	9.2	9.5	16.3
29	21.6	22.5	111.3	22.7	22.6	110.1	21.9	22.7	21.8	22.2
30	12.8	23.1	22.5	20.6	23.1	67.8	72.2	68.7	69.0	71.7
1′		52.7								

[a] Measured in C_5D_5N, 125 MHz for ^{13}C NMR. [b] Measured in C_5D_5N, 150 MHz for ^{13}C NMR. [c] Measured in $CDCl_3$, 100 MHz for ^{13}C NMR. [d] Measured in C_5D_5N, 75 MHz for ^{13}C NMR. [e] Measured in C_5D_5N, 100 MHz for ^{13}C NMR.

Table S2. ^{1}H NMR spectroscopic data of compounds 1–5 (δ in ppm, J in Hz) [a].

No.	**1** [b]	**2** [c]	**3** [c]	**4** [d]	**5** [d]
1	4.00, s	4.20, s	4.34, s	4.01, s	3.98, s
2			5.76, d (9.6)		
3	6.07, br s	6.11, s	6.31, d (9.6)	6.08, br s	6.06, br s
5	2.92, m	3.20, d (13.0)	2.75	2.89, d (11.8)	2.95, d (12.1)
6	α 2.59, dd (14.0, 4.0)	α 2.29, dt (14.6, 3.2)	α 1.96, m	α 2.23, dt (14.8, 3.0)	α 2.23, dt (14.5, 3.3)
	β 2.24, t (14.0)	β 1.86, m	β 2.09	β 2.01	β 2.03, m
7		3.97, m	4.20, m	4.34, t (3.0)	4.50, m
9	2.11, d (2.3)	2.76, br s	2.35, d (10.3)	2.04	3.72, s
11	5.45, m	5.72	4.17	4.84, br s	
12	4.72, dd (4.6, 1.8)	4.10, m	4.17	3.92, m	3.94, d (2.7)
13	3.17, qd (7.2, 1.8)	2.94	2.09	2.83, qd (7.0, 2.3)	2.37, m
14		2.95	1.57, m		1.81
15	5.13, s		α 4.06, dd (19.2, 11.6)	3.63, s	5.42, d (10.1)
			β 2.76		
18	1.34, d (7.2)	1.48, d (6.5)	1.17, d (7.0)	0.89, d (7.0)	1.33, d (7.3)
19	1.39, s	1.38, s	1.10, s	1.11, s	1.22, s
29	1.70, s	1.77, s	a 5.00, s	1.96, s	1.96, s
			b 4.87, s		
30	1.96, s	1.77, s	1.11, s	1.58, s	1.17, s
1′		3.83, s			
11-OH	6.89, d (5.7)				
14-OH	7.05, s				

[a] Overlapped signals were reported without designating multiplicity. [b] Measured in C_5D_5N, 500 MHz for ^{1}H NMR. [c] Measured in C_5D_5N, 600 MHz for ^{1}H NMR. [d] Measured in $CDCl_3$, 400 MHz for ^{1}H NMR.

Table S3. **^1H NMR spectroscopic data of compounds 6–10 (δ in ppm, *J* in Hz)** [a].

No.	**6** [b]	**7** [c]	**8** [d]	**9** [c]	**10** [c]
1	3.88, d (8.6)	4.03	4.47, s	4.03, d (7.5)	4.12, d (7.8)
2	4.10, m	4.63, br s		4.55	4.47
3	α 2.38, t (11.9)	5.81, br s	6.10, s	5.76, br s	5.77, s
	β 2.87				
5	2.74	2.68, d (13.7)	3.17, d (12.5)	2.76, d (13.6)	2.30
6	2.13	α 2.23, dt (15.0, 2.1)	α 2.36, d (15.2)	α 2.20, dt (15.1, 2.8)	α 2.09, m
		β 2.05, td (15.0, 13.7)	β 2.22, m	β 2.04, ddd (15.1, 13.6, 2.8)	β 2.33, m
7	5.23, s	5.10, d (4.6)	4.65, br s	4.55	4.47
9	3.34, s	3.64, s	3.33, s	3.02, s	3.40, s
12	4.16, d (3.6)	4.20, br s			
13	2.90	3.08, m	2.95, q (7.3)	2.92, q (7.5)	3.01, q (7.4)
15	5.61, s	4.97, s	5.44, s	5.40, s	5.26, s
18	1.85, d (7.0)	1.32, d (6.9)	1.74, d (7.3)	1.72, d (7.5)	1.87, d (7.4)
19	1.67, s	1.67, s	1.33, s	1.45, s	1.50, s
29	a 4.98, s	1.66, s	1.80, s	1.63, s	1.72, s
	b 4.74, s				
30	a 4.67, d (8.8)	a 4.87, dd (9.6, 1.7)	a 5.63, d (12.2)	a 5.63, d (12.2)	a 5.50, d (11.3)
	b 4.10, d (8.8)	b 4.02	b 4.38, d (12.2)	b 4.37, d (12.2)	b 4.53, d (11.3)
14-OH		5.55, s		7.77, s	8.48, s
15-OH		8.88, d (5.1)			

[a] Overlapped signals were reported without designating multiplicity. [b] Measured in C_5D_5N, 300 MHz for ^1H NMR. [c] Measured in C_5D_5N, 500 MHz for ^1H NMR. [d] Measured in C_5D_5N, 400 MHz for ^1H NMR.

Table S4. ^{1}H and ^{13}C NMR spectroscopic data for 11–14 (δ in ppm, J in Hz)[a]

no.	11[b]		12[c]		13[d]		14[e]	
	δ_C	δ_H	δ_C	δ_H	δ_C	δ_H	δ_C	δ_H
1	86.7	3.27	84.0	3.87, d (2.4)	82.7	4.03, s	84.9	4.52, s
2	70.3	4.01, m	199.1		198.2		197.9	
3	43.6	α 1.21	124.0	6.02, br s	124.4	6.09, s	126.4	6.16, br s
		β 2.10, dt (12.8, 4.6)						
4	28.8	1.52, m	165.1		164.5		162.9	
5	44.4	1.37, m	42.5	2.86, d (12.1)	43.6	2.88, d (13.7)	42.5	3.26, d (12.5)
6	26.5	α 1.62, d (13.6)	25.1	α 2.01, m	26.1	α 2.29, dt (14.4, 2.9)	26.0	α 2.33, dt (15.1, 2.9)
		β 1.86		β 2.11, td (14.5, 12.4, 2.0)		β 2.10, m		β 2.02, td (15.1, 12.5, 2.9)
7	83.3	4.36, br s	81.9	4.24, s	81.5	4.63, t (3.0)	72.1	5.26, t (2.9)
8	41.1		37.3		48.2		52.9	
9	53.7	3.12, s	40.3	1.80, d (2.4)	45.0	2.11	48.1	3.82, s
10	42.9		47.9		43.1		46.3	
11	212.6		72.2	4.84, m	73.7	4.90, t (2.8)	109.9	
12	78.5	4.47, d (11.0)	75.3	3.35, m	77.0	3.90, t (2.6)	81.3	4.81, s
13	41.8	2.27, dq (11.0, 6.8)	26.3	2.34, ddd (7.3, 5.0, 2.4)	35.9	2.43, qd (7.3, 3.2)	148.4	
14	45.5	1.87	55.0	1.51, t (5.7)	75.9		79.8	
15	28.6	α 3.27	65.3	4.76, t (6.2)	71.2	5.36, s	76.2	5.67, s
		β 2.90, dd (18.7, 6.5)						
16	170.5		172.7		175.9		174.3	
18	15.7	1.23, d (6.8)	16.4	1.04, d (7.3)	12.5	1.23, d (7.3)	119.7	a 6.12, d (1.8)
								b 5.65, d (1.8)
19	12.3	1.59, s	11.7	1.07, s	12.4	1.18, s	10.8	1.62, s
29	20.1	0.77, d (6.3)	22.1	1.94, s	22.8	1.97, s	22.8	1.78, s
30	23.5	1.12, s	24.5	1.42, s	17.4	1.52, s	68.0	a 4.55, d (8.6)
								b 4.02, d (8.6)

[a] Overlapped signals were reported without designating multiplicity. [b] Measured in C_5D_5N, 600 MHz for ^{1}H NMR, 150 MHz for ^{13}C NMR. [c] Measured in DMSO-d_6, 300 MHz for ^{1}H NMR, 75 MHz for ^{13}C NMR. [d] Measured in $CDCl_3$, 400 MHz for ^{1}H NMR, 100 MHz for ^{13}C NMR. [e] Measured in C_5D_5N, 300 MHz for ^{1}H NMR, 75 MHz for ^{13}C NMR.

Table S5. Anti-proliferation activities of quassinoids on human leukemia cell lines.

Compound	K562 (IC_{50}, μM) [a]	HL-60 (IC_{50}, μM) [a]
**1**	>10	>10
**2**	>10	>10
**3**	>10	>10
**4**	>10	>10
**5**	6.33 ± 0.35	4.77 ± 0.47
**6**	>10	>10
**7**	>10	>10
**8**	>10	>10
**9**	>10	>10
**10**	>10	>10
**11**	>10	>10
**12**	4.31 ± 0.23	2.90 ± 0.35
**13**	3.23 ± 0.12	3.50 ± 0.57
**14**	8.20 ± 0.85	5.97 ± 0.55

[a] IC_{50} is the concentration of compounds that inhibited 50% cell proliferation.
Data are mean ± s.d., n = 3 biological replicates.

1. X-ray crystallographic analysis of 1

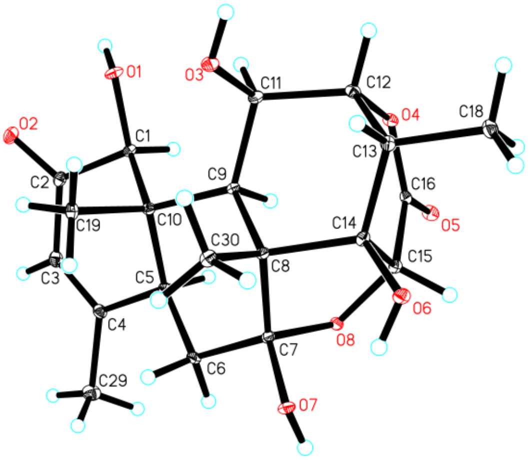

Figure S1. X-ray structure of **1**.

Colorless needles of **1** were obtained in methanol solvent. The crystal data was collected using an Oxford-Diffraction SuperNova diffractometer with Cu Kα radiation. The crystal structure was solved by direct methods using the SHELXS program and refined by the SHELXL-2018 program and full-matrix least-squares calculation. Crystallographic data for compound **1** have been deposited with the Cambridge Crystallographic Data Centre (CCDC 2105724).

Table S2. Crystal data and structure refinement for 1.

Empirical formula	$C_{20}H_{26}O_8$
Formula weight	394.41
Temperature/K	99.99(10)
Crystal system	orthorhombic
Space group	$P2_12_12_1$
a/Å	7.17350(10)

b/Å	10.18810(10)
c/Å	24.5189(2)
α/°	90
β/°	90
γ/°	90
Volume/$Å^3$	1791.95(3)
Z	4
ρ_{calc}g/cm^3	1.462
μ/mm^{-1}	0.948
F(000)	840.0
Crystal size/mm^3	0.14 × 0.13 × 0.12
Radiation	CuKα (λ = 1.54184)
2Θ range for data collection/°	7.21 to 147.054
Index ranges	-8 ≤ h ≤ 8, -12 ≤ k ≤ 12, -29 ≤ l ≤ 30
Reflections collected	21948
Independent reflections	3593 [R_{int} = 0.0301, R_{sigma} = 0.0140]
Data/restraints/parameters	3593/0/261
Goodness-of-fit on F^2	1.115
Final R indexes [I>=2σ (I)]	R_1 = 0.0306, wR_2 = 0.0806
Final R indexes [all data]	R_1 = 0.0307, wR_2 = 0.0807
Largest diff. peak/hole / e $Å^{-3}$	0.20/-0.41

Flack parameter	0.06(4)

2. X-ray crystallographic analysis of 2

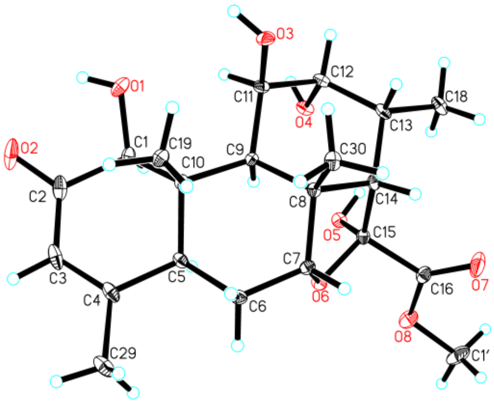

Figure S2. X-ray structure of **2**.

Colorless needles of **2** were obtained in methanol solvent. The crystal data was collected using an Oxford-Diffraction SuperNova diffractometer with Cu Kα radiation. The crystal structure was solved by direct methods using the SHELXS program, and refined by the SHELXL-2018 program and full-matrix least-squares calculation. Crystallographic data for compound **2** have been deposited with the Cambridge Crystallographic Data Centre (CCDC 2105722).

Table S3. Crystal data and structure refinement for 2.

Empirical formula	$C_{21}H_{30}O_8$
Formula weight	410.45
Temperature/K	100.00(10)
Crystal system	monoclinic
Space group	$P2_1$
a/Å	8.0642(2)
b/Å	10.6239(3)
c/Å	11.9568(3)
α/°	90
β/°	107.411(2)
γ/°	90
Volume/Å^3	977.44(5)
Z	2
ρ_{calc}g/cm^3	1.395
μ/mm^{-1}	0.888
F(000)	440.0
Crystal size/mm^3	0.14 × 0.12 × 0.11
Radiation	CuKα (λ = 1.54184)
2Θ range for data collection	7.75 to 146.844

Index ranges	$-9 \leq h \leq 10$, $-12 \leq k \leq 13$, $-14 \leq l \leq 14$
Reflections collected	9576
Independent reflections	3595 [R_{int} = 0.0302, R_{sigma} = 0.0220]
Data/restraints/parameters	3595/1/274
Goodness-of-fit on F^2	1.068
Final R indexes [I>=2σ (I)]	R_1 = 0.0353, wR_2 = 0.0943
Final R indexes [all data]	R_1 = 0.0355, wR_2 = 0.0946
Largest diff. peak/hole / e Å^{-3}	0.23/-0.21
Flack parameter	0.09(8)

3. X-ray crystallographic analysis of 3

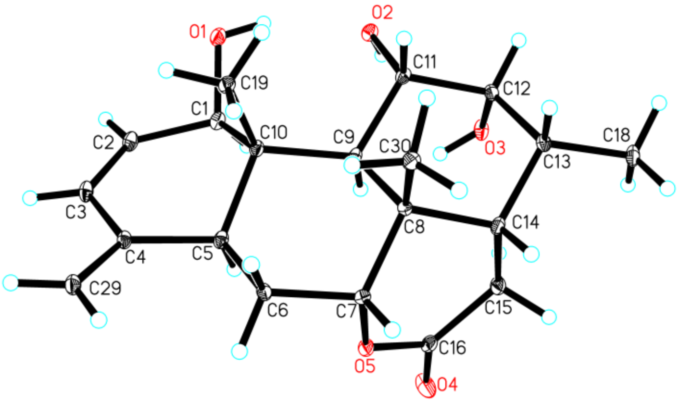

Figure S3. X-ray structure of **3**.

Colorless needles of **3** were obtained in methanol solvent. The crystal data was collected using an Oxford-Diffraction SuperNova diffractometer with Cu Kα radiation. The crystal structure was solved by direct methods using the SHELXS program, and refined by the SHELXL-2018 program and full-matrix least-squares calculation. Crystallographic data for compound **3** have been deposited with the Cambridge Crystallographic Data Centre (CCDC 2105723).

Table S4 Crystal data and structure refinement for 3.

Empirical formula	$C_{20}H_{30}O_6$
Formula weight	366.44
Temperature/K	293(2)
Crystal system	monoclinic
Space group	$P2_1$
a/Å	7.0067(2)
b/Å	13.4001(4)

c/Å	9.7581(3)
α/°	90
β/°	90.421(2)
γ/°	90
Volume/$Å^3$	916.17(5)
Z	2
ρ_{calc}g/cm^3	1.328
μ/mm^{-1}	0.795
F(000)	396.0
Crystal size/mm^3	? × ? × ?
Radiation	CuKα (λ = 1.54184)
2Θ range for data collection/°	9.062 to 147.176
Index ranges	-8 ≤ h ≤ 8, -16 ≤ k ≤ 16, -11 ≤ l ≤ 12
Reflections collected	8696
Independent reflections	3440 [R_{int} = 0.0639, R_{sigma} = 0.0435]
Data/restraints/parameters	3440/1/244
Goodness-of-fit on F^2	1.039
Final R indexes [I>=2σ (I)]	R_1 = 0.0355, wR_2 = 0.0891
Final R indexes [all data]	R_1 = 0.0373, wR_2 = 0.0908
Largest diff. peak/hole / e $Å^{-3}$	0.18/-0.26
Flack parameter	0.09(9)

4. X-ray crystallographic analysis of 4

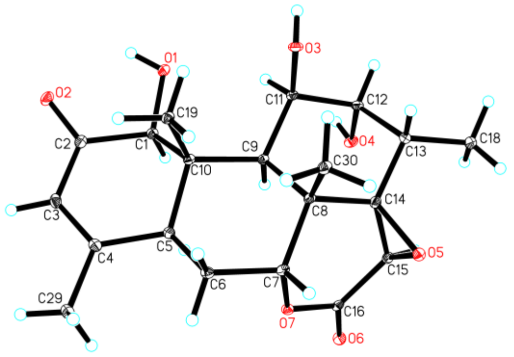

Figure S4. X-ray structure of **4**.

Colorless needles of **4** were obtained in methanol solvent. The crystal data was collected using an Oxford-Diffraction SuperNova diffractometer with Cu Kα radiation. The crystal structure was solved by direct methods using the SHELXS program, and refined by the SHELXL-2018 program and full-matrix least-squares calculation. Crystallographic data for compound **4** have been deposited with the Cambridge Crystallographic Data Centre (CCDC 2105731).

Table S5. Crystal data and structure refinement for 4.

Empirical formula	$C_{20}H_{26}O_7$
Formula weight	378.41
Temperature/K	100.00(10)
Crystal system	orthorhombic
Space group	$P2_12_12_1$
a/Å	9.63490(10)
b/Å	12.0407(2)

c/Å	15.6703(2)
α/°	90
β/°	90
γ/°	90
Volume/Å^3	1817.93(4)
Z	4
ρ_{calc}g/cm^3	1.383
μ/mm^{-1}	0.868
F(000)	808.0
Crystal size/mm^3	0.14 × 0.12 × 0.11
Radiation	CuKα (λ = 1.54184)
2Θ range for data collection	9.262 to 146.826
Index ranges	-11 ≤ h ≤ 11, -12 ≤ k ≤ 14, -19 ≤ l ≤ 19
Reflections collected	14343
Independent reflections	3607 [R_{int} = 0.0285, R_{sigma} = 0.0201]
Data/restraints/parameters	3607/0/251
Goodness-of-fit on F^2	1.042
Final R indexes [I>=2σ (I)]	R_1 = 0.0284, wR_2 = 0.0741
Final R indexes [all data]	R_1 = 0.0290, wR_2 = 0.0746
Largest diff. peak/hole / e Å^{-3}	0.22/-0.20
Flack parameter	-0.05(6)

5. X-ray crystallographic analysis of 5

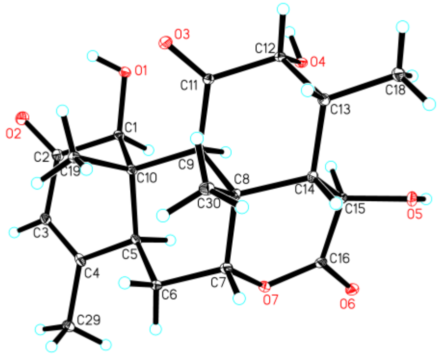

Figure S5. X-ray structure of **5**.

Colorless needles of **5** were obtained in methanol/pyridine mixed solvent. The crystal data was collected using an Oxford-Diffraction SuperNova diffractometer with Cu Kα radiation. The crystal structure was solved by direct methods using the SHELXS program, and refined by the SHELXL-2018 program and full-matrix least-squares calculation. Crystallographic data for compound **5** have been deposited with the Cambridge Crystallographic Data Centre (CCDC 2105730).

Table S6. Crystal data and structure refinement for 5.

Formula weight	457.51
Temperature/K	100.00(10)
Crystal system	monoclinic
Space group	I2
a/Å	7.84600(10)
b/Å	12.85380(10)
c/Å	22.0124(2)
α/°	90

β/°	95.8390(10)
γ/°	90
Volume/$Å^3$	2208.45(4)
Z	4
ρ_{calc}g/cm^3	1.376
μ/mm^{-1}	0.828
F(000)	976.0
Crystal size/mm^3	0.14 × 0.13 × 0.11
Radiation	CuKα (λ = 1.54184)
2Θ range for data collection/°	7.976 to 147.044
Index ranges	-9 ≤ h ≤ 9, -15 ≤ k ≤ 15, -27 ≤ l ≤ 27
Reflections collected	20973
Independent reflections	4407 [R_{int} = 0.0497, R_{sigma} = 0.0283]
Data/restraints/parameters	4407/1/305
Goodness-of-fit on F^2	1.029
Final R indexes [I>=2σ (I)]	R_1 = 0.0334, wR_2 = 0.0879
Final R indexes [all data]	R_1 = 0.0336, wR_2 = 0.0884
Largest diff. peak/hole / e $Å^{-3}$	0.22/-0.23
Flack parameter	0.05(7)

6. X-ray crystallographic analysis of 6

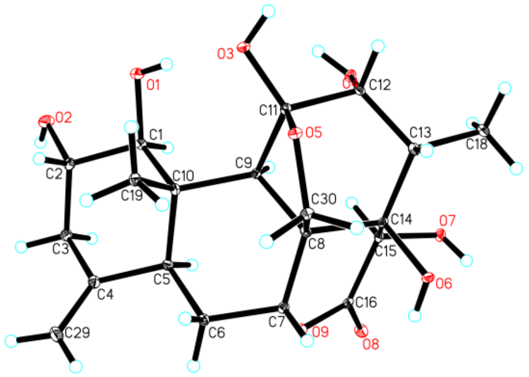

Figure S6. X-ray structure of **6**.

Colorless needles of **6** were obtained in methanol/pyridine mixed solvent. The crystal data was collected using an Oxford-Diffraction SuperNova diffractometer with Cu Kα radiation. The crystal structure was solved by direct methods using the SHELXS program, and refined by the SHELXL-2018 program and full-matrix least-squares calculation. Crystallographic data for compound **6** have been deposited with the Cambridge Crystallographic Data Centre (CCDC 2105729).

Table S7. Crystal data and structure refinement for 6.

Empirical formula	$C_{20}H_{34}O_{12}$
Formula weight	466.47
Temperature/K	113(20)
Crystal system	orthorhombic
Space group	$P2_12_12$
a/Å	13.8037(6)
b/Å	12.1850(6)
c/Å	12.0498(5)

α/°	90
β/°	90
γ/°	90
Volume/Å^3	2026.75(16)
Z	4
ρ_{calc}g/cm^3	1.529
μ/mm^{-1}	1.079
F(000)	1000.0
Crystal size/mm^3	? × ? × ?
Radiation	Cu Kα (λ = 1.54184)
2Θ range for data collection/°	7.336 to 147.508
Index ranges	-17 ≤ h ≤ 11, -14 ≤ k ≤ 14, -14 ≤ l ≤ 10
Reflections collected	7421
Independent reflections	3904 [R_{int} = 0.0401, R_{sigma} = 0.0517]
Data/restraints/parameters	3904/0/314
Goodness-of-fit on F^2	1.144
Final R indexes [I>=2σ (I)]	R_1 = 0.0503, wR_2 = 0.1441
Final R indexes [all data]	R_1 = 0.0530, wR_2 = 0.1464
Largest diff. peak/hole / e Å^{-3}	0.46/-0.45
Flack parameter	-0.04(9)

7. X-ray crystallographic analysis of 7

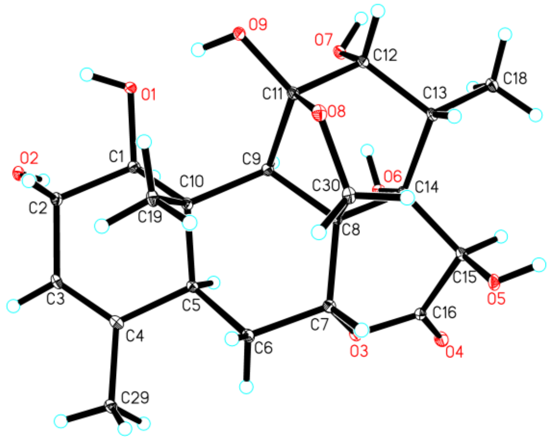

Figure S7. X-ray structure of **7**.

Colorless needles of **7** were obtained in methanol solvent. The crystal data was collected using an Oxford-Diffraction SuperNova diffractometer with Cu Kα radiation. The crystal structure was solved by direct methods using the SHELXS program, and refined by the SHELXL-2018 program and full-matrix least-squares calculation. Crystallographic data for compound **7** have been deposited with the Cambridge Crystallographic Data Centre (CCDC 2105727).

Table S8. Crystal data and structure refinement for 7.

Empirical formula	$C_{20}H_{30}O_{10}$
Formula weight	430.44
Temperature/K	100.00(10)
Crystal system	orthorhombic
Space group	$P2_12_12_1$
a/Å	6.94750(10)
b/Å	9.86740(10)

c/Å	28.8229(4)
α/°	90
β/°	90
γ/°	90
Volume/Å^3	1975.92(4)
Z	4
ρ_{calc}g/cm^3	1.447
μ/mm^{-1}	0.983
F(000)	920.0
Crystal size/mm^3	0.13 × 0.12 × 0.11
Radiation	CuKα (λ = 1.54184)
2Θ range for data collection/°	6.132 to 147.044
Index ranges	$-8 \le h \le 8$, $-12 \le k \le 12$, $-35 \le l \le 35$
Reflections collected	22539
Independent reflections	3946 [R_{int} = 0.0695, R_{sigma} = 0.0368]
Data/restraints/parameters	3946/0/283
Goodness-of-fit on F^2	1.059
Final R indexes [I>=2σ (I)]	R_1 = 0.0372, wR_2 = 0.0994
Final R indexes [all data]	R_1 = 0.0389, wR_2 = 0.1016
Largest diff. peak/hole / e Å^{-3}	0.28/-0.27
Flack parameter	-0.12(9)

8. X-ray crystallographic analysis of 8

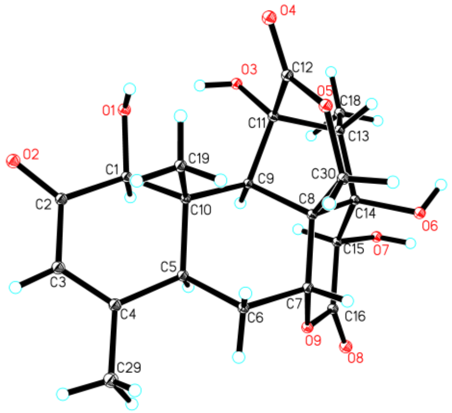

Figure S8. X-ray structure of **8**.

Colorless needles of **8** were obtained in methanol solvent. The crystal data was collected using an Oxford-Diffraction SuperNova diffractometer with Cu Kα radiation. The crystal structure was solved by direct methods using the SHELXS program, and refined by the SHELXL-2018 program and full-matrix least-squares calculation. Crystallographic data for compound **8** have been deposited with the Cambridge Crystallographic Data Centre (CCDC 2105725).

Table S9. Crystal data and structure refinement for 8.

Empirical formula	$C_{20}H_{32}O_{13}$
Formula weight	480.45
Temperature/K	100.01(10)
Crystal system	orthorhombic
Space group	$P2_12_12_1$
a/Å	7.02070(10)
b/Å	13.3311(2)

c/Å	22.4885(3)
α/°	90
β/°	90
γ/°	90
Volume/Å^3	2104.78(5)
Z	4
ρ_{calc}g/cm^3	1.516
μ/mm^{-1}	1.097
F(000)	1024.0
Crystal size/mm^3	0.9 × 0.7 × 0.5
Radiation	CuKα (λ = 1.54184)
2Θ range for data collection/°	7.71 to 147.848
Index ranges	-6 ≤ h ≤ 8, -16 ≤ k ≤ 16, -27 ≤ l ≤ 27
Reflections collected	20217
Independent reflections	4187 [R_{int} = 0.0429, R_{sigma} = 0.0296]
Data/restraints/parameters	4187/0/317
Goodness-of-fit on F^2	1.040
Final R indexes [I>=2σ (I)]	R_1 = 0.0282, wR_2 = 0.0723
Final R indexes [all data]	R_1 = 0.0298, wR_2 = 0.0734
Largest diff. peak/hole / e Å^{-3}	0.25/-0.26
Flack parameter	-0.01(6)

9. X-ray crystallographic analysis of 9

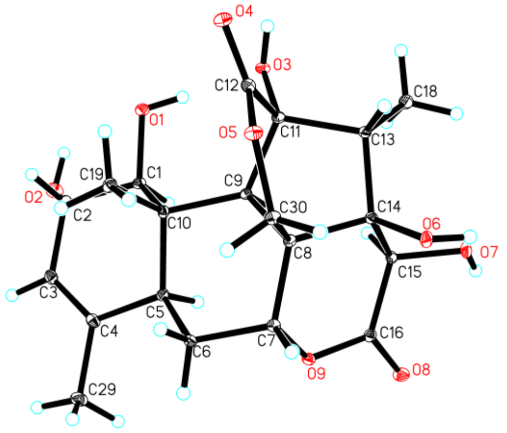

Figure S9. X-ray structure of **9**.

Colorless needles of **9** were obtained in methanol solvent. The crystal data was collected using an Oxford-Diffraction SuperNova diffractometer with Cu Kα radiation. The crystal structure was solved by direct methods using the SHELXS program, and refined by the SHELXL-2018 program and full-matrix least-squares calculation. Crystallographic data for compound **9** have been deposited with the Cambridge Crystallographic Data Centre (CCDC 2105726).

Table S10. Crystal data and structure refinement for 9.

Empirical formula	$C_{20}H_{26}O_9$
Formula weight	410.41
Temperature/K	100.00(10)
Crystal system	monoclinic
Space group	C2
a/Å	17.7027(6)
b/Å	8.4150(3)

c/Å	12.3274(3)
α/°	90
β/°	99.070(3)
γ/°	90
Volume/$Å^3$	1813.43(10)
Z	4
ρ_{calc}g/cm^3	1.503
μ/mm^{-1}	1.004
F(000)	872.0
Crystal size/mm^3	0.9 × 0.7 × 0.5
Radiation	CuKα (λ = 1.54184)
2Θ range for data collection/°	7.262 to 147.362
Index ranges	-21 ≤ h ≤ 21, -10 ≤ k ≤ 10, -15 ≤ l ≤ 15
Reflections collected	16975
Independent reflections	3635 [R_{int} = 0.0514, R_{sigma} = 0.0313]
Data/restraints/parameters	3635/1/270
Goodness-of-fit on F^2	1.149
Final R indexes [I>=2σ (I)]	R_1 = 0.0388, wR_2 = 0.1073
Final R indexes [all data]	R_1 = 0.0396, wR_2 = 0.1080
Largest diff. peak/hole / e $Å^{-3}$	0.67/-0.27
Flack parameter	0.10(9)

10. X-ray crystallographic analysis of 10

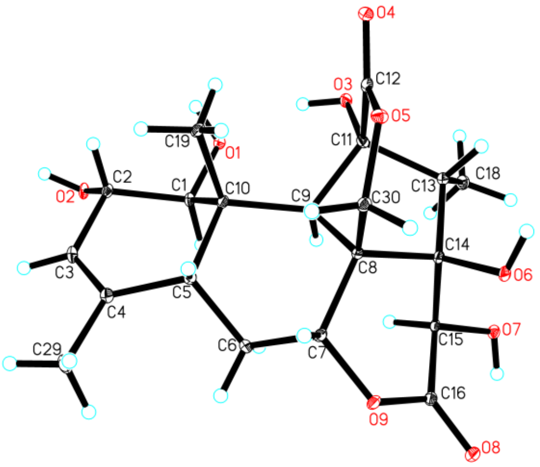

Figure S10. X-ray structure of **10**.

Colorless needles of **10** were obtained in methanol solvent. The crystal data was collected using an Oxford-Diffraction SuperNova diffractometer with Cu Kα radiation. The crystal structure was solved by direct methods using the SHELXS program, and refined by the SHELXL-2018 program and full-matrix least-squares calculation. Crystallographic data for compound **10** have been deposited with the Cambridge Crystallographic Data Centre (CCDC 2105728).

Table S11. Crystal data and structure refinement for 10.

Empirical formula	$C_{20}H_{28}O_{10}$
Formula weight	428.42
Temperature/K	100.00(10)
Crystal system	orthorhombic
Space group	$P2_12_12_1$
a/Å	7.2511(2)

b/Å	10.6856(3)
c/Å	24.0611(9)
α/°	90
β/°	90
γ/°	90
Volume/Å^3	1864.31(10)
Z	4
ρ_{calc}g/cm^3	1.526
μ/mm^{-1}	1.042
F(000)	912.0
Crystal size/mm^3	0.9 × 0.7 × 0.5
Radiation	CuKα (λ = 1.54184)
2Θ range for data collection/°	7.348 to 147.984
Index ranges	-8 ≤ h ≤ 8, -13 ≤ k ≤ 13, -29 ≤ l ≤ 29
Reflections collected	17396
Independent reflections	3693 [R_{int} = 0.0511, R_{sigma} = 0.0405]
Data/restraints/parameters	3693/0/282
Goodness-of-fit on F^2	1.082
Final R indexes [I>=2σ (I)]	R_1 = 0.0375, wR_2 = 0.0878
Final R indexes [all data]	R_1 = 0.0426, wR_2 = 0.0896
Largest diff. peak/hole / e Å^{-3}	0.21/-0.24

Flack parameter 0.07(10)

11. UV, IR, HR-ESI-MS, and NMR spectra of 1–14

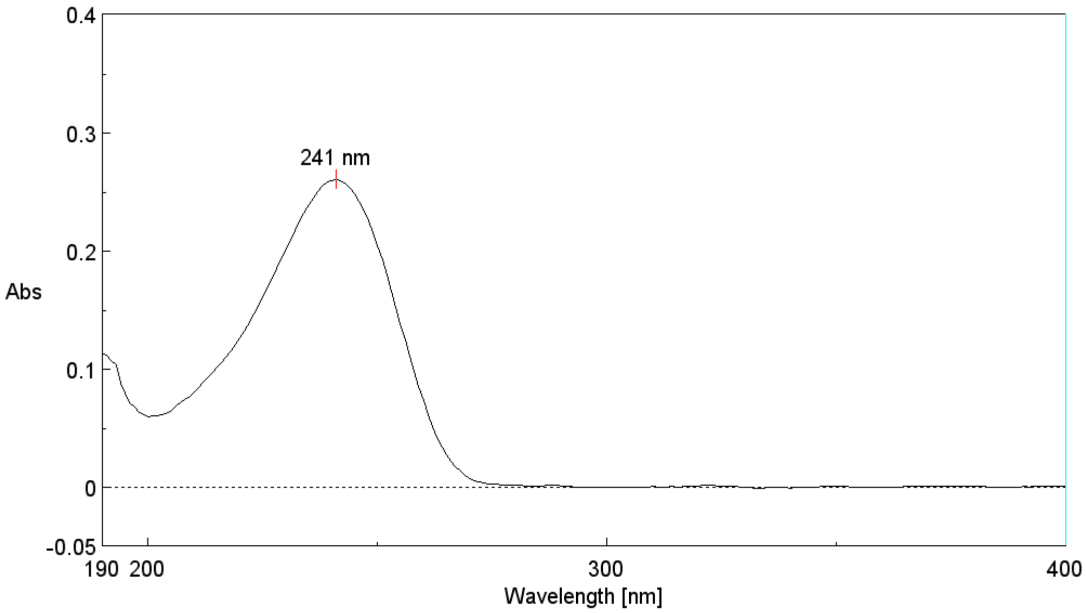

Figure S11. UV spectrum of compound **1**

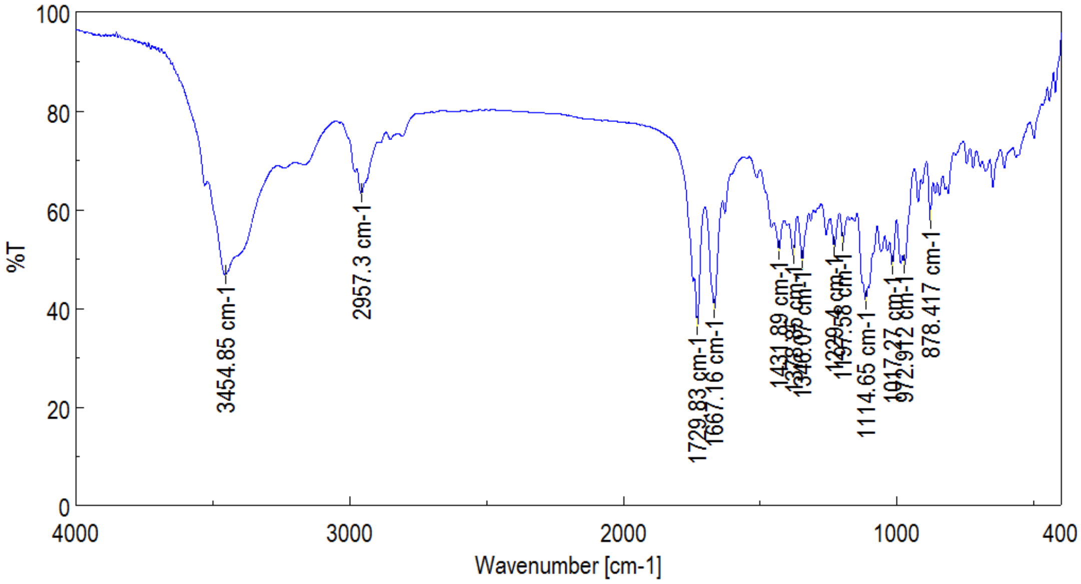

Figure S12. IR (KBr disc) spectrum of compound **1**

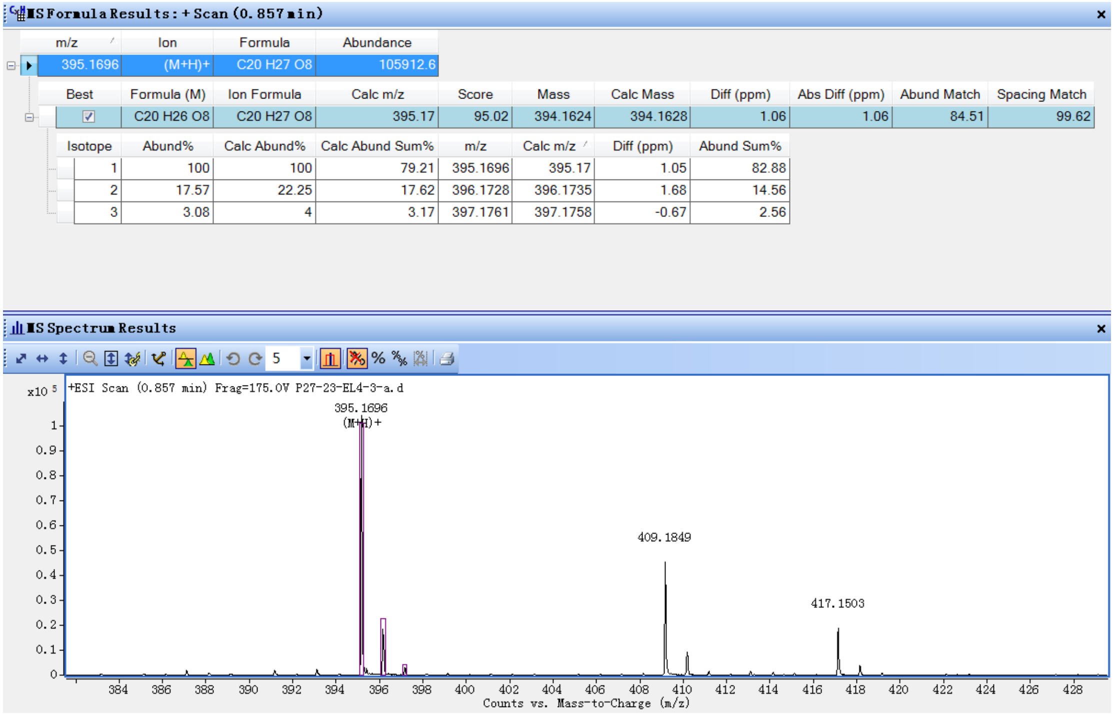

Figure S13. HR-ESI-MS spectrum of compound **1**

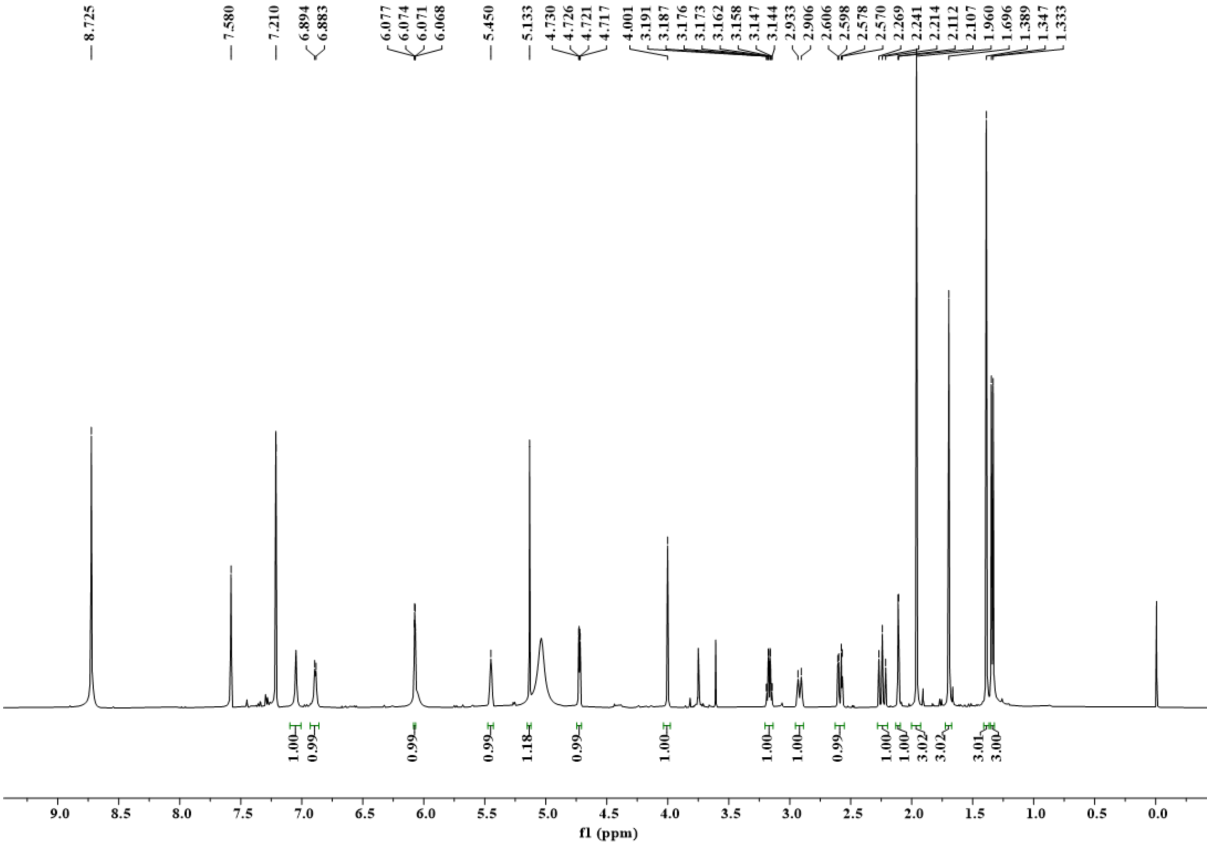

Figure S14. ^{1}H NMR spectrum of compound **1**

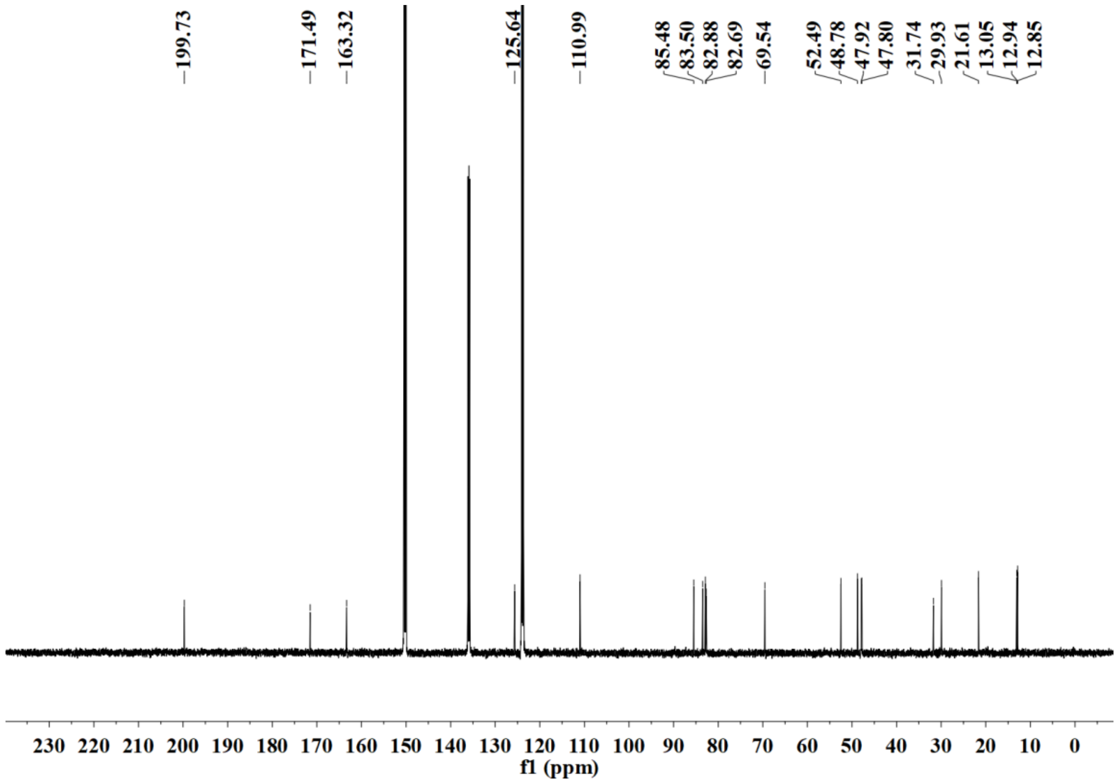

Figure S15. ^{13}C NMR spectrum of compound **1**

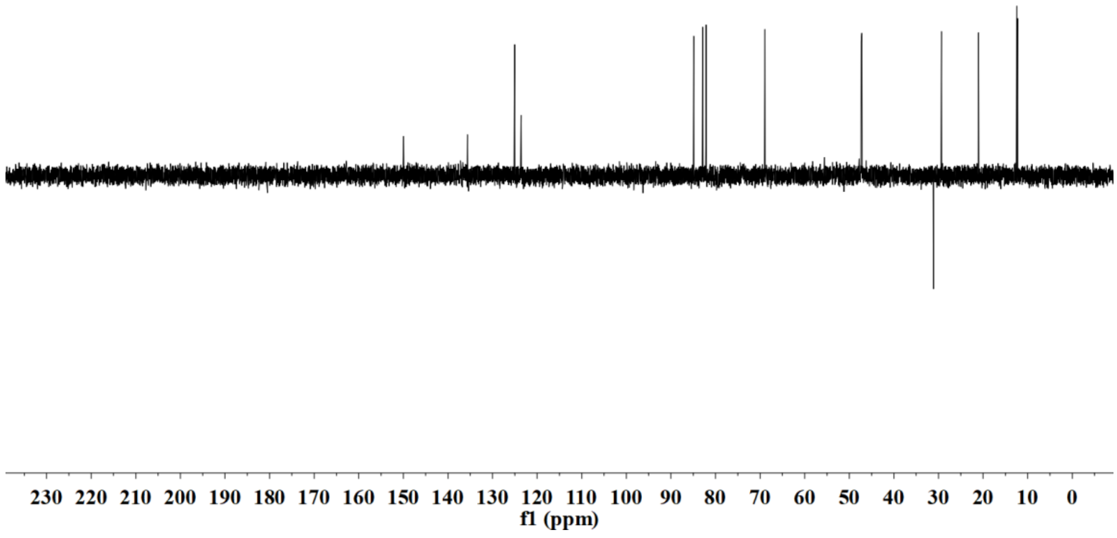

Figure S16. DEPT-135 spectrum of compound **1**

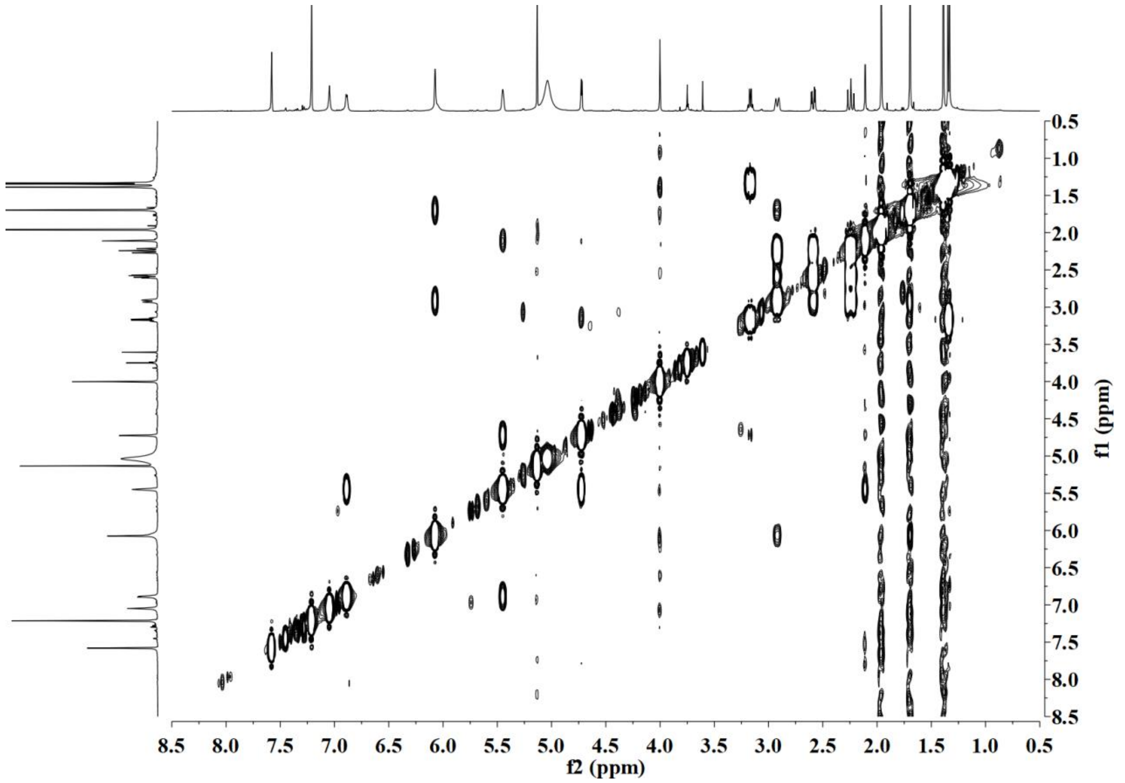

Figure S17. ^{1}H-^{1}H COSY spectrum of compound **1**

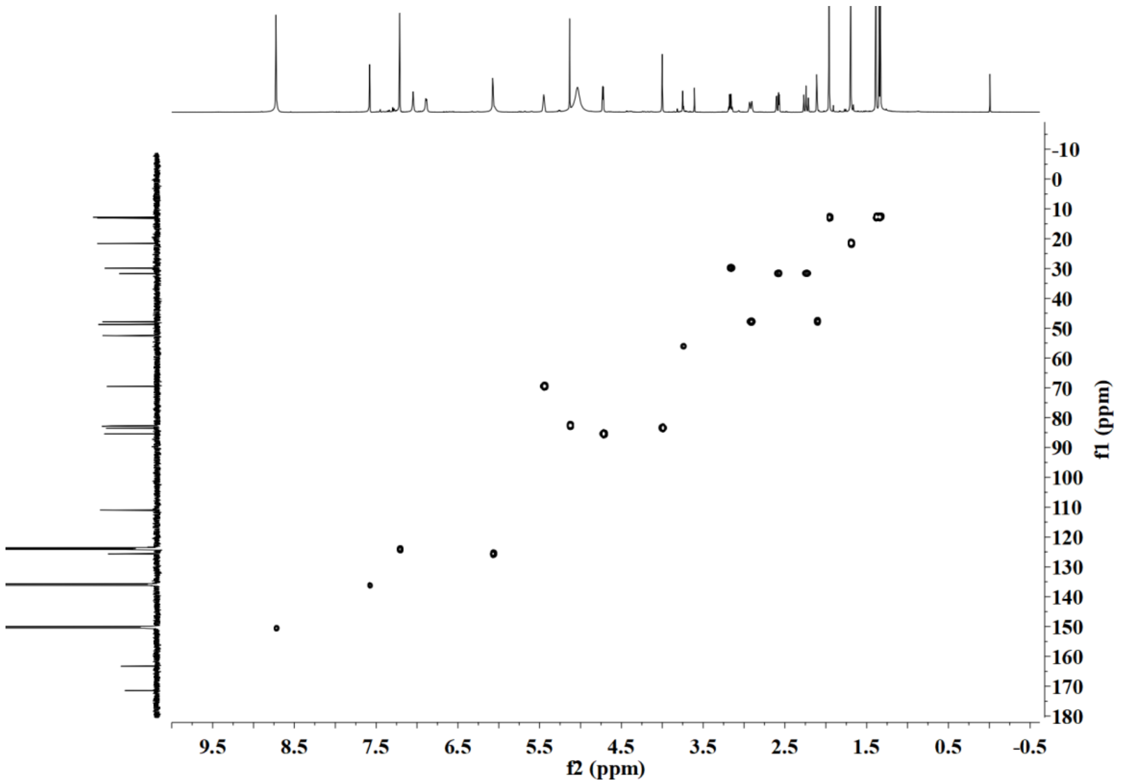

Figure S18. HSQC spectrum of compound **1**

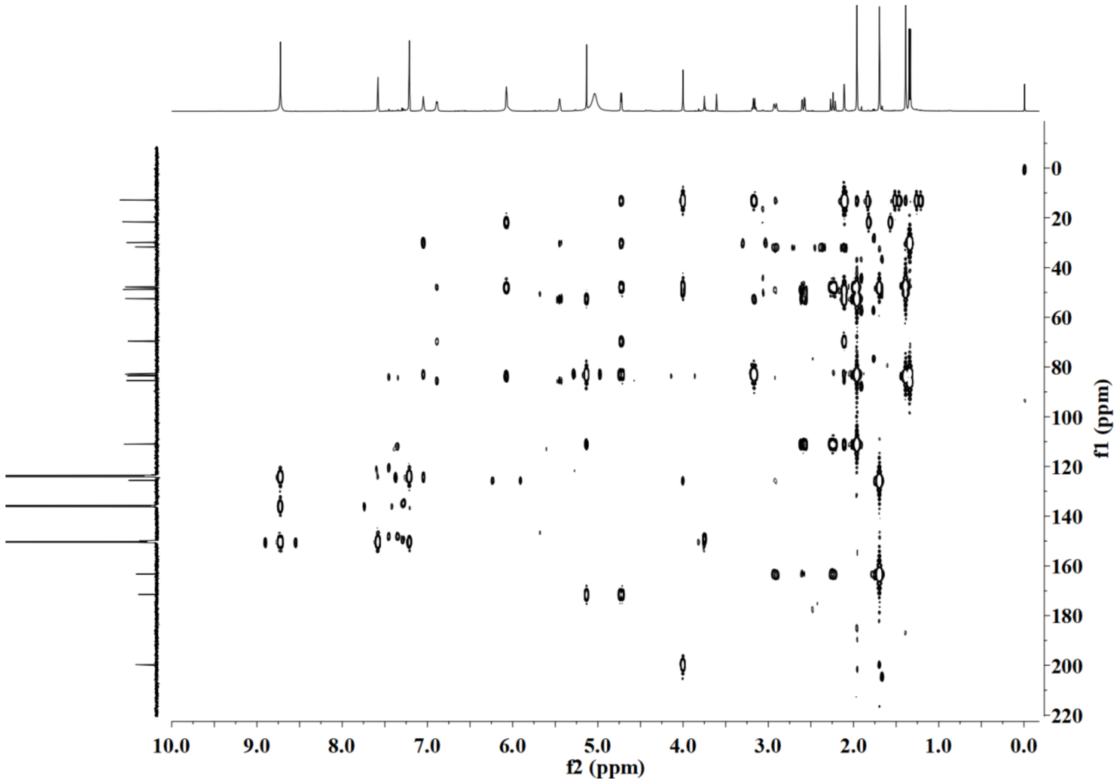

Figure S19. HMBC spectrum of compound **1**

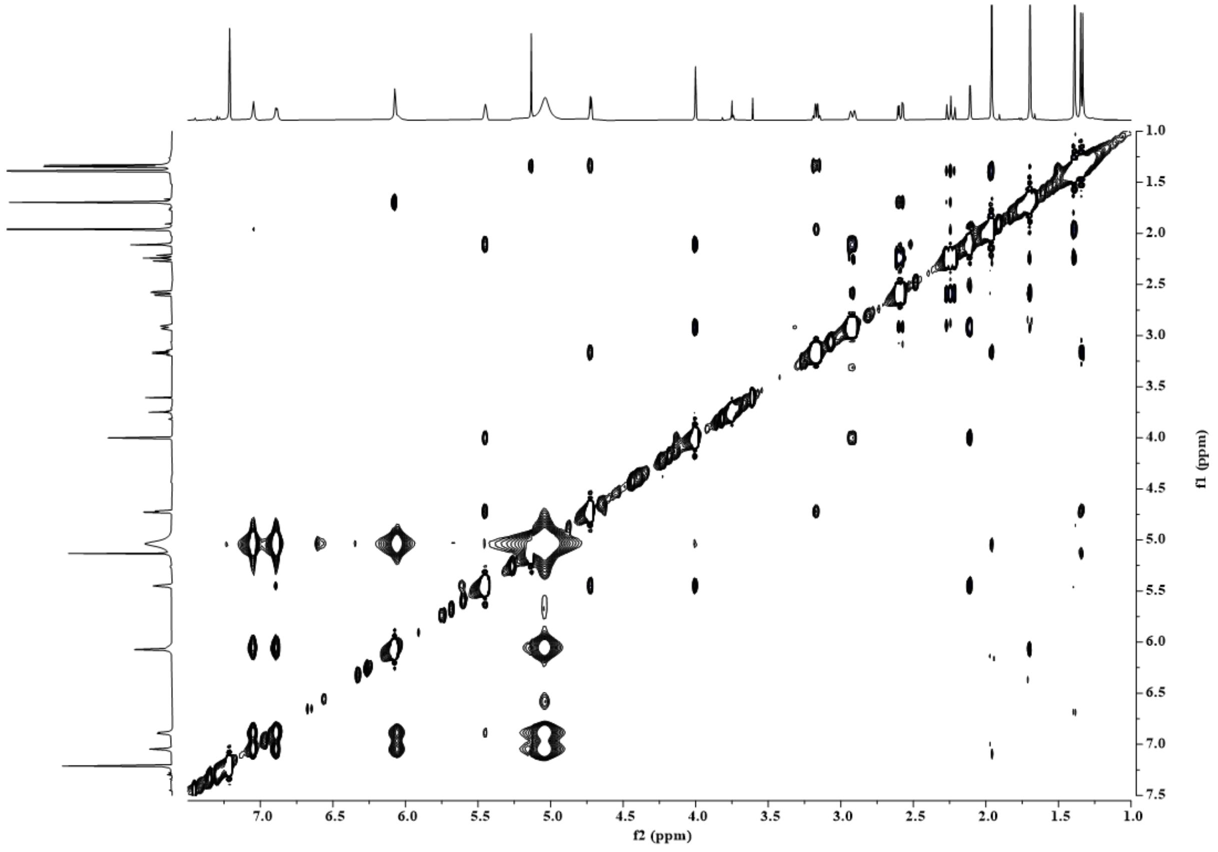

Figure S20. NOESY spectrum of compound **1**

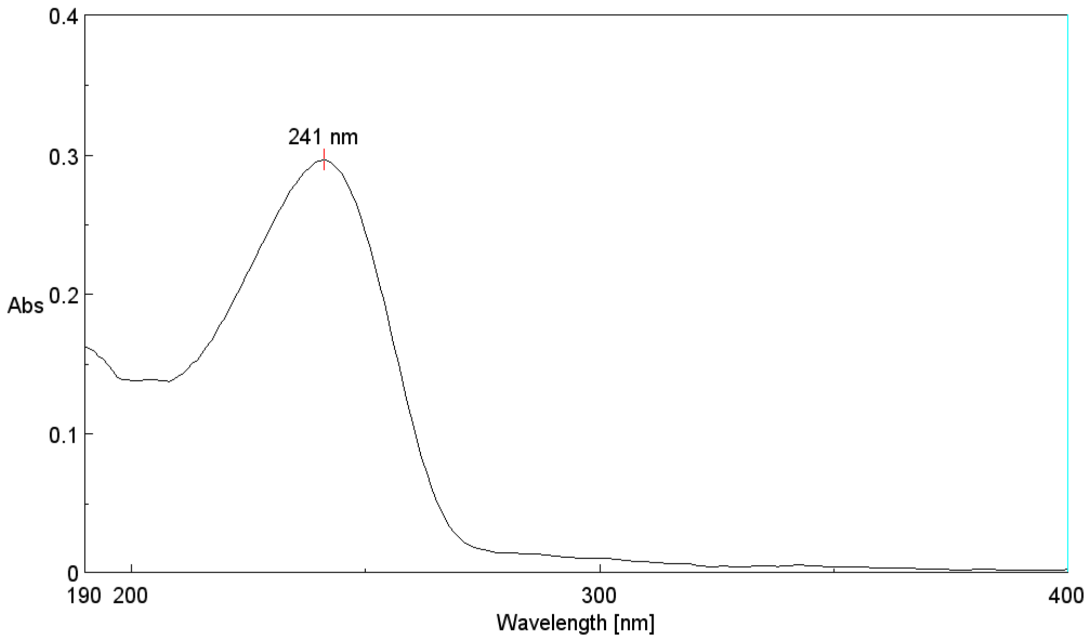

Figure S21. UV spectrum of compound **2**

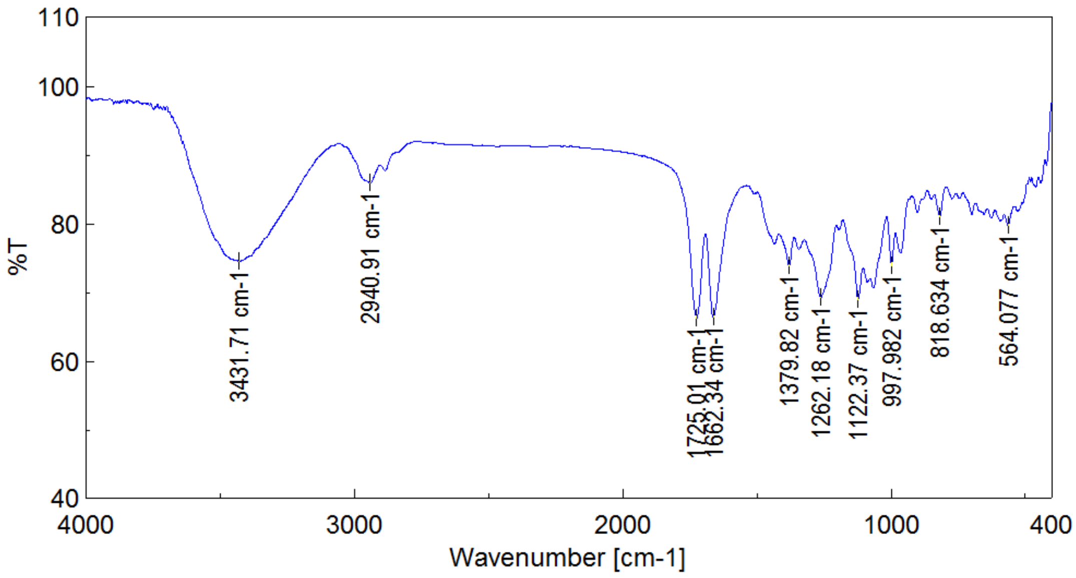

Figure S22. IR (KBr disc) spectrum of compound **2**

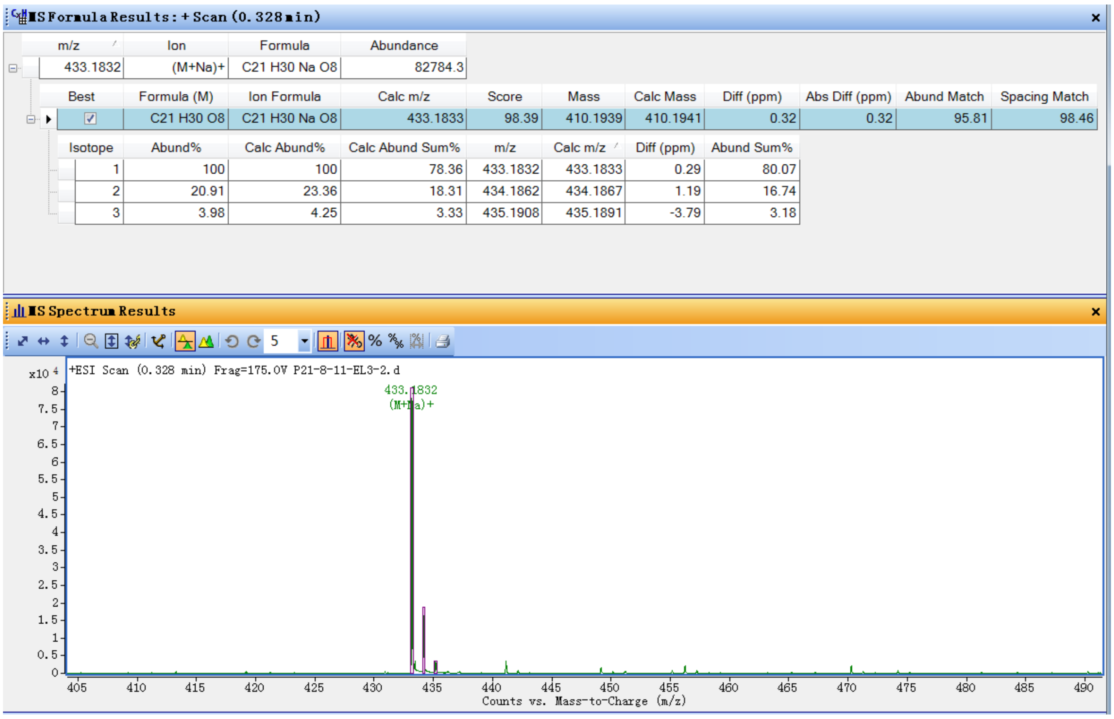

Figure S23. HR-ESI-MS spectrum of compound **2**

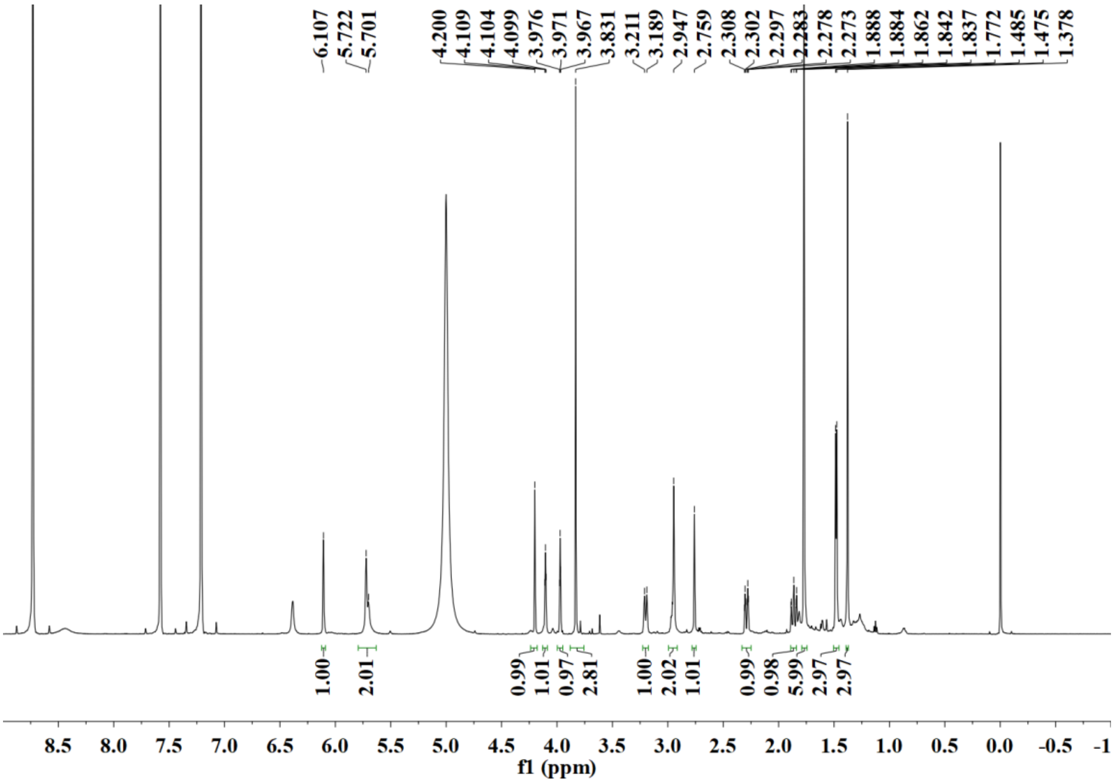

Figure S24. ¹H NMR spectrum of compound **2**

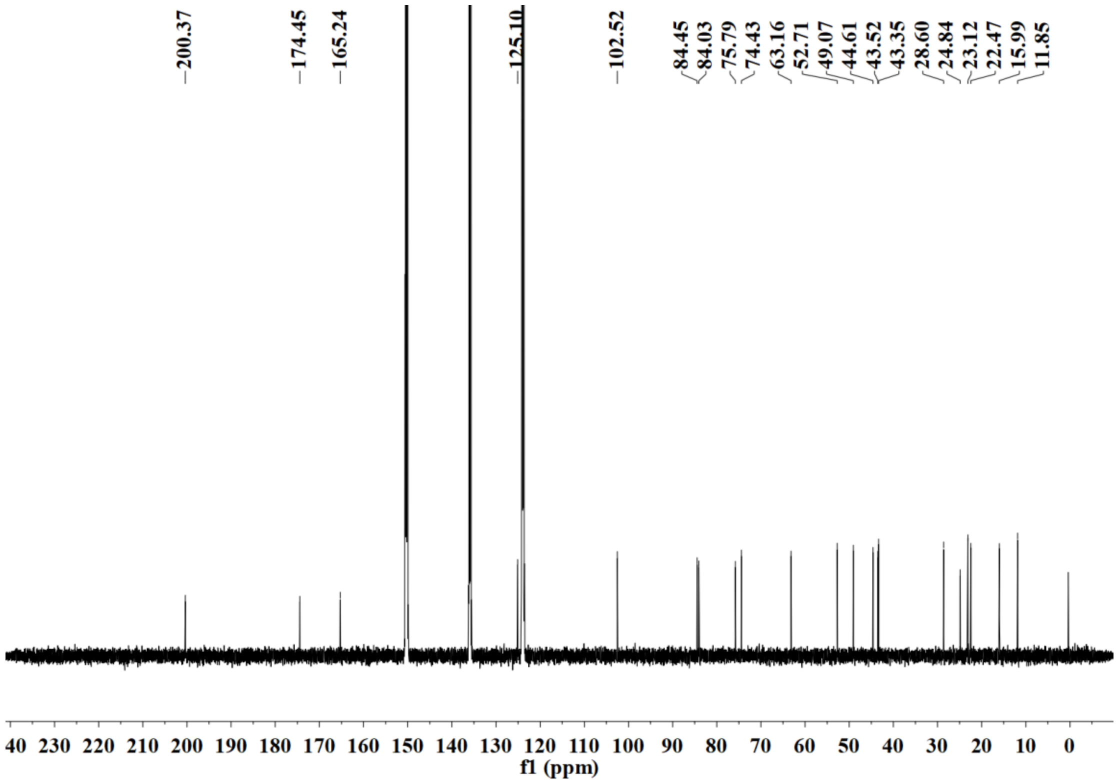

Figure S25. ^{13}C NMR spectrum of compound **2**

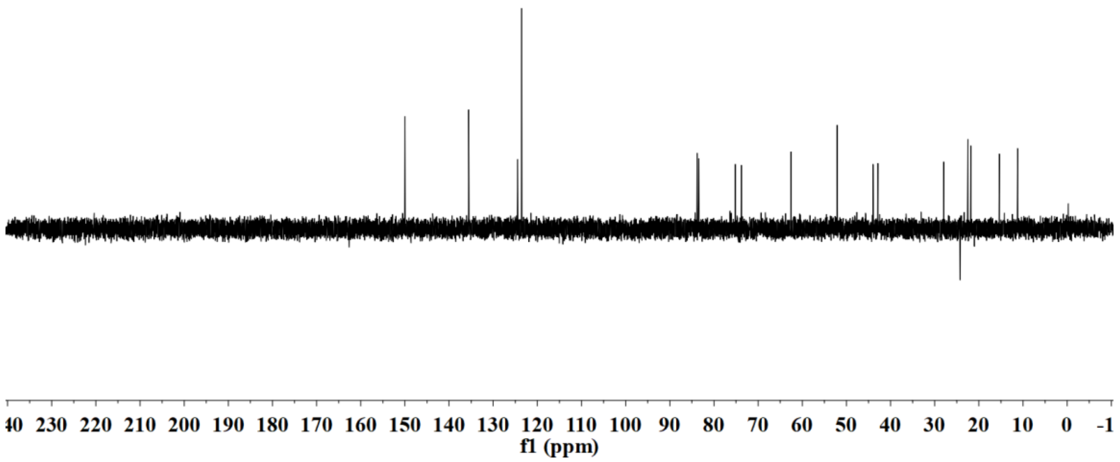

Figure S26. DEPT-135 spectrum of compound **2**

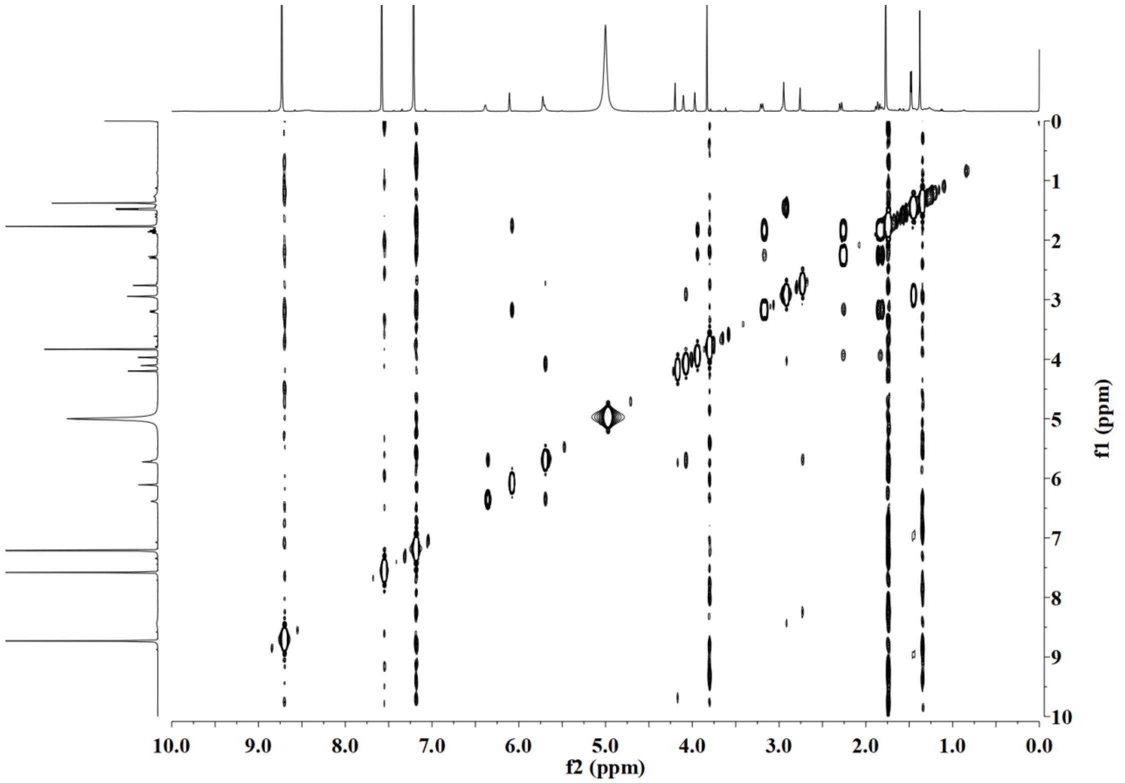

Figure S27. ^{1}H-^{1}H COSY spectrum of compound **2**

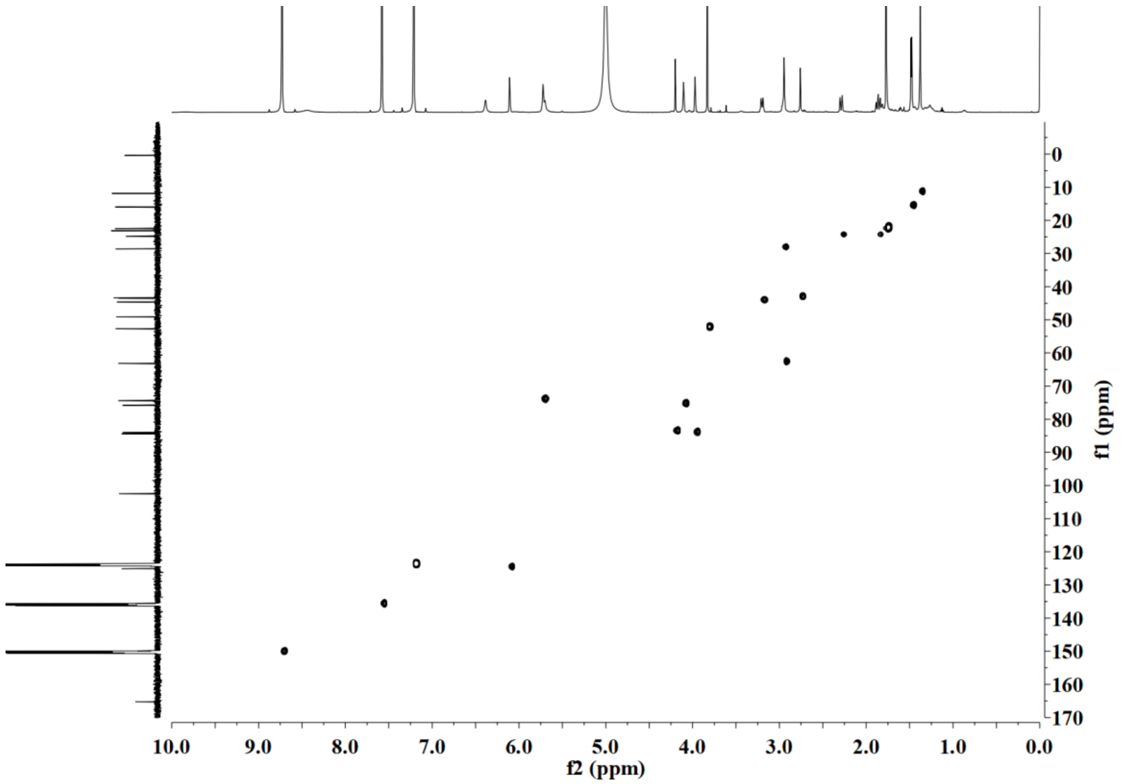

Figure S28. HSQC spectrum of compound **2**

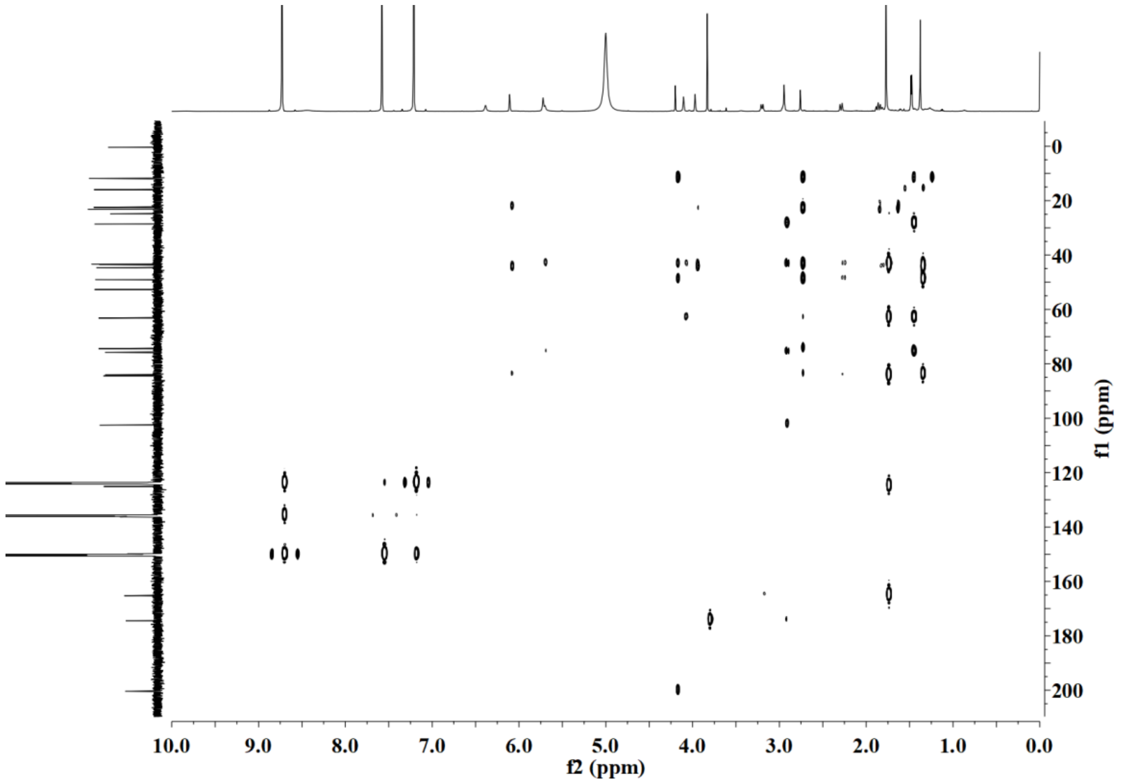

Figure S29. HMBC spectrum of compound **2**

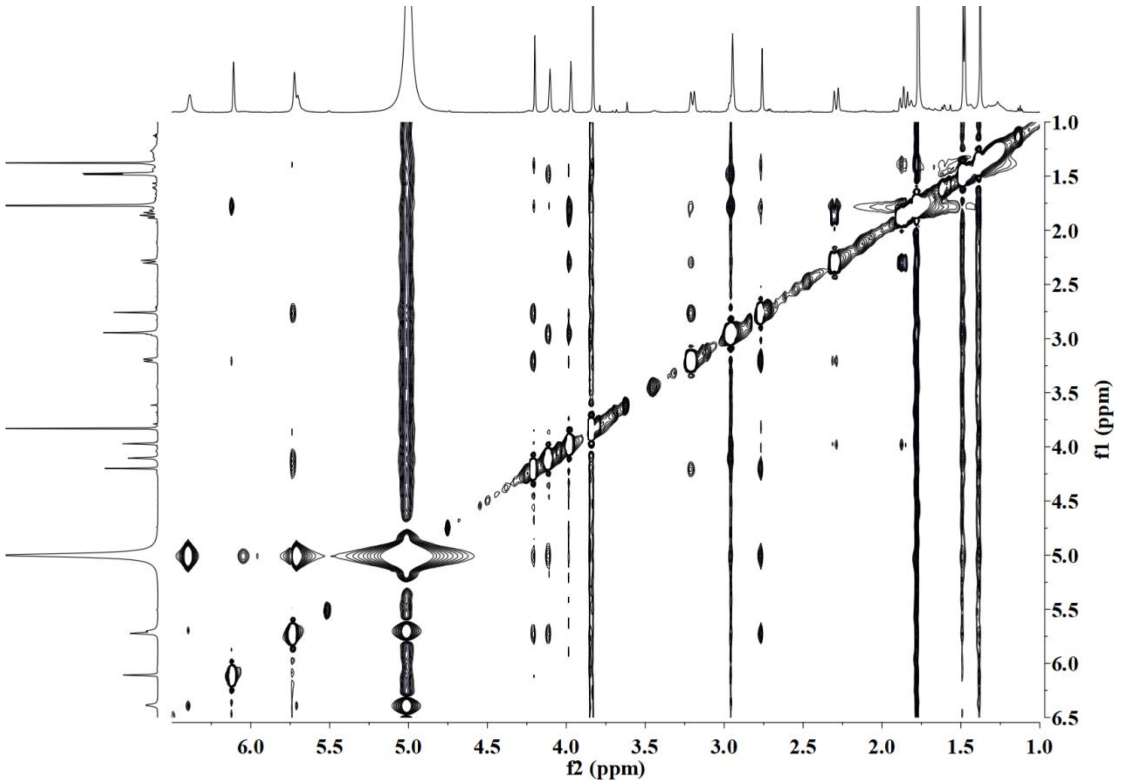

Figure S30. NOESY spectrum of compound **2**

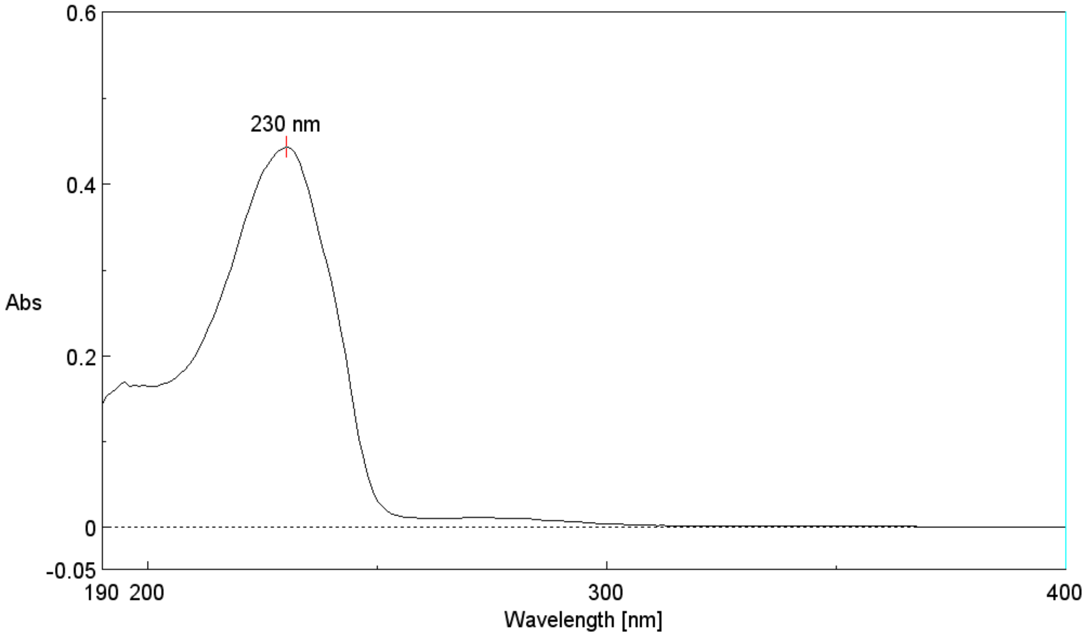

Figure S31. UV spectrum of compound **3**

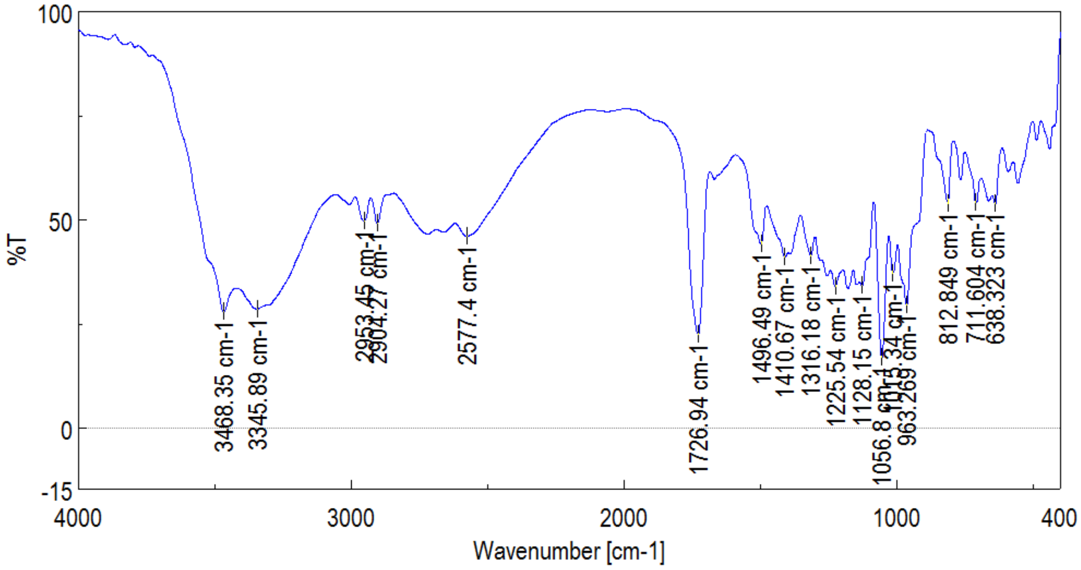

Figure S32. IR (KBr disc) spectrum of compound **3**

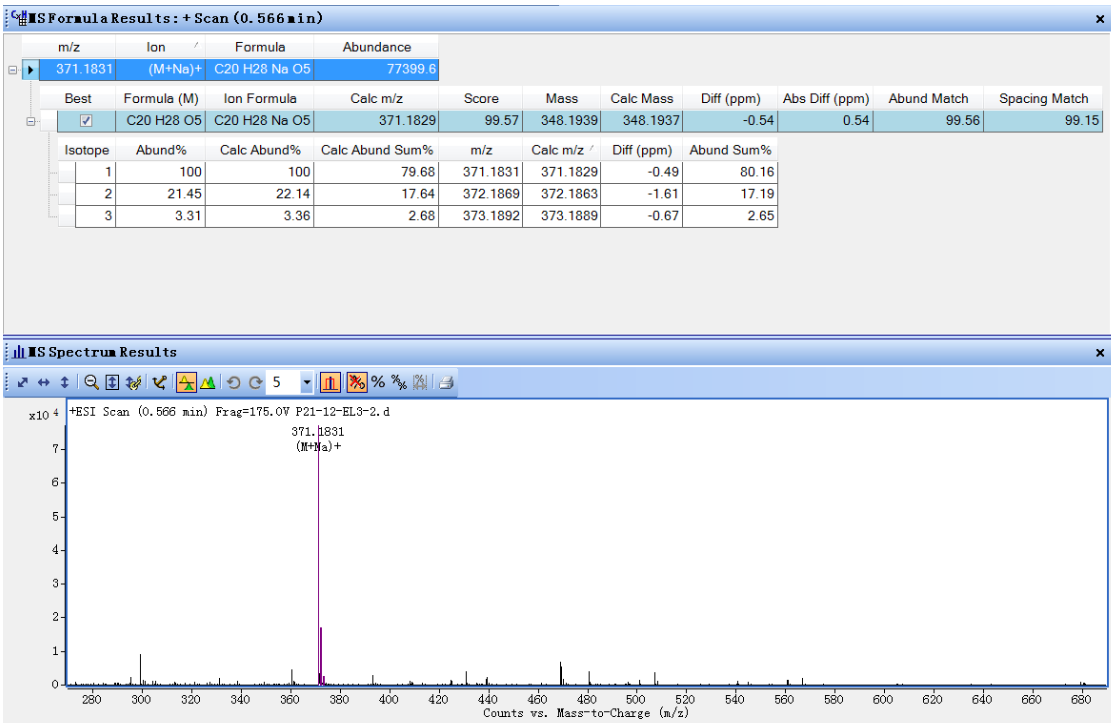

Figure S33. HR-ESI-MS spectrum of compound **3**

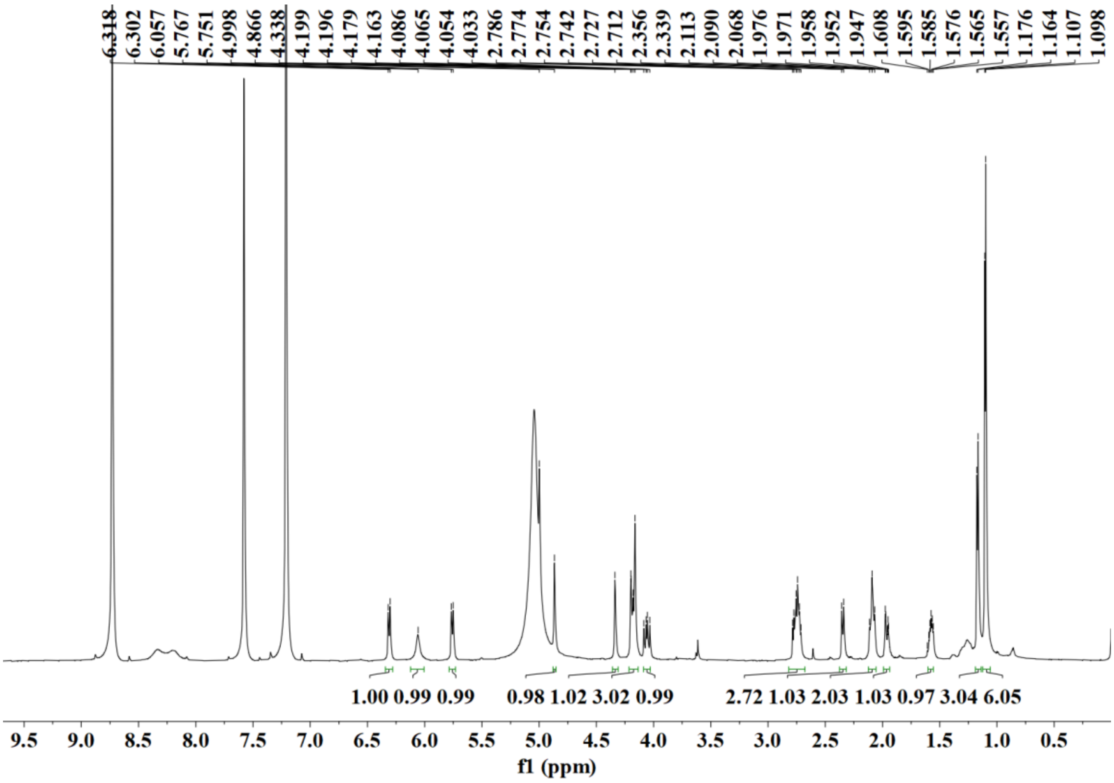

Figure S34. ^{1}H NMR spectrum of compound **3**

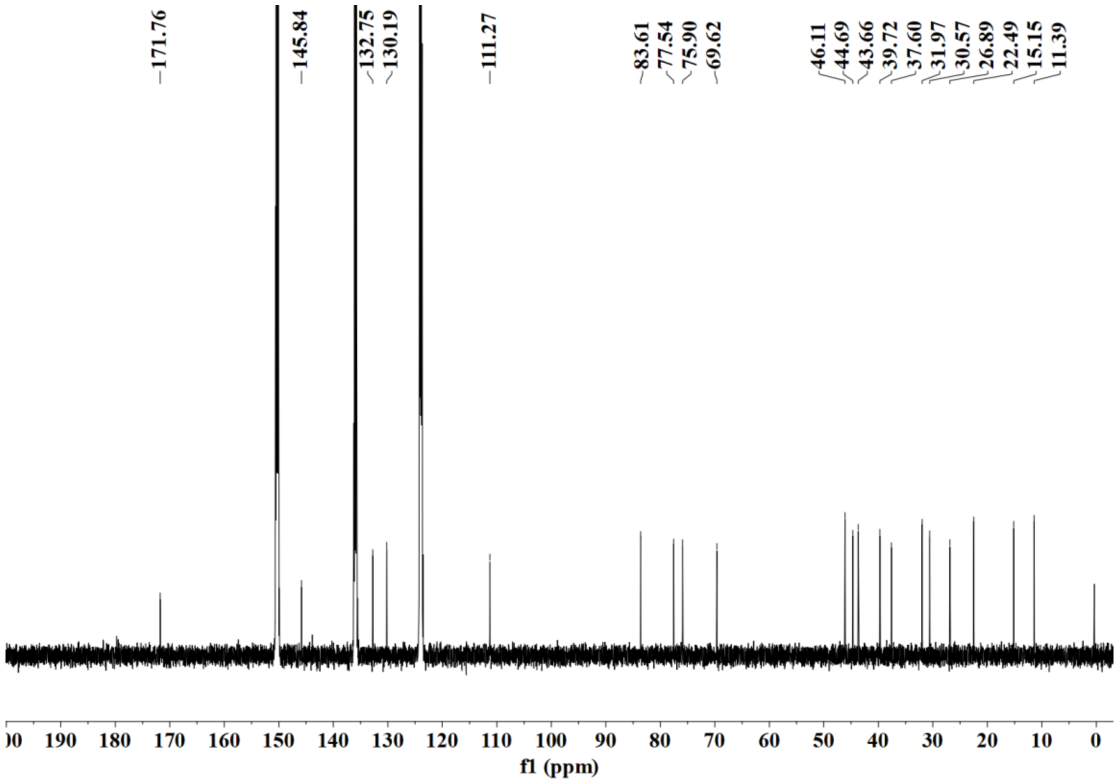

Figure S35. ^{13}C NMR spectrum of compound **3**

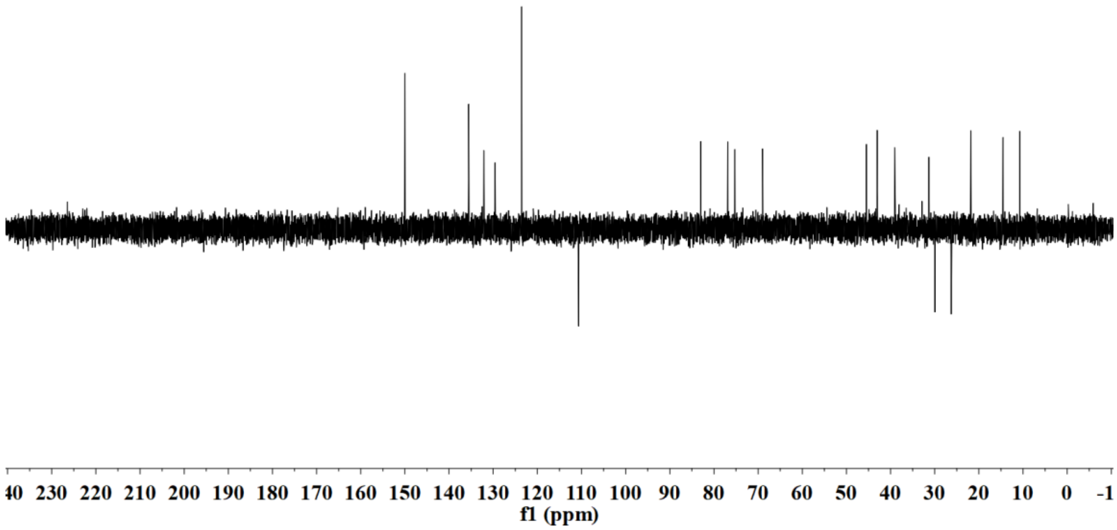

Figure S36. DEPT-135 spectrum of compound **3**

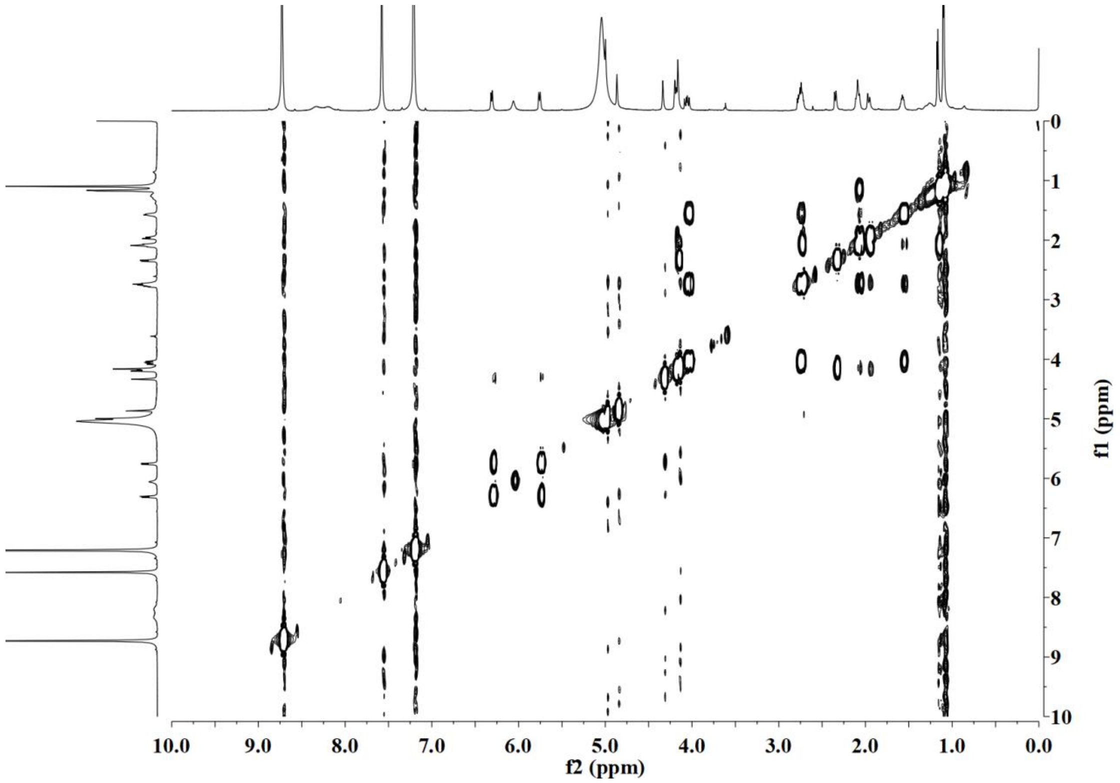

Figure S37. ^{1}H-^{1}H COSY spectrum of compound **3**

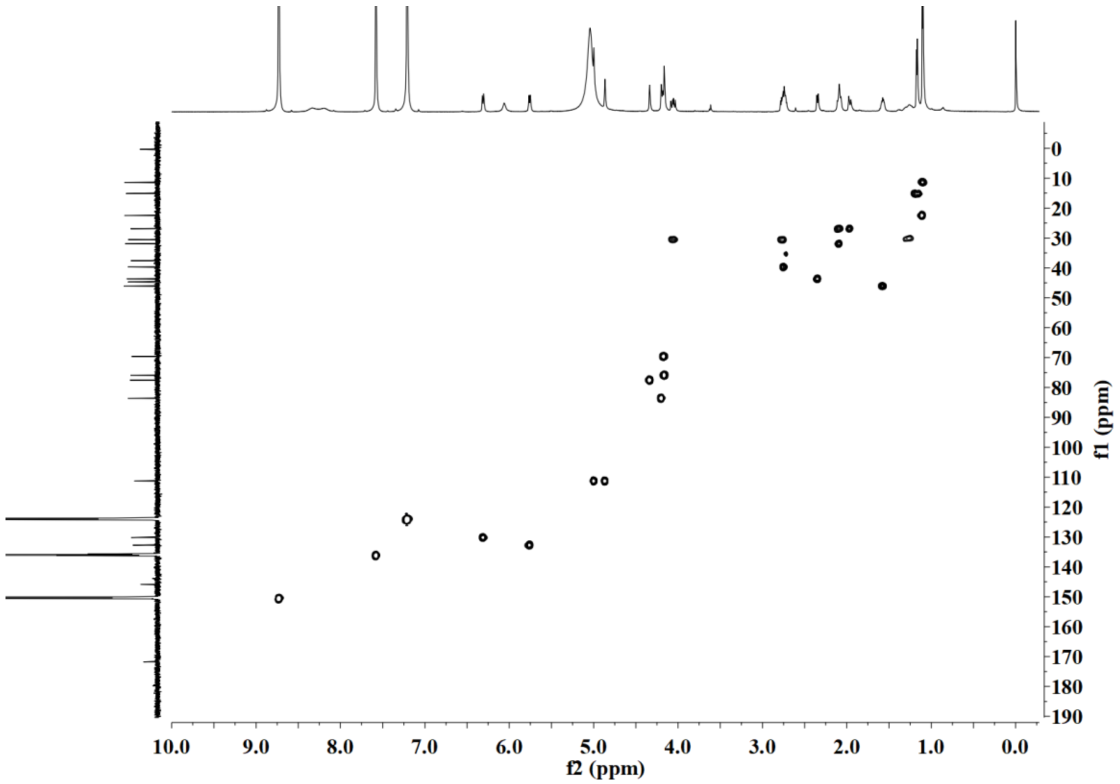

Figure S38. HSQC spectrum of compound **3**

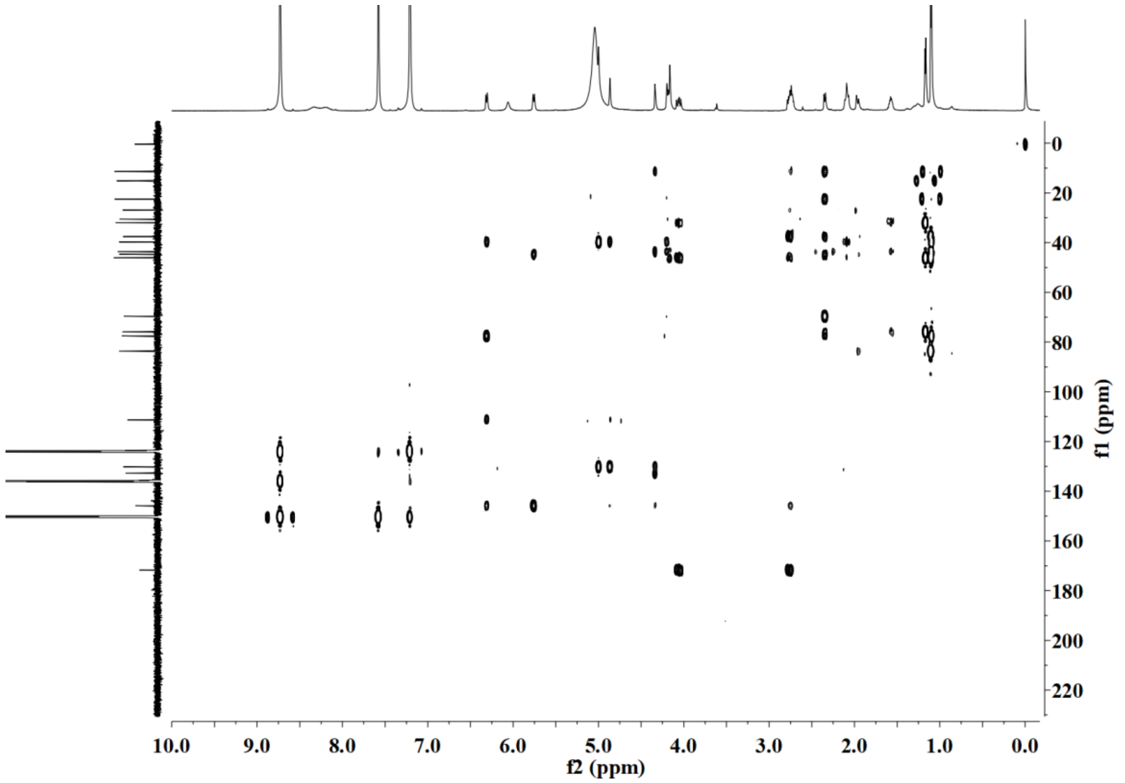

Figure S39. HMBC spectrum of compound **3**

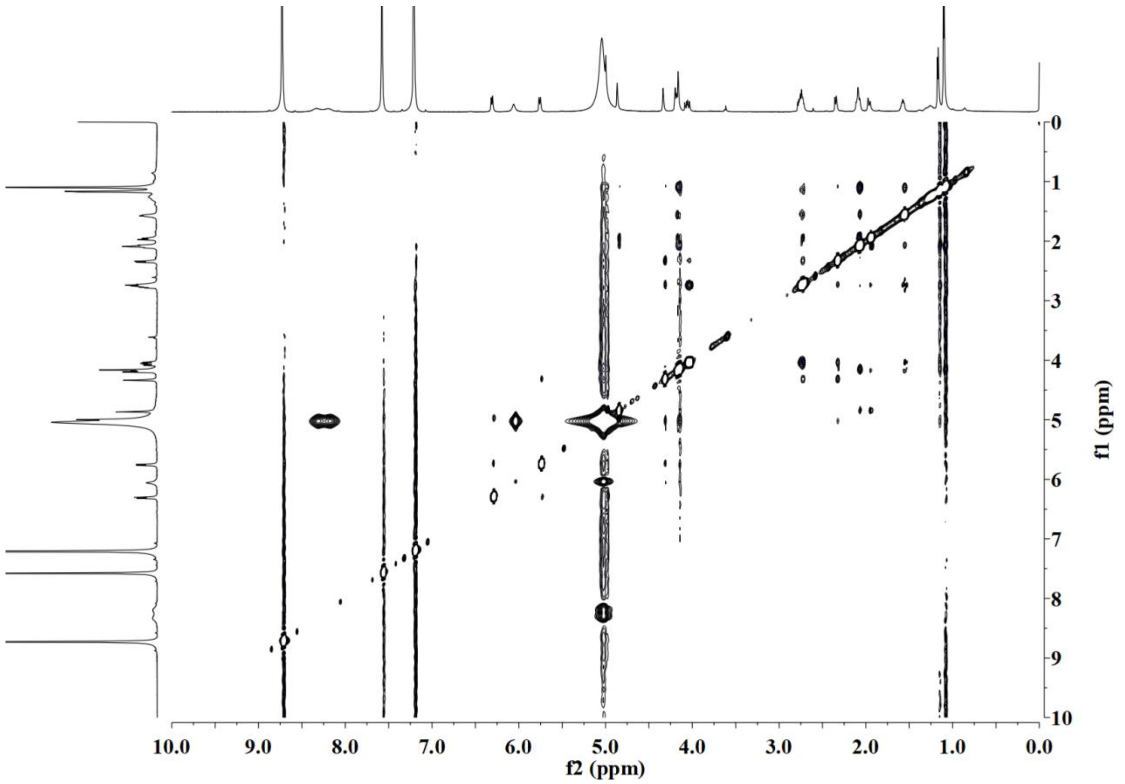

Figure S40. NOESY spectrum of compound **3**

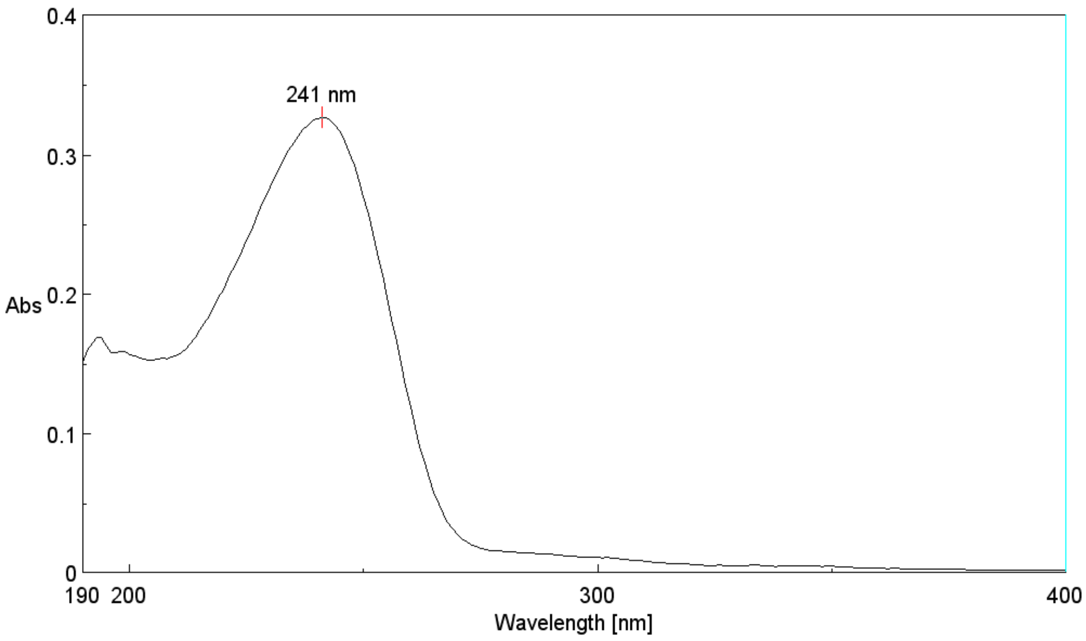

Figure S41. UV spectrum of compound **4**

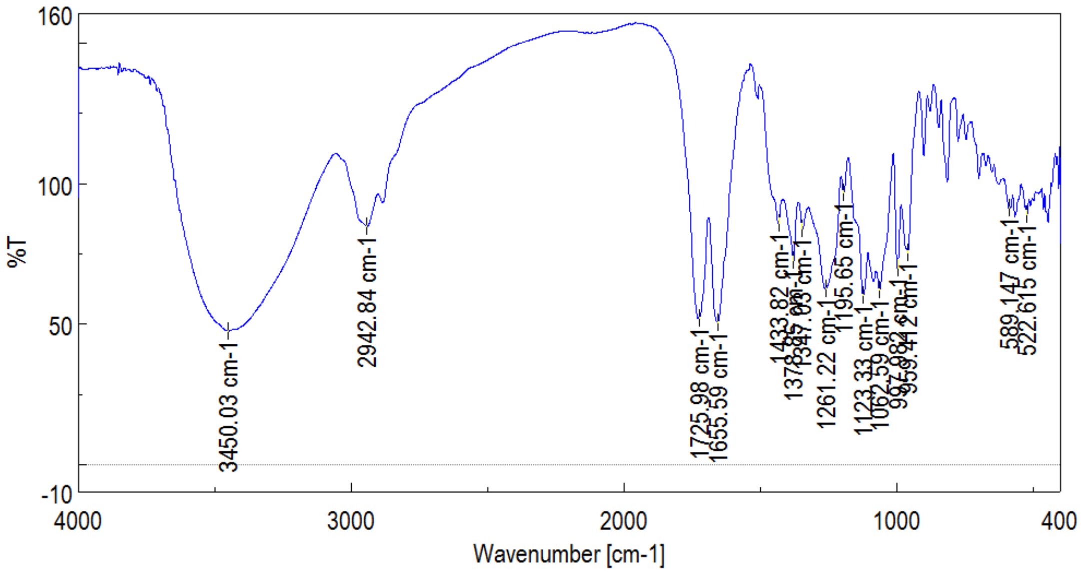

Figure S42. IR (KBr disc) spectrum of compound **4**

MS Formula Results: + Scan (0.782min)

m/z	Ion	Formula	Abundance
401.1574	(M+Na)+	C20 H26 Na O7	52616.4

Best	Formula (M)	Ion Formula	Calc m/z	Score	Mass	Calc Mass	Diff (ppm)	Abs Diff (ppm)	Abund Match	Spacing Match
☑	C20 H26 O7	C20 H26 Na O7	401.1571	90.71	378.1682	378.1679	-0.95	0.95	96.24	66.94

Isotope	Abund%	Calc Abund%	Calc Abund Sum%	m/z	Calc m/z	Diff (ppm)	Abund Sum%
1	100	100	79.38	401.1574	401.1571	-0.88	78.69
2	21.61	22.2	17.62	402.1616	402.1605	-2.88	17.01
3	5.47	3.79	3	403.1536	403.1629	23.16	4.3

MS Spectrum Results

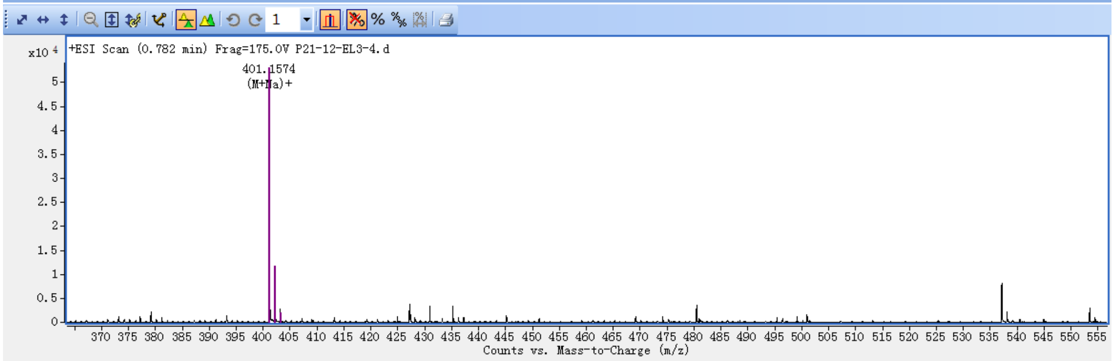

Figure S43. HR-ESI-MS spectrum of compound **4**

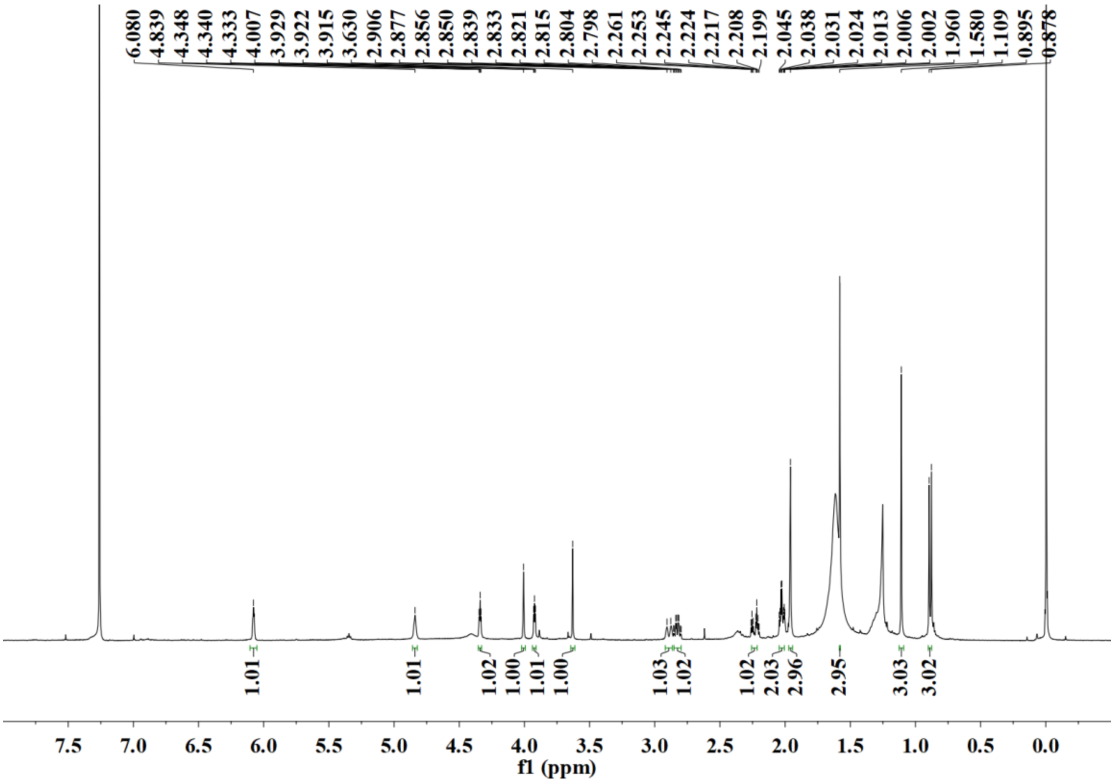

Figure S44. ^{1}H NMR spectrum of compound **4**

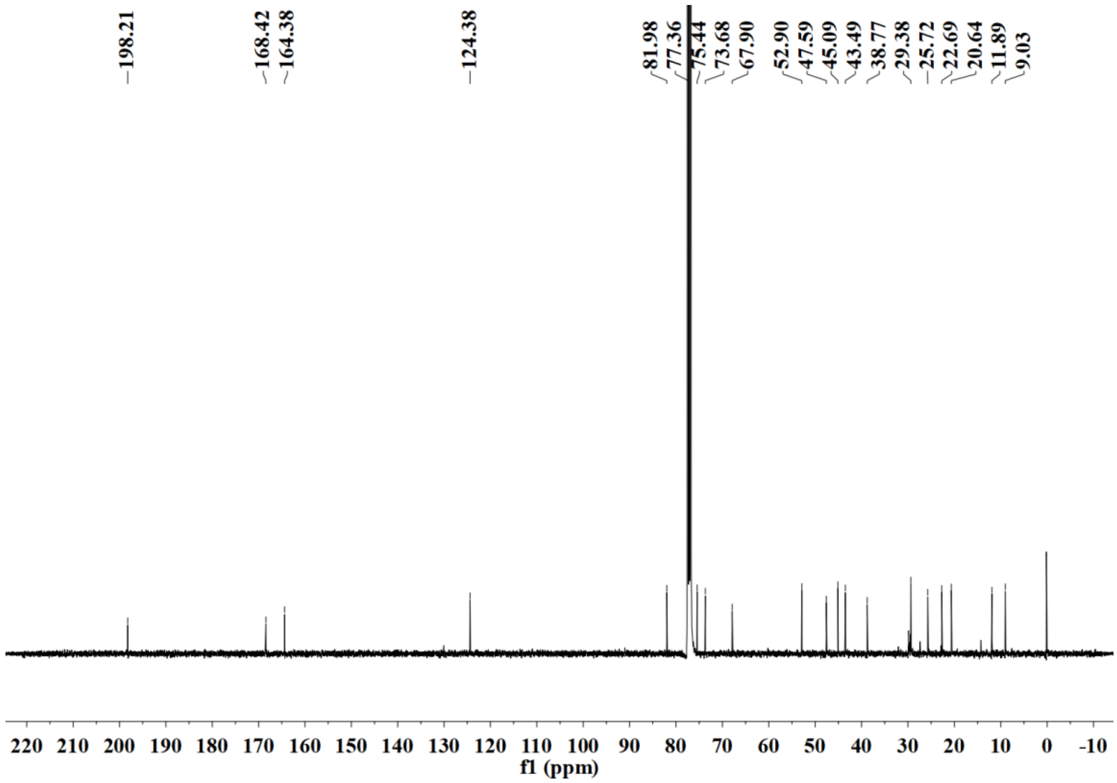

Figure S45. ^{13}C NMR spectrum of compound **4**

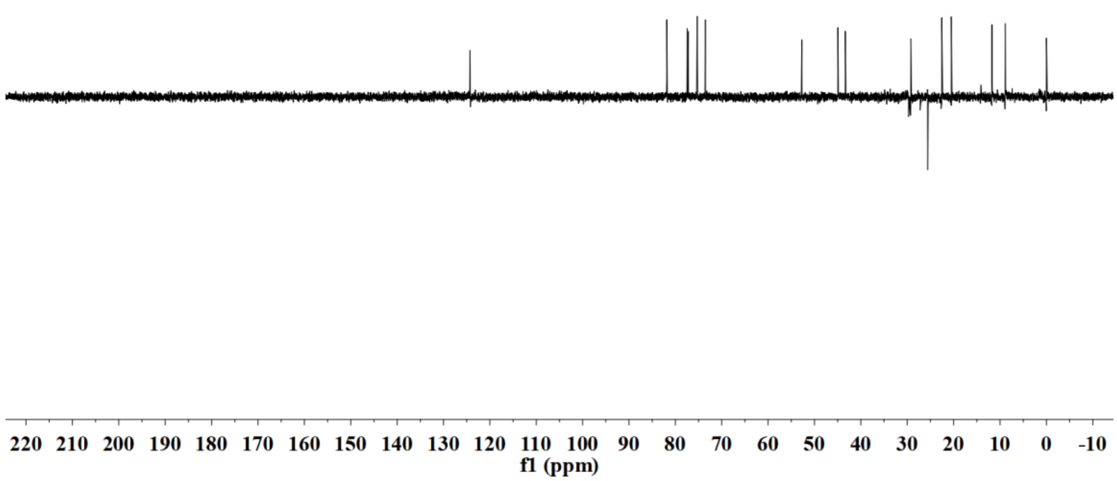

Figure S46. DEPT-135 spectrum of compound **4**

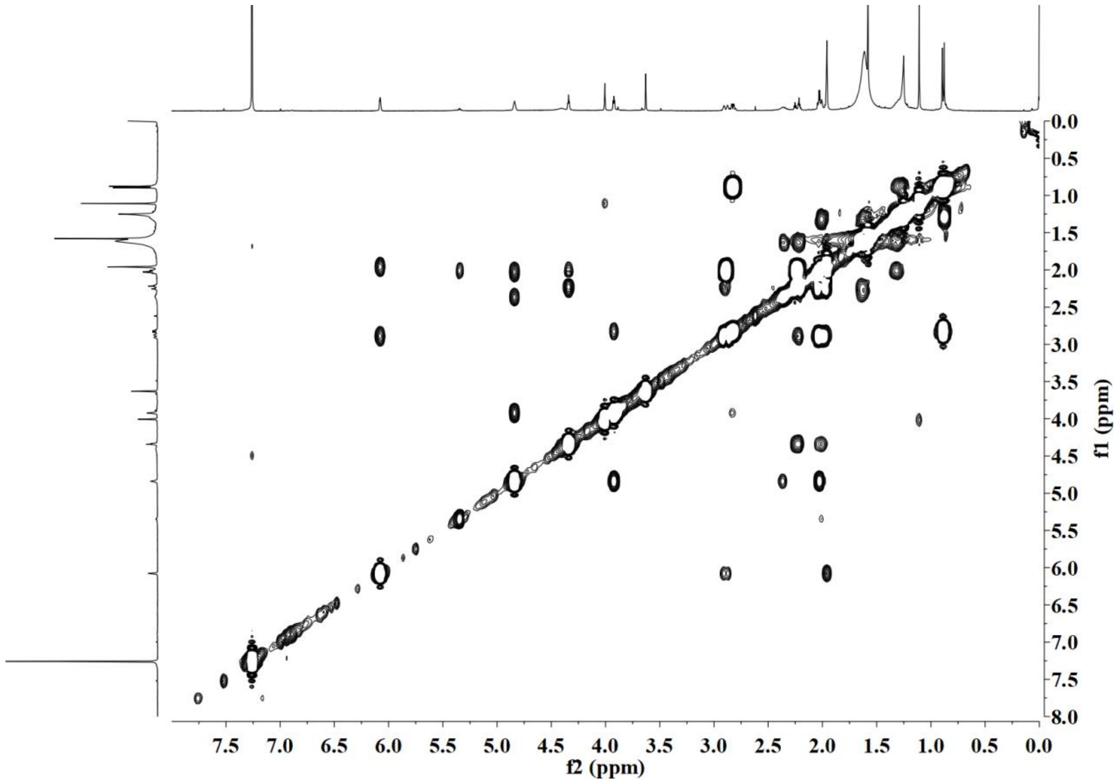

Figure S47. ^{1}H-^{1}H COSY spectrum of compound **4**

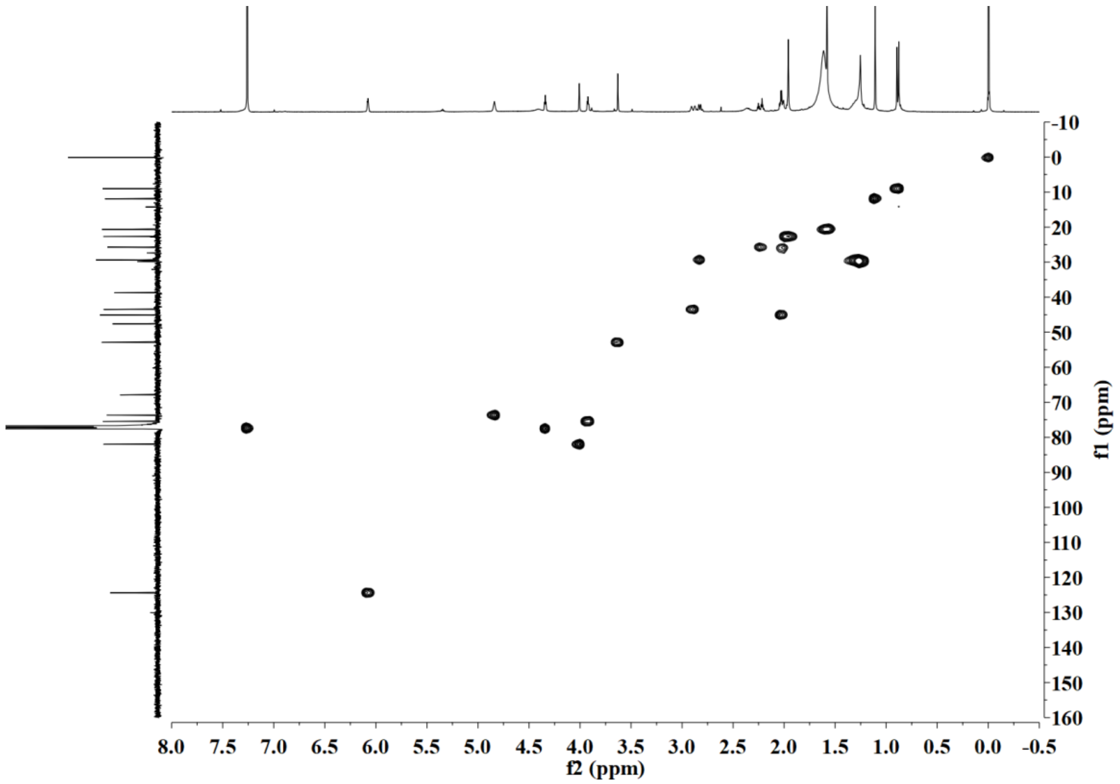

Figure S48. HSQC spectrum of compound **4**

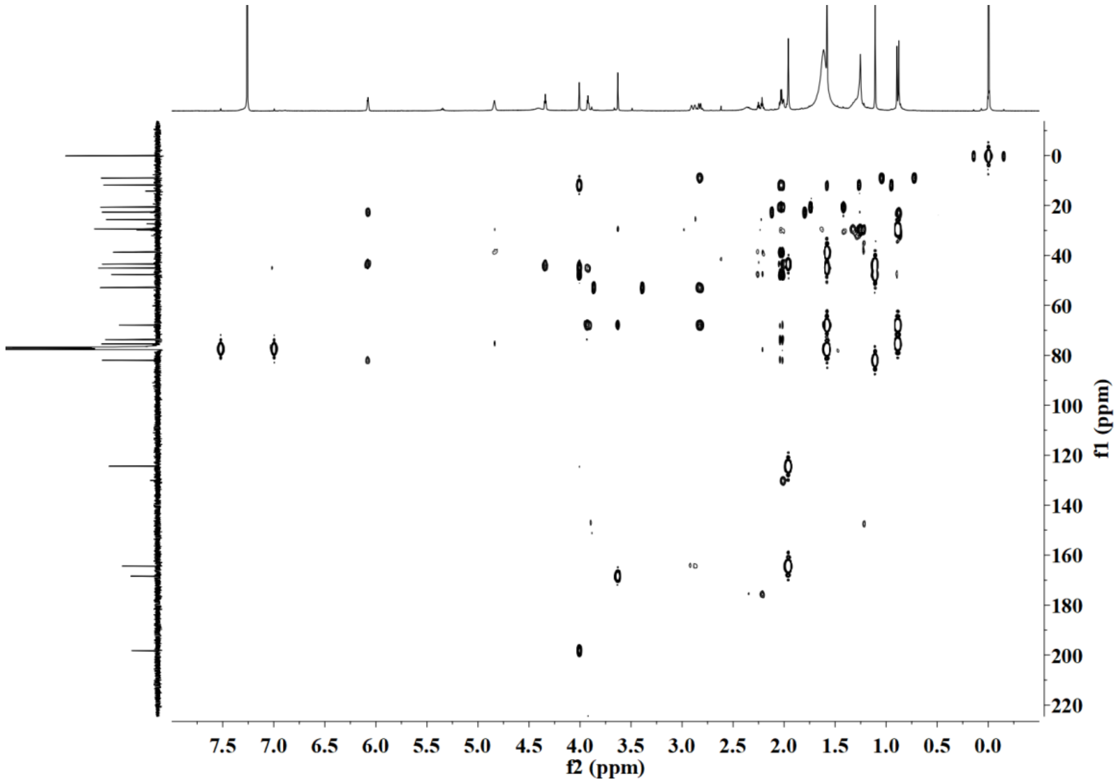

Figure S49. HMBC spectrum of compound **4**

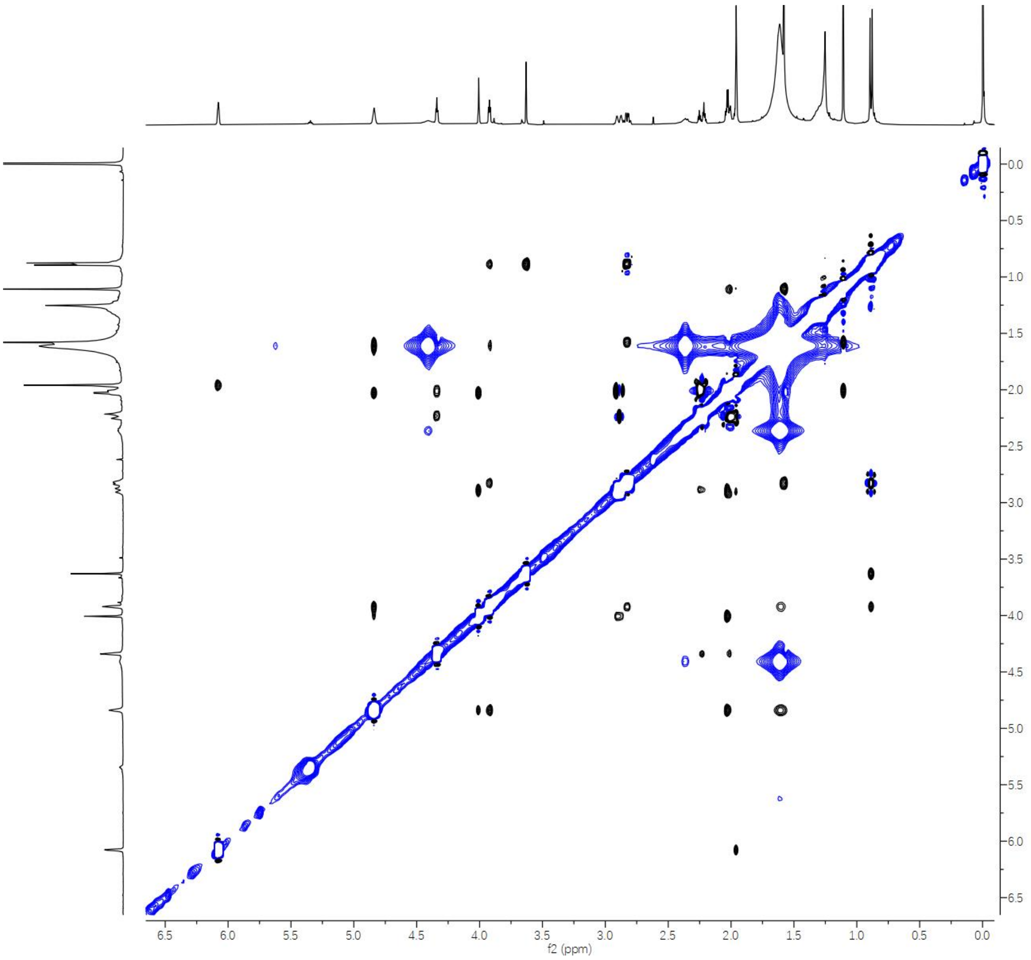

Figure S50. NOESY spectrum of compound **4**

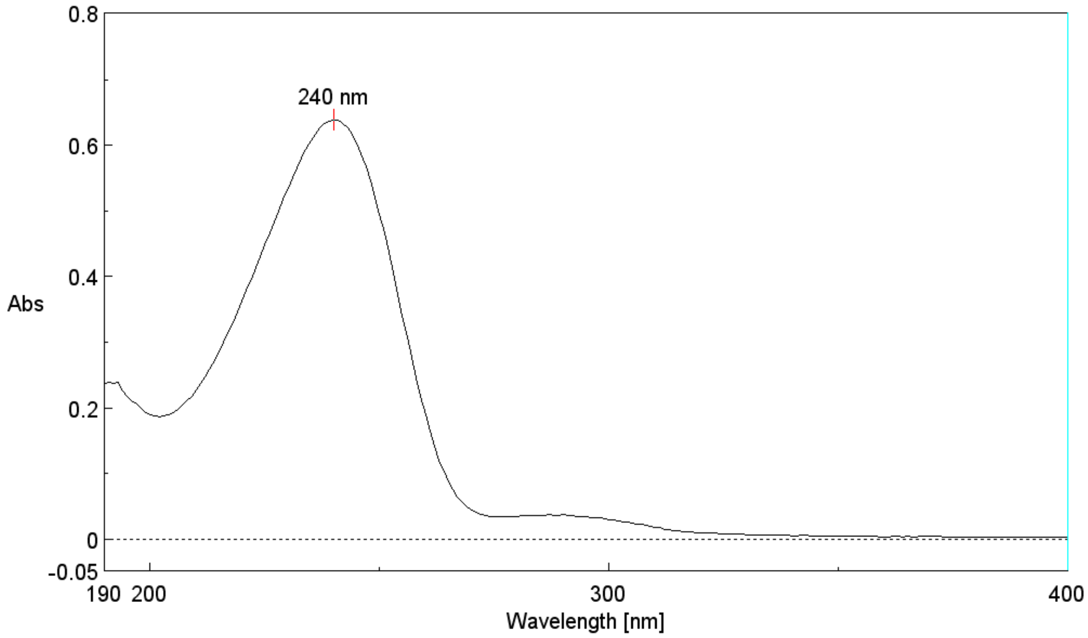

Figure S51. UV spectrum of compound **5**

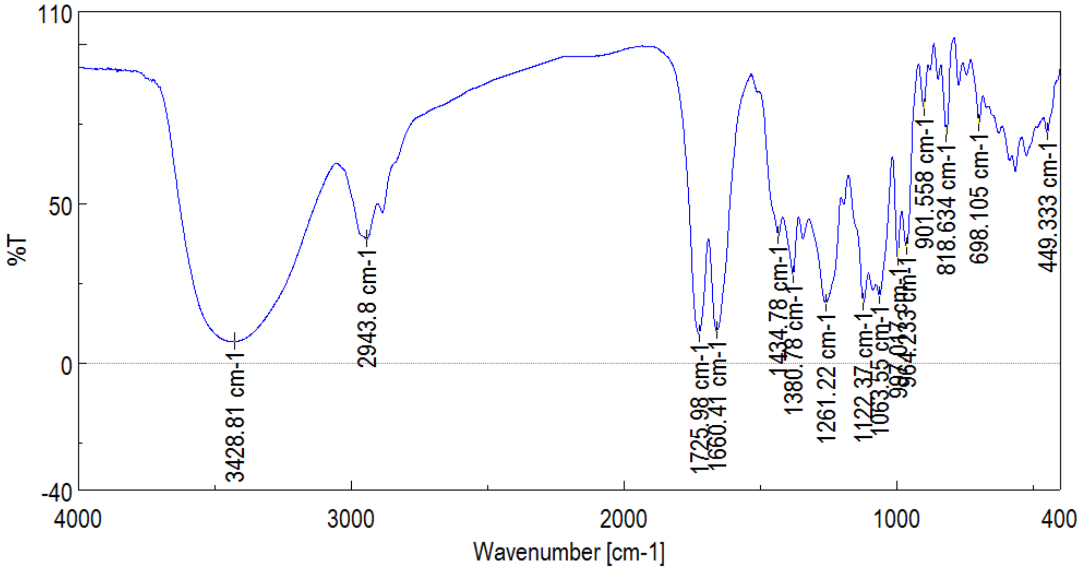

Figure S52. IR (KBr disc) spectrum of compound **5**

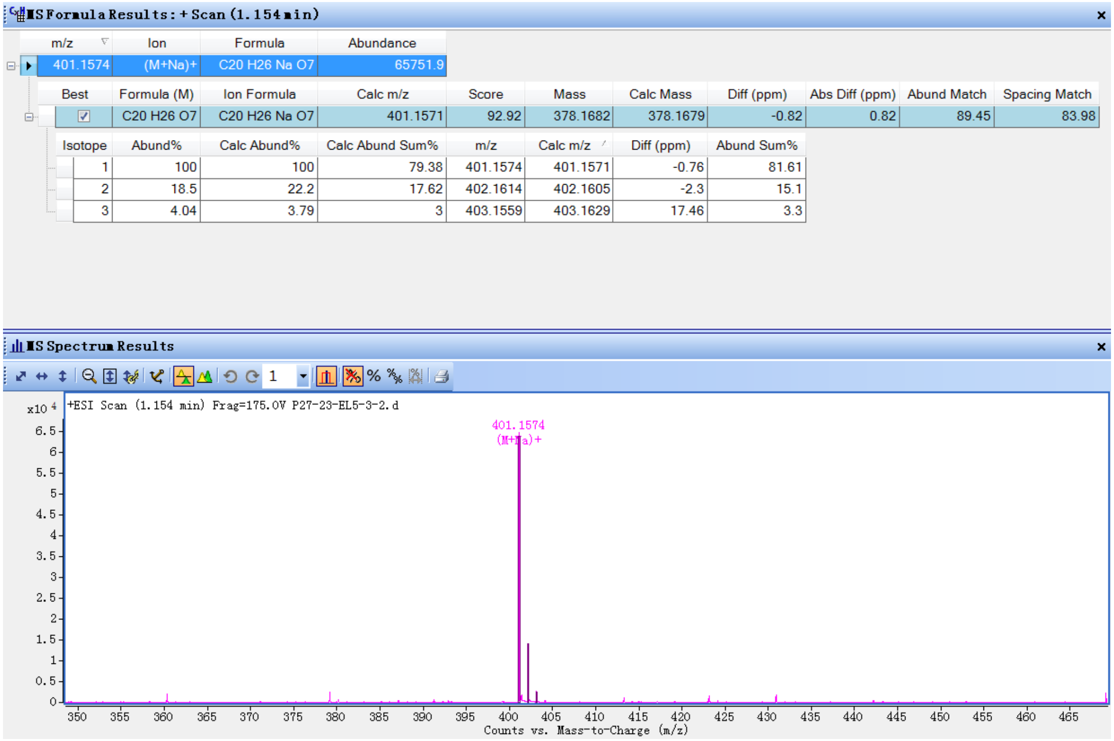

Figure S53. HR-ESI-MS spectrum of compound **5**

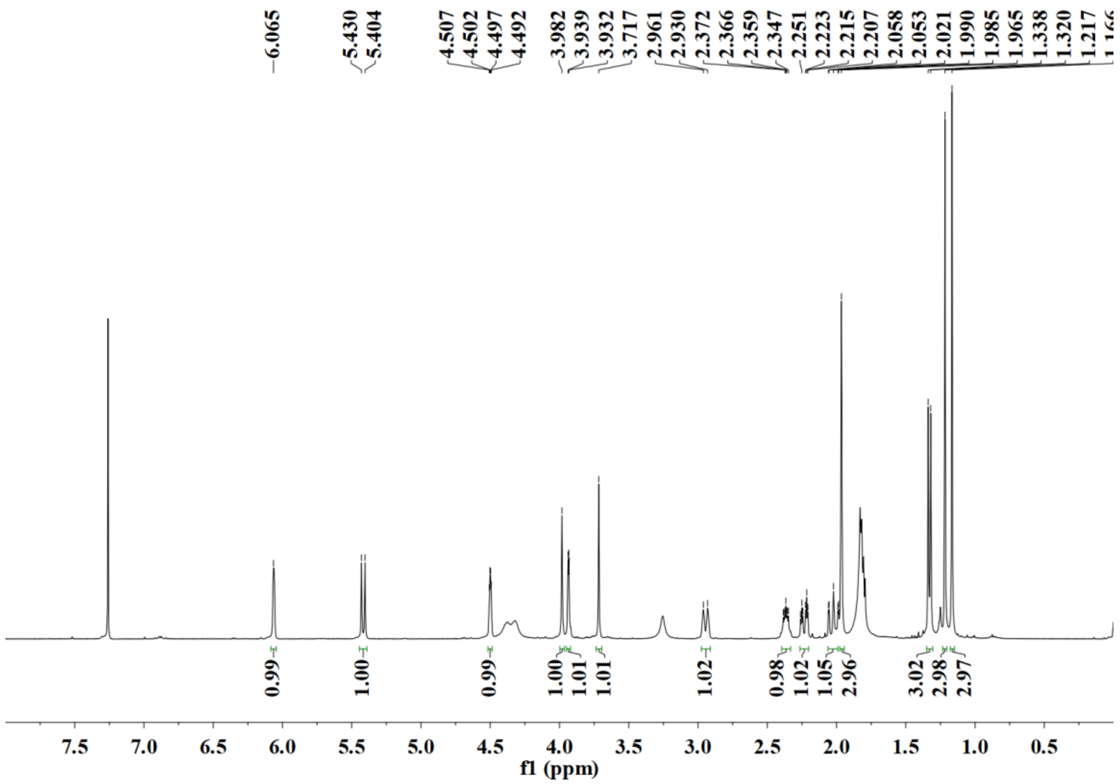

Figure S54. ^{1}H NMR spectrum of compound **5**

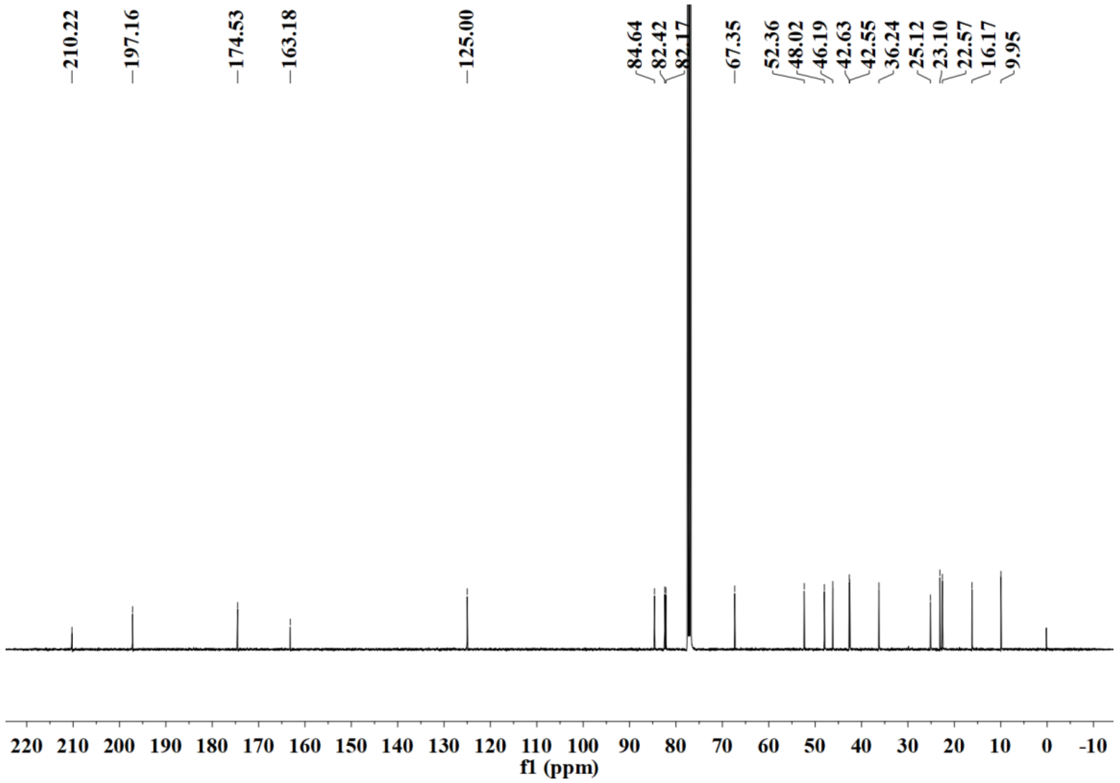

Figure S55. ^{13}C NMR spectrum of compound **5**

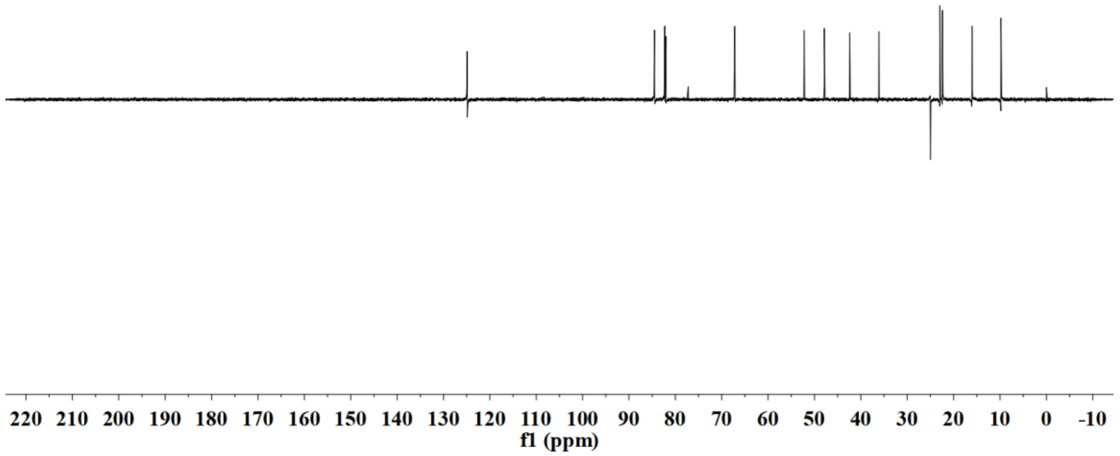

Figure S56. DEPT-135 spectrum of compound **5**

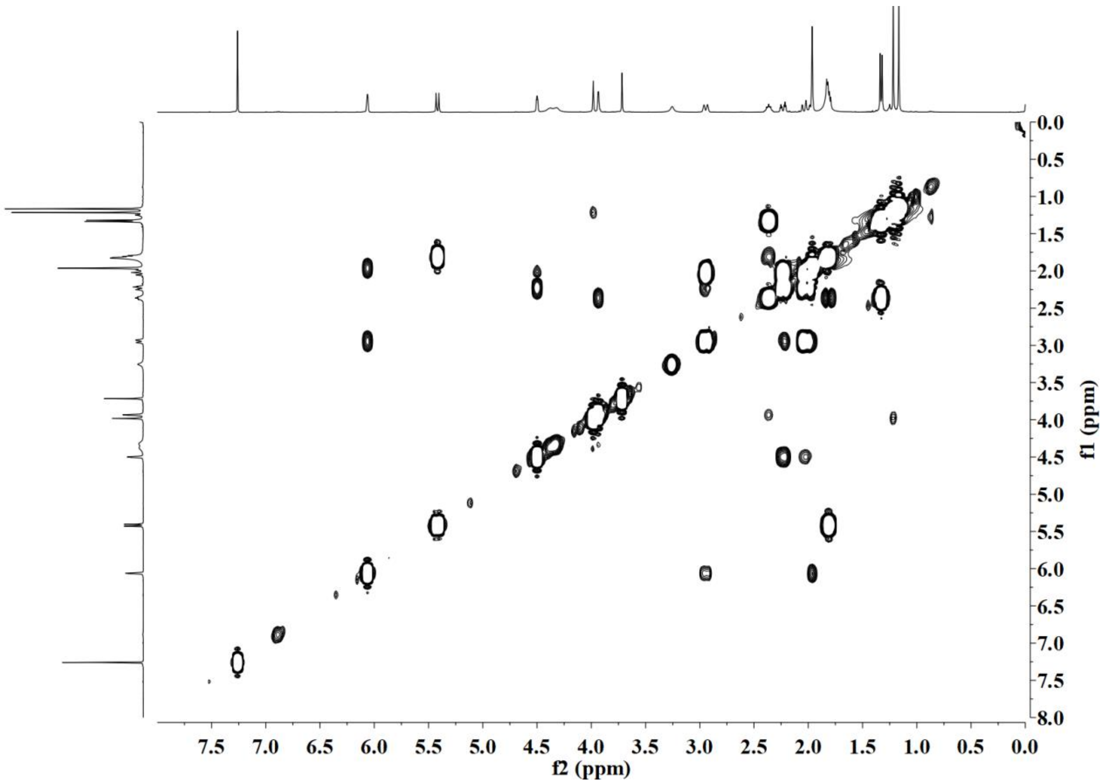

Figure S57. ^{1}H-^{1}H COSY spectrum of compound **5**

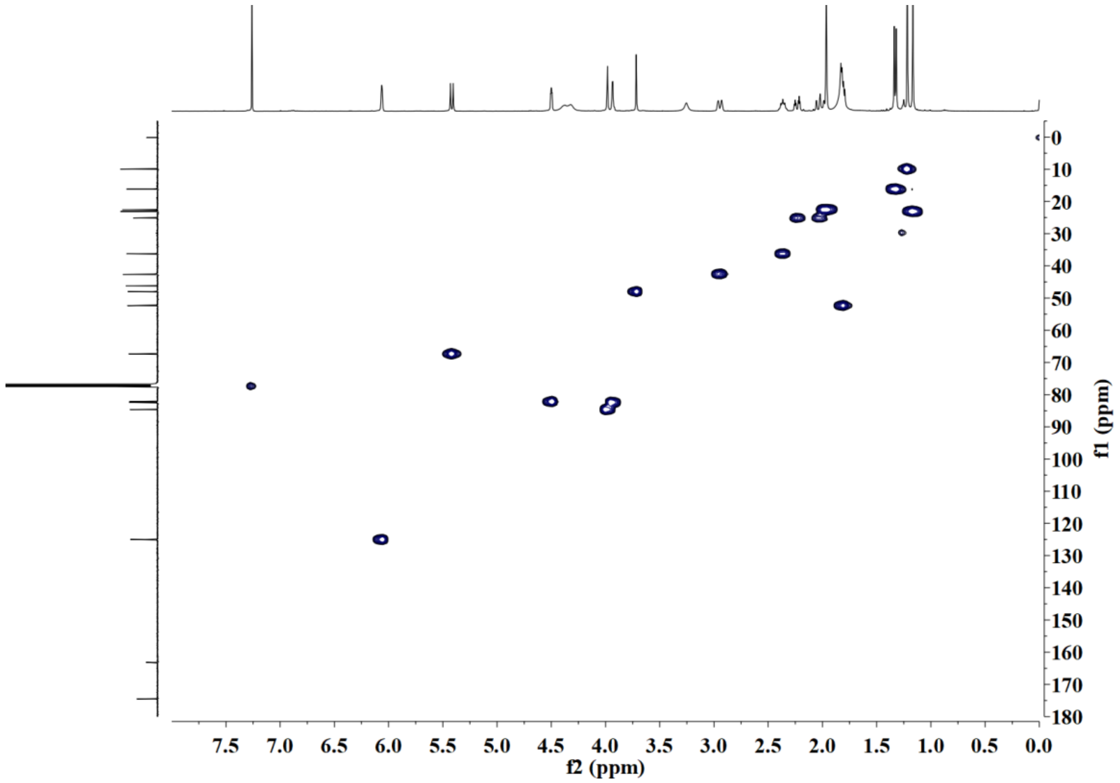

Figure S58. HSQC spectrum of compound **5**

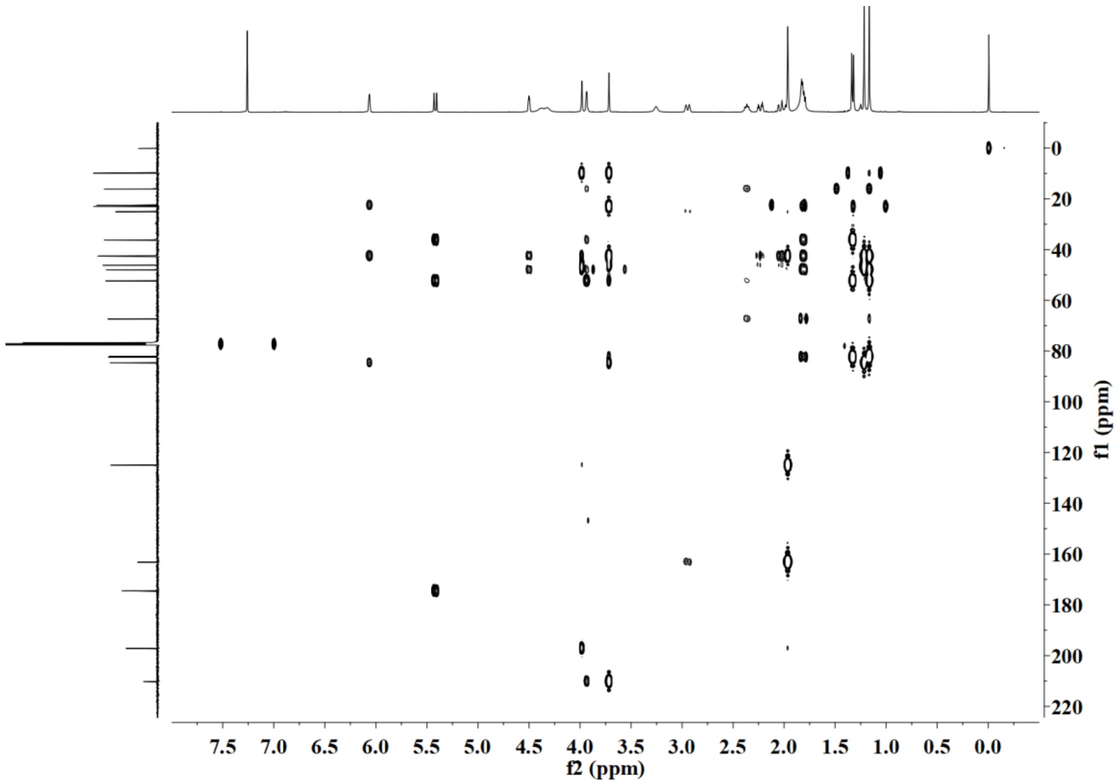

Figure S59. HMBC spectrum of compound **5**

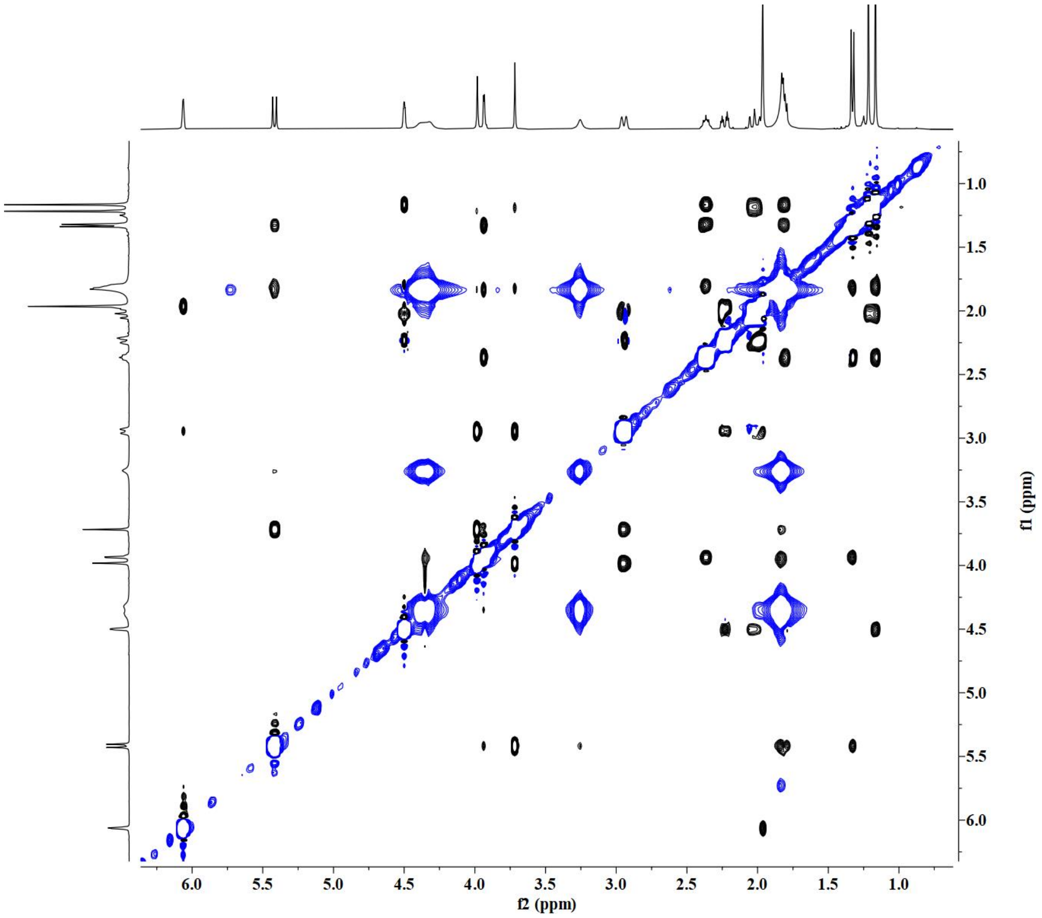

Figure S60. NOESY spectrum of compound **5**

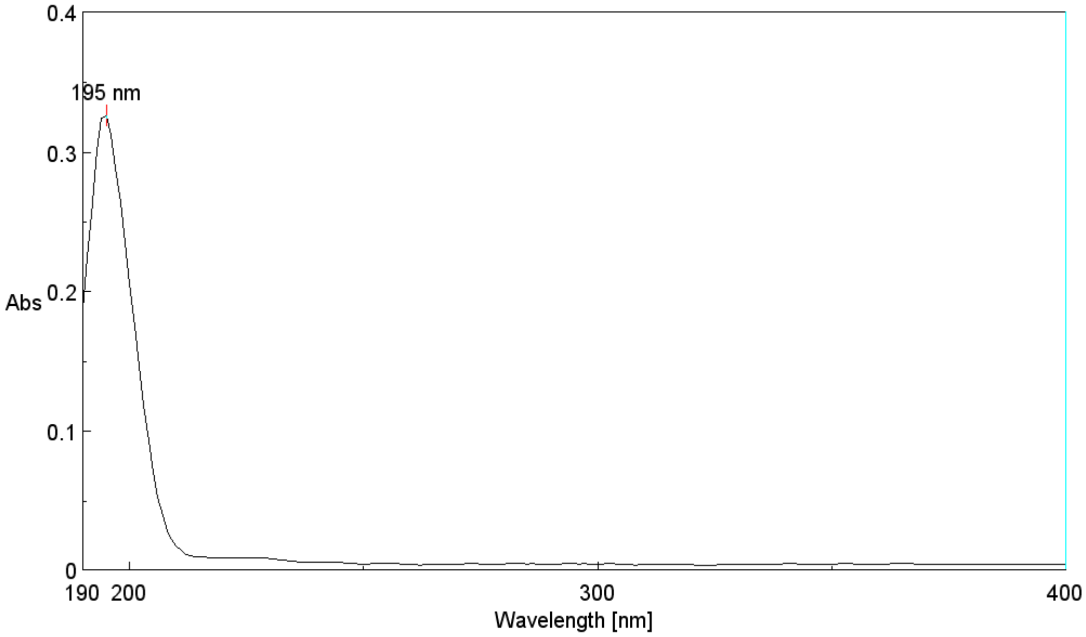

Figure S61. UV spectrum of compound **6**

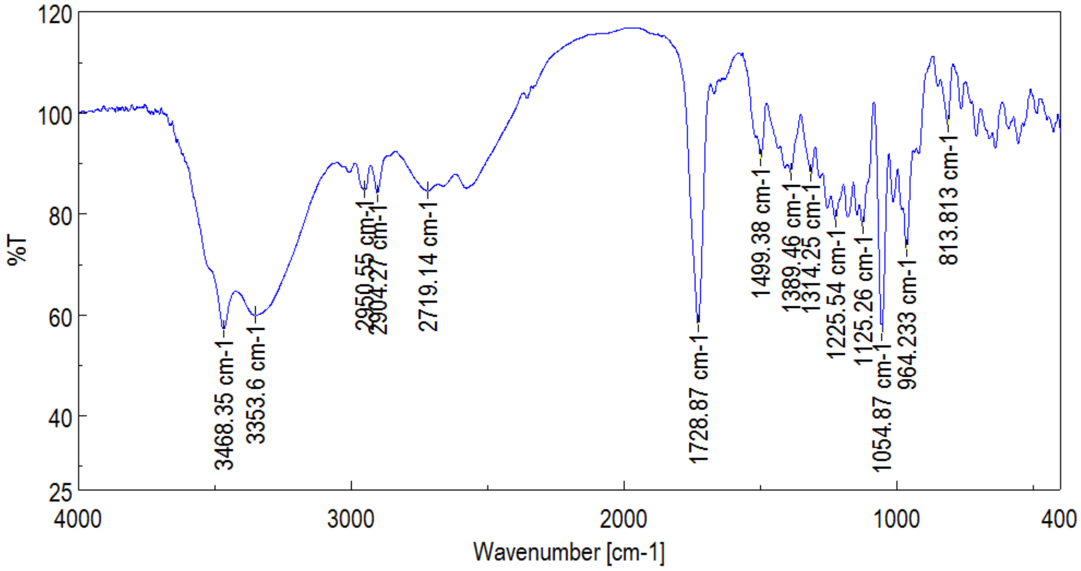

Figure S62. IR (KBr disc) spectrum of compound **6**

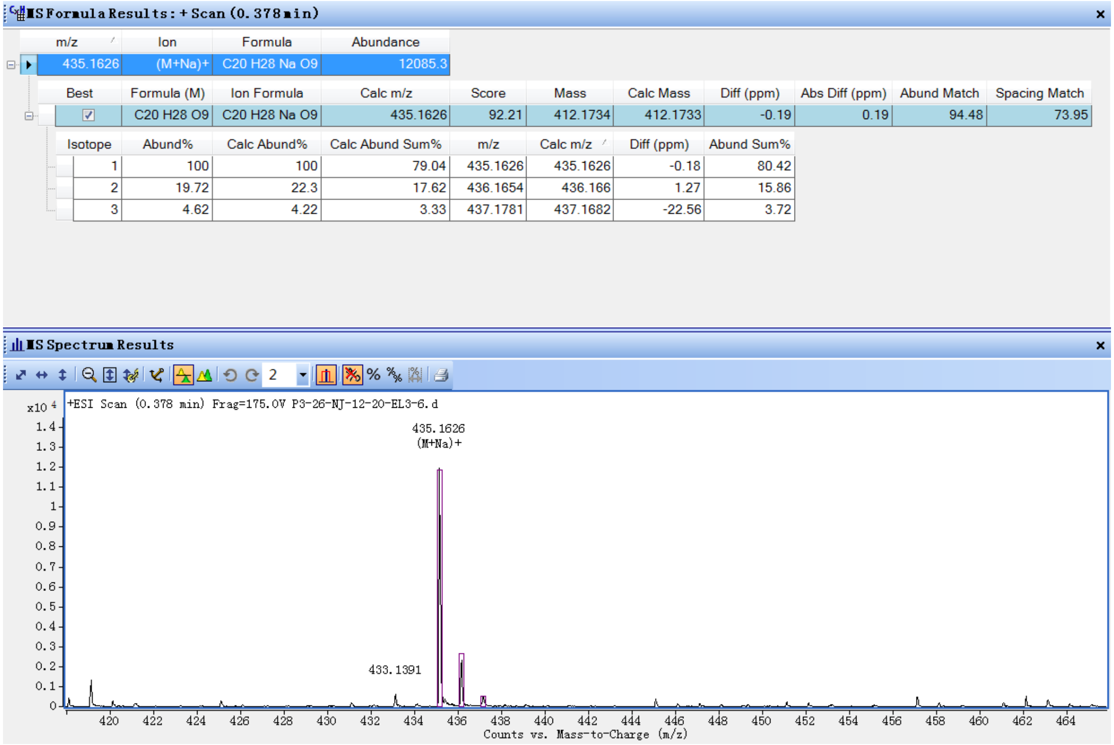

m/z	Ion	Formula	Abundance
435.1626	(M+Na)+	C20 H28 Na O9	12085.3

Best	Formula (M)	Ion Formula	Calc m/z	Score	Mass	Calc Mass	Diff (ppm)	Abs Diff (ppm)	Abund Match	Spacing Match
☑	C20 H28 O9	C20 H28 Na O9	435.1626	92.21	412.1734	412.1733	-0.19	0.19	94.48	73.95

Isotope	Abund%	Calc Abund%	Calc Abund Sum%	m/z	Calc m/z	Diff (ppm)	Abund Sum%
1	100	100	79.04	435.1626	435.1626	-0.18	80.42
2	19.72	22.3	17.62	436.1654	436.166	1.27	15.86
3	4.62	4.22	3.33	437.1781	437.1682	-22.56	3.72

Figure S63. HR-ESI-MS spectrum of compound **6**

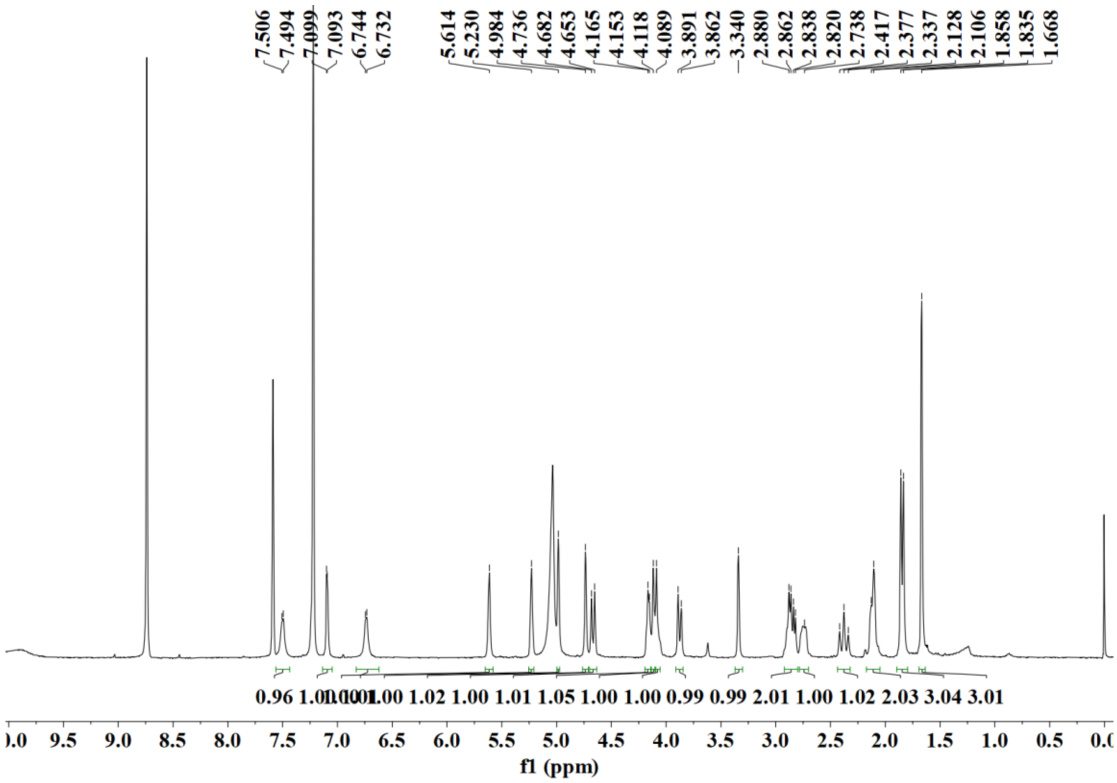

Figure S64. ¹H NMR spectrum of compound **6**

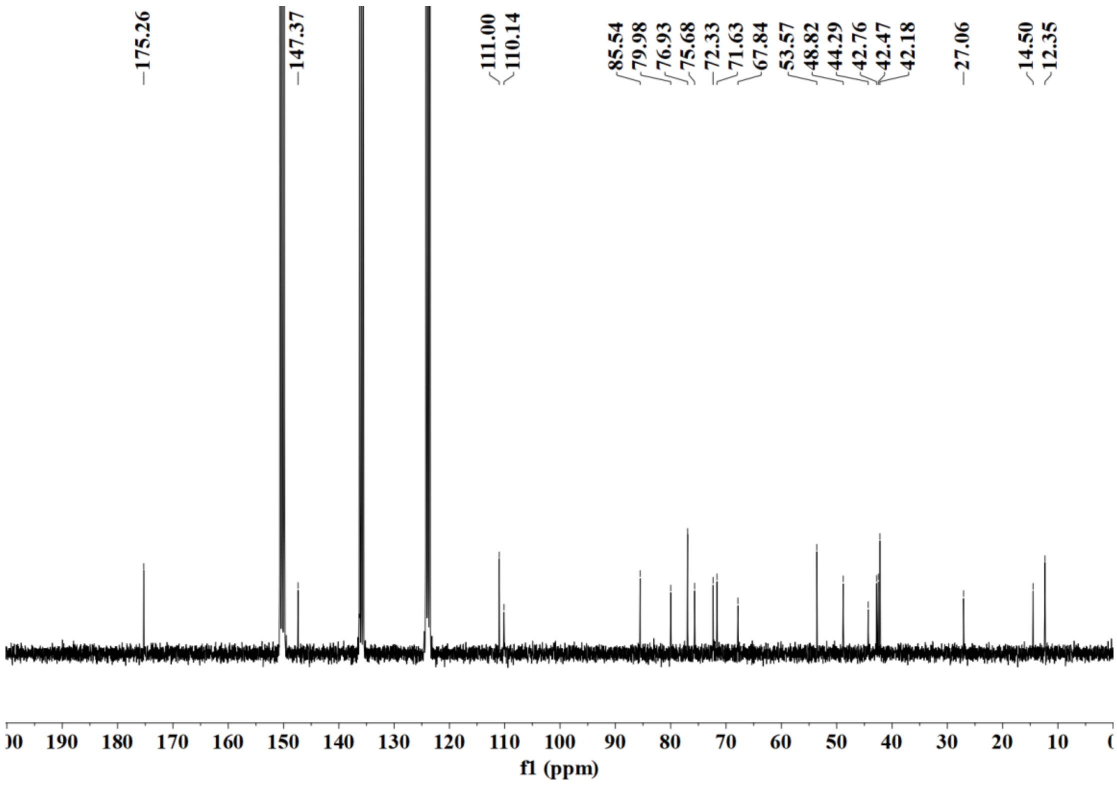

Figure S65. ¹³C NMR spectrum of compound **6**

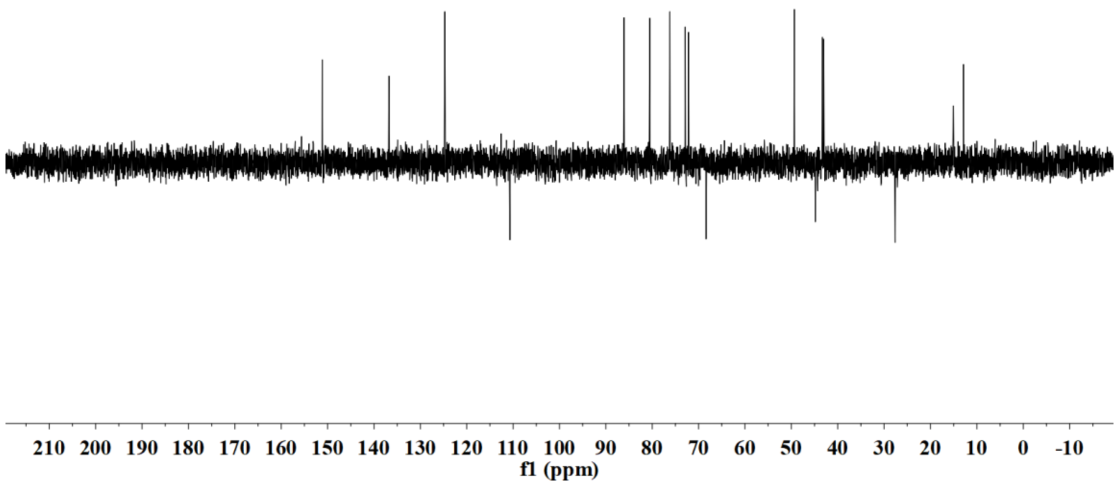

Figure S66. DEPT-135 spectrum of compound **6**

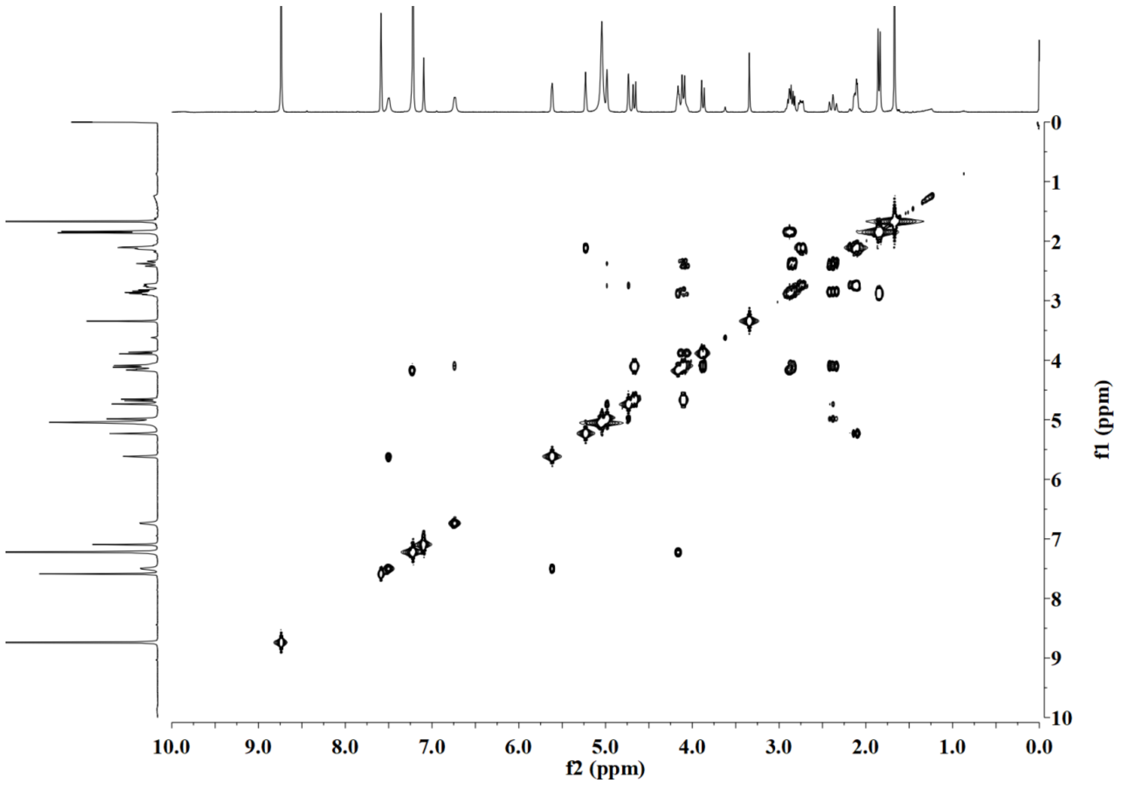

Figure S67. ^{1}H-^{1}H COSY spectrum of compound **6**

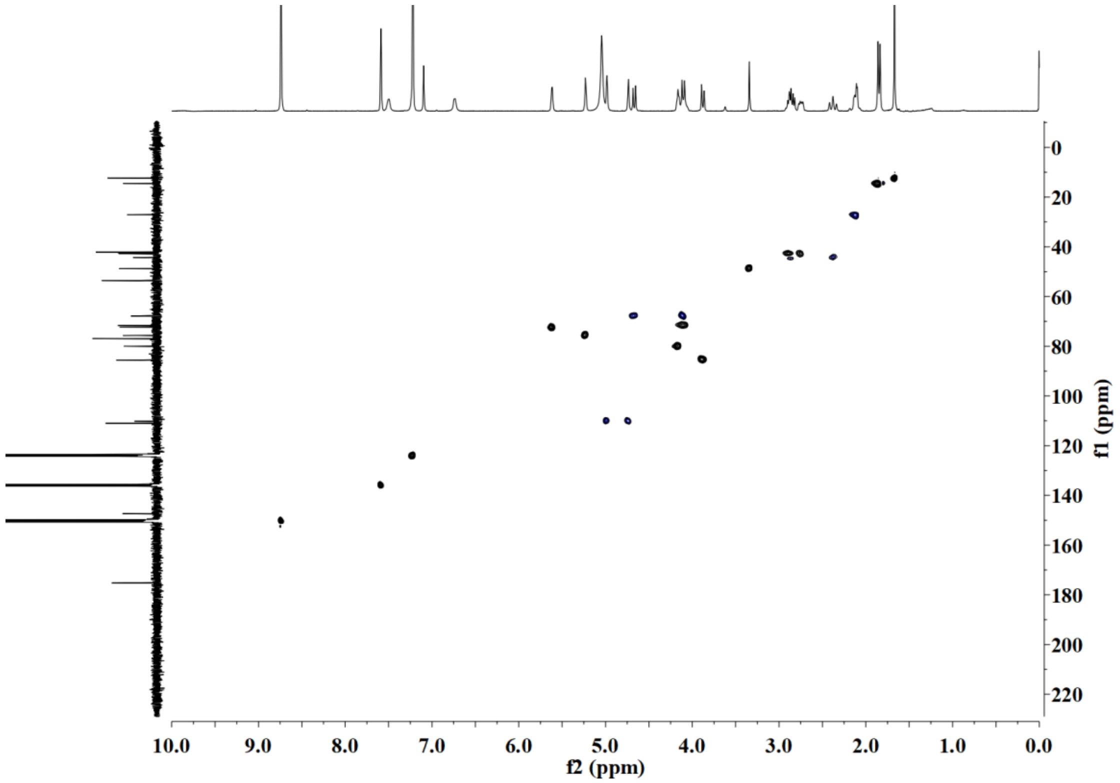

Figure S68. HSQC spectrum of compound **6**

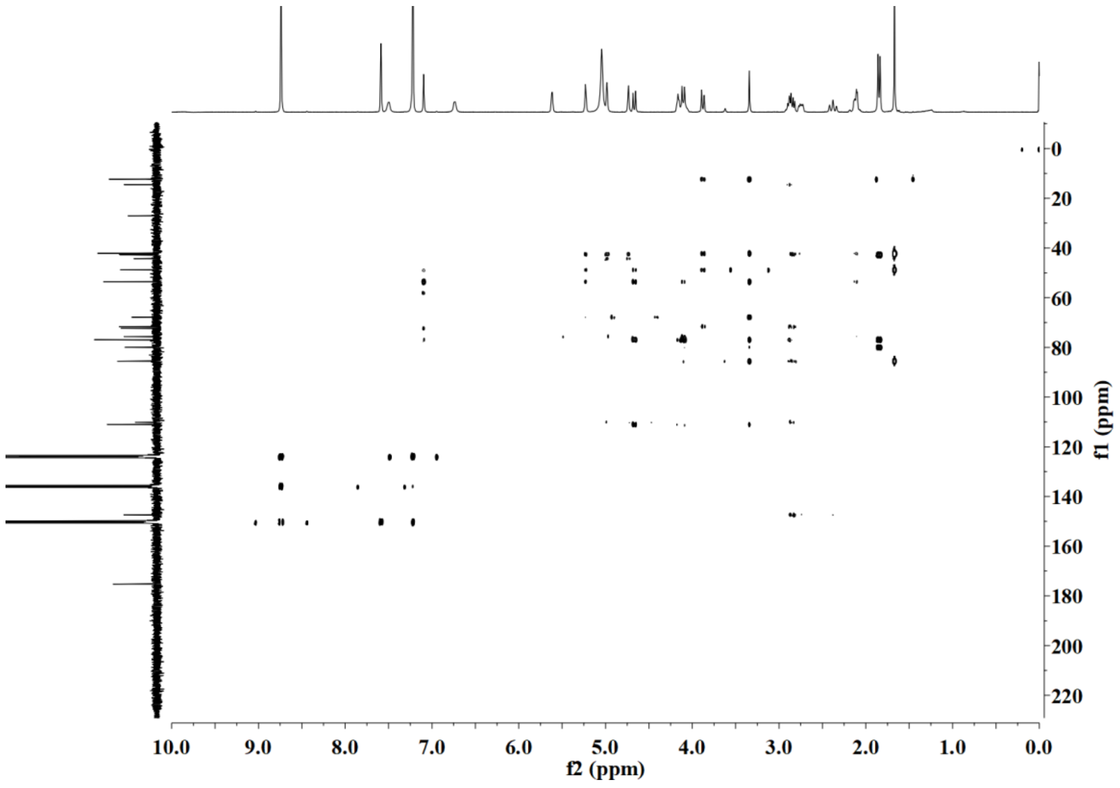

Figure S69. HMBC spectrum of compound **6**

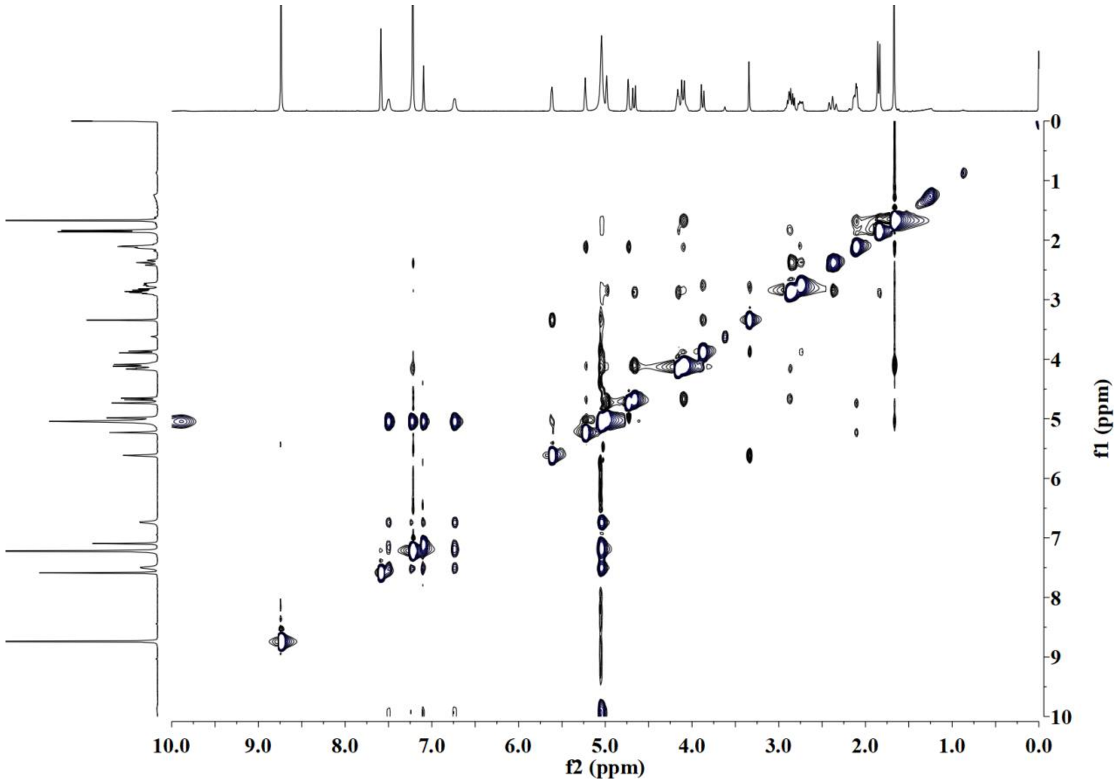

Figure S70. NOESY spectrum of compound **6**

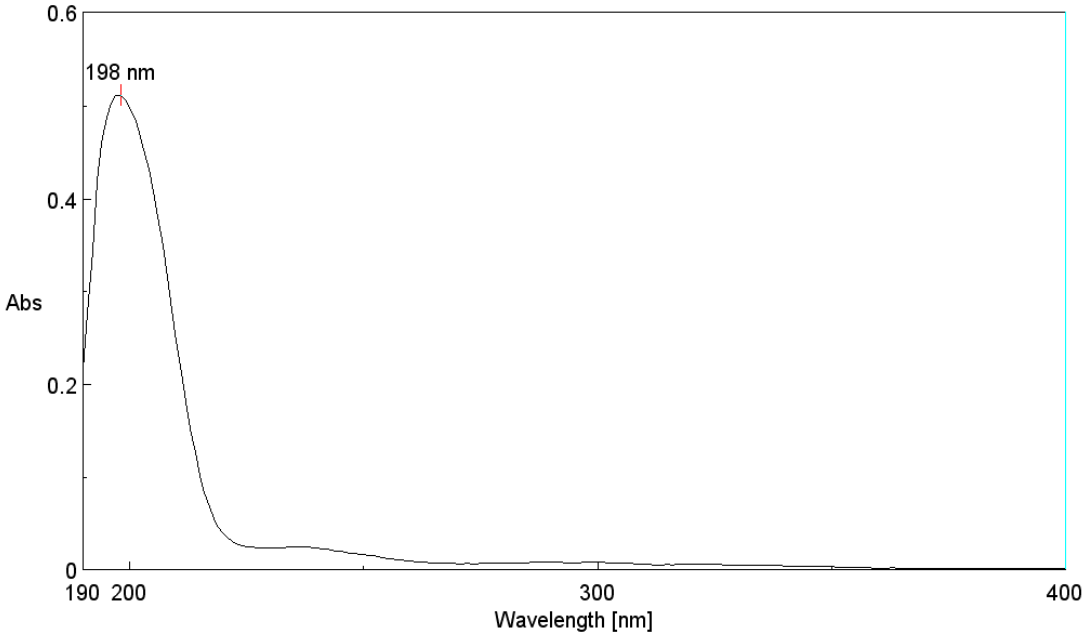

Figure S71. UV spectrum of compound **7**

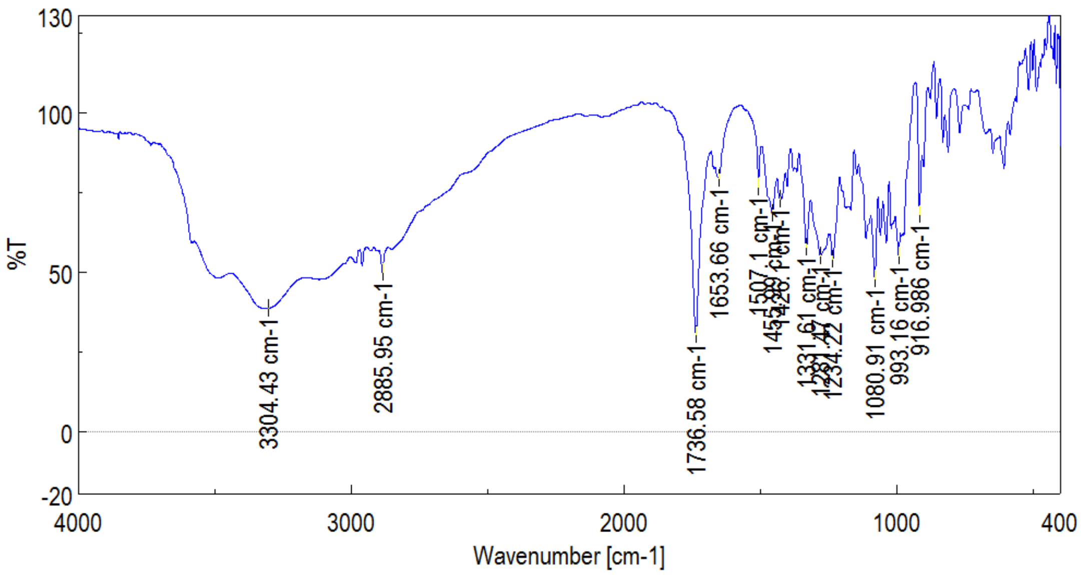

Figure S72. IR (KBr disc) spectrum of compound **7**

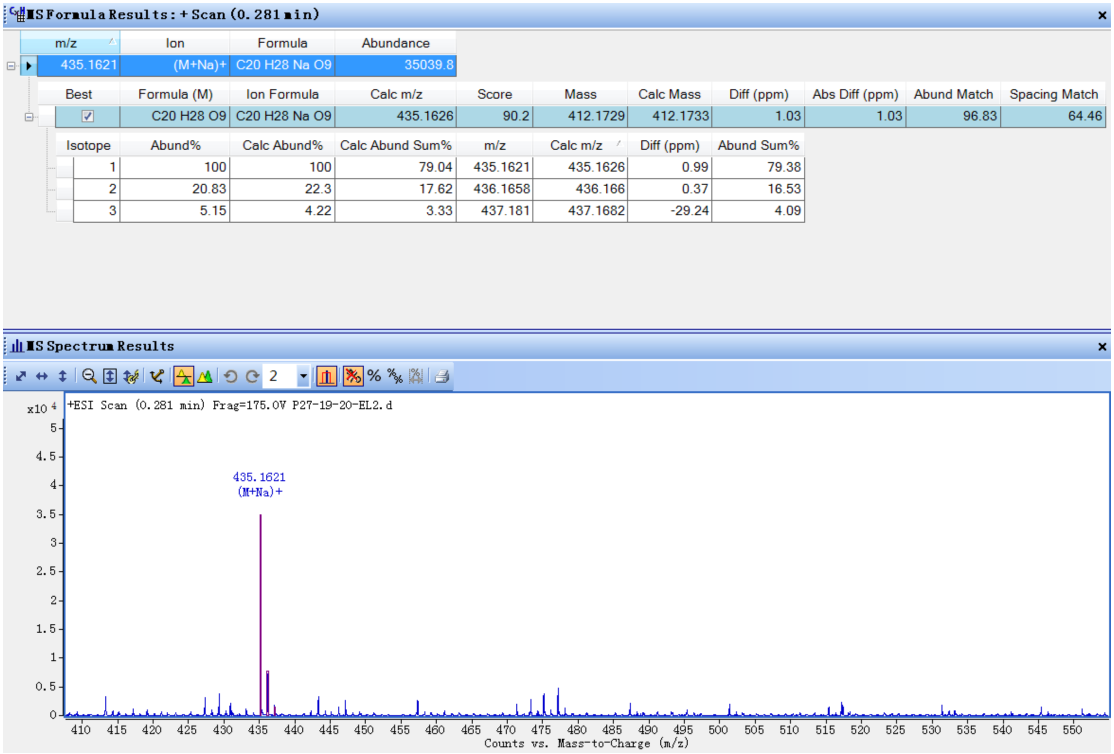

Figure S73. HR-ESI-MS spectrum of compound **7**

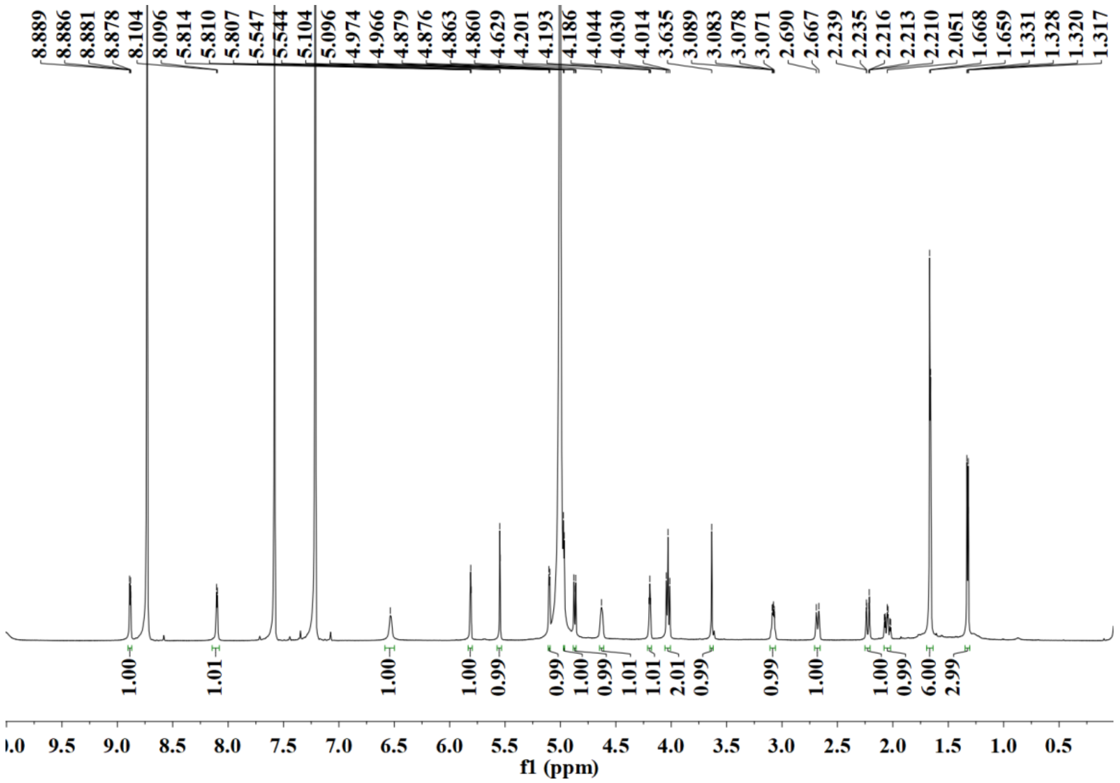

Figure S74. ^{1}H NMR spectrum of compound **7**

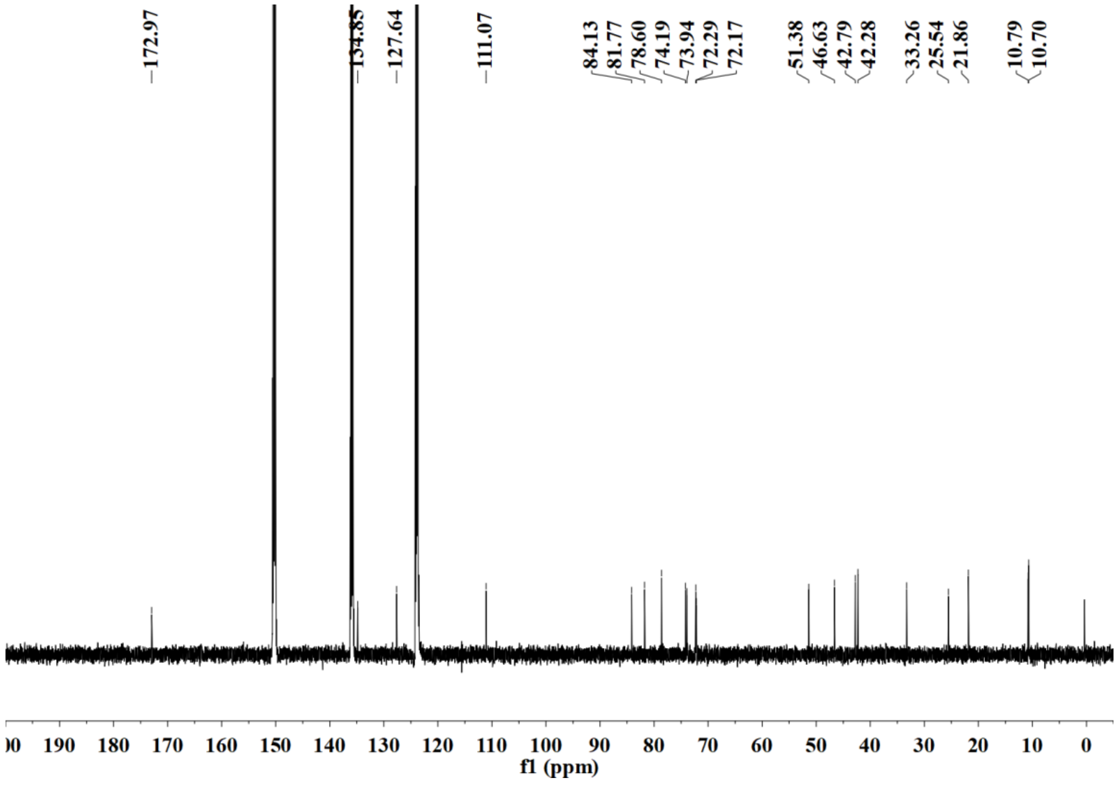

Figure S75. ^{13}C NMR spectrum of compound **7**

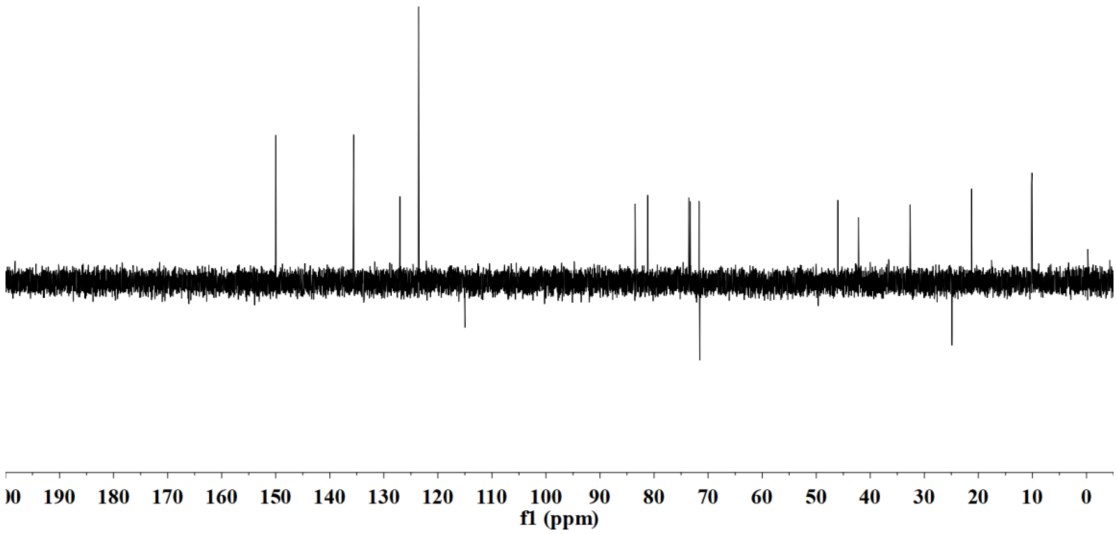

Figure S76. DEPT-135 spectrum of compound **7**

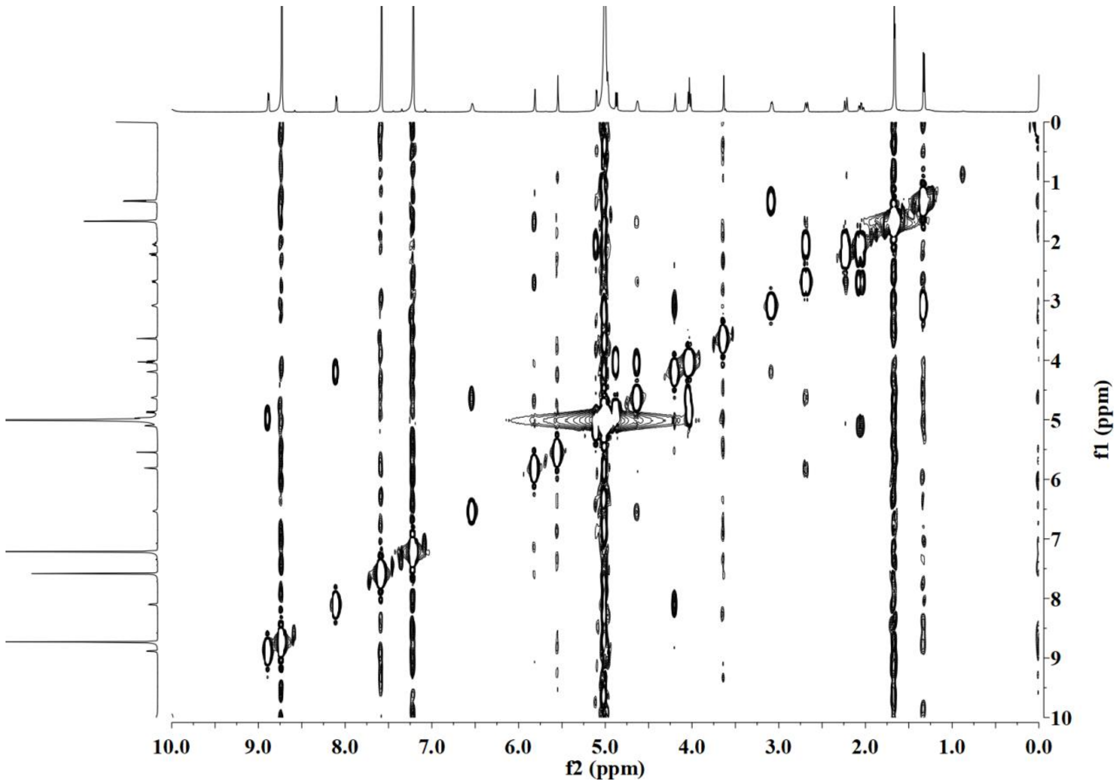

Figure S77. ^{1}H-^{1}H COSY spectrum of compound **7**

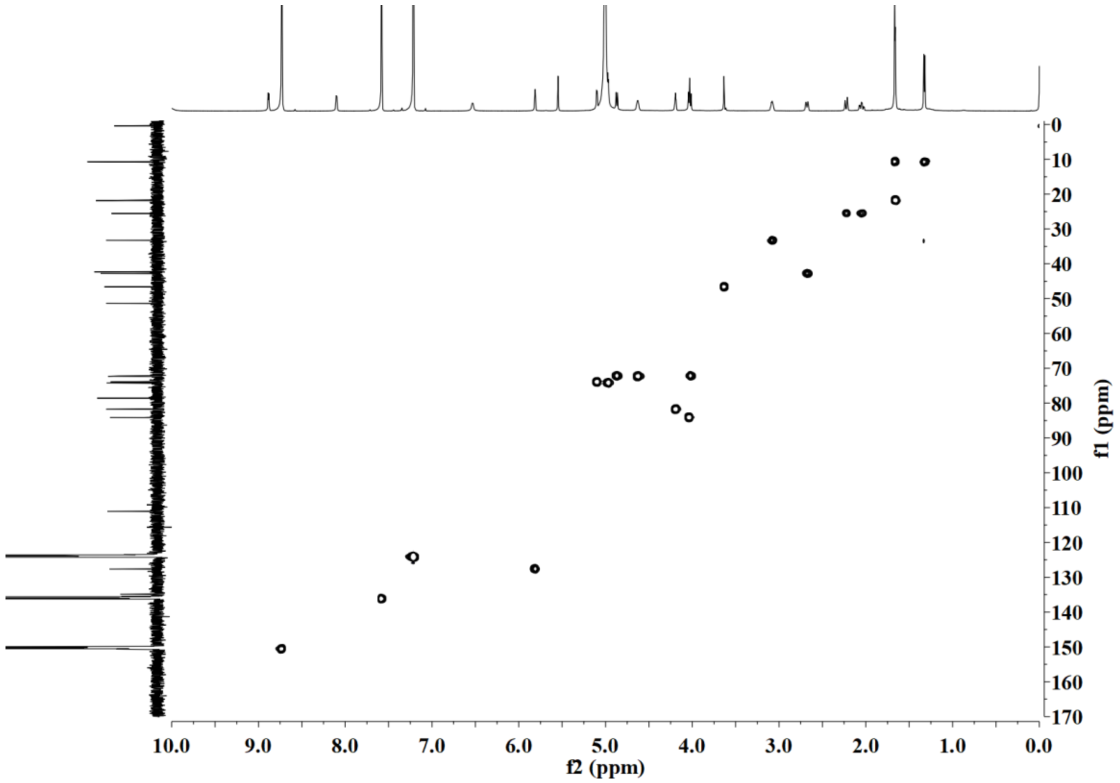

Figure S78. HSQC spectrum of compound **7**

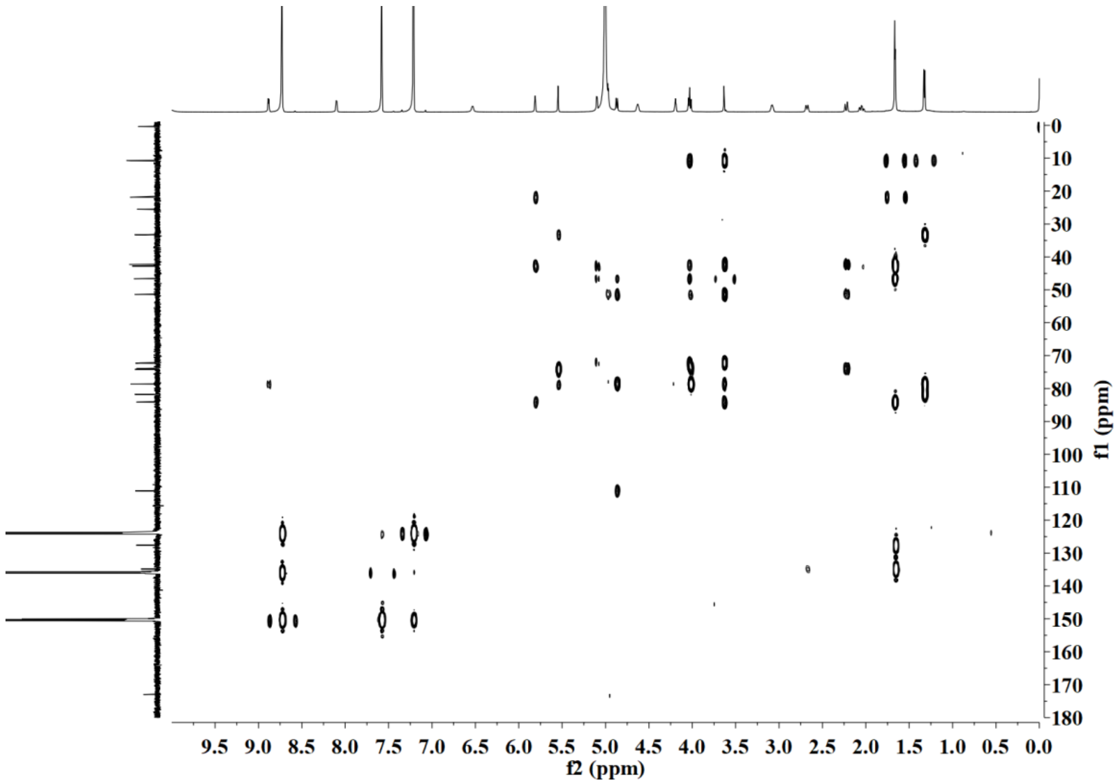

Figure S79. HMBC spectrum of compound **7**

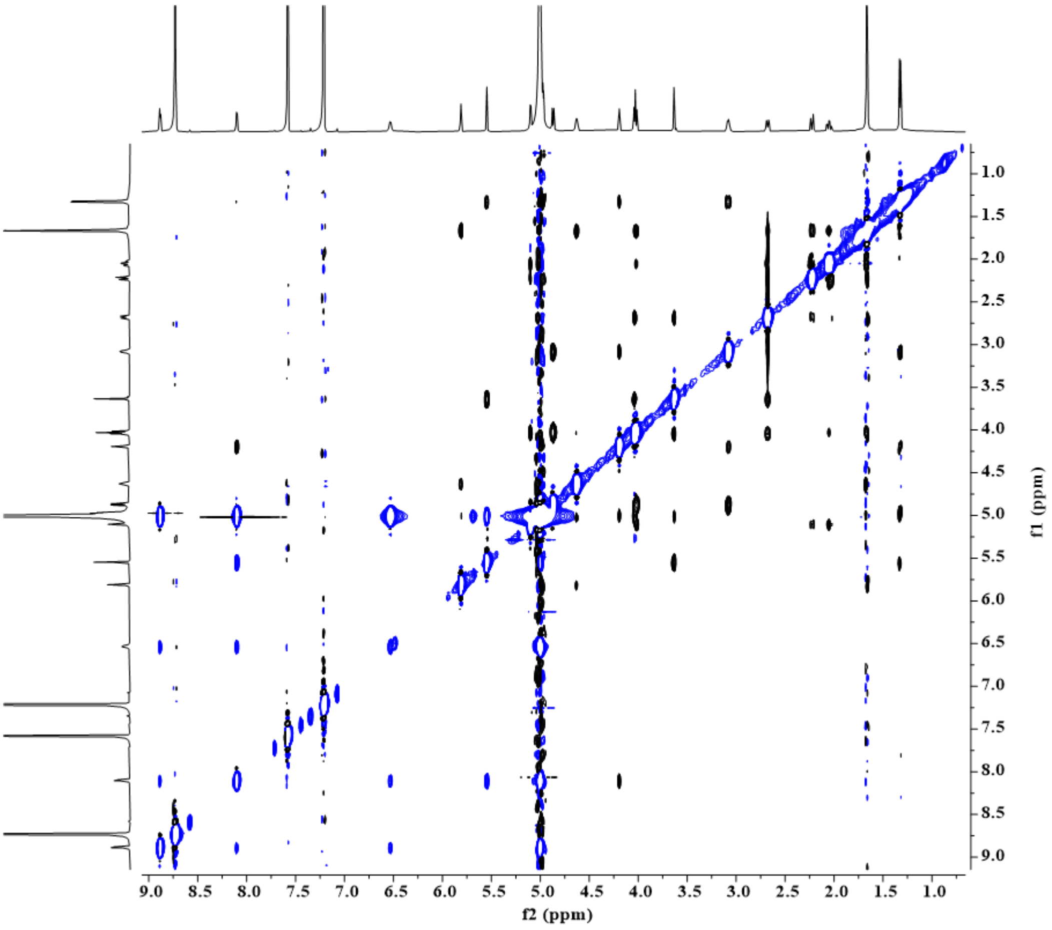

Figure S80. NOESY spectrum of compound **7**

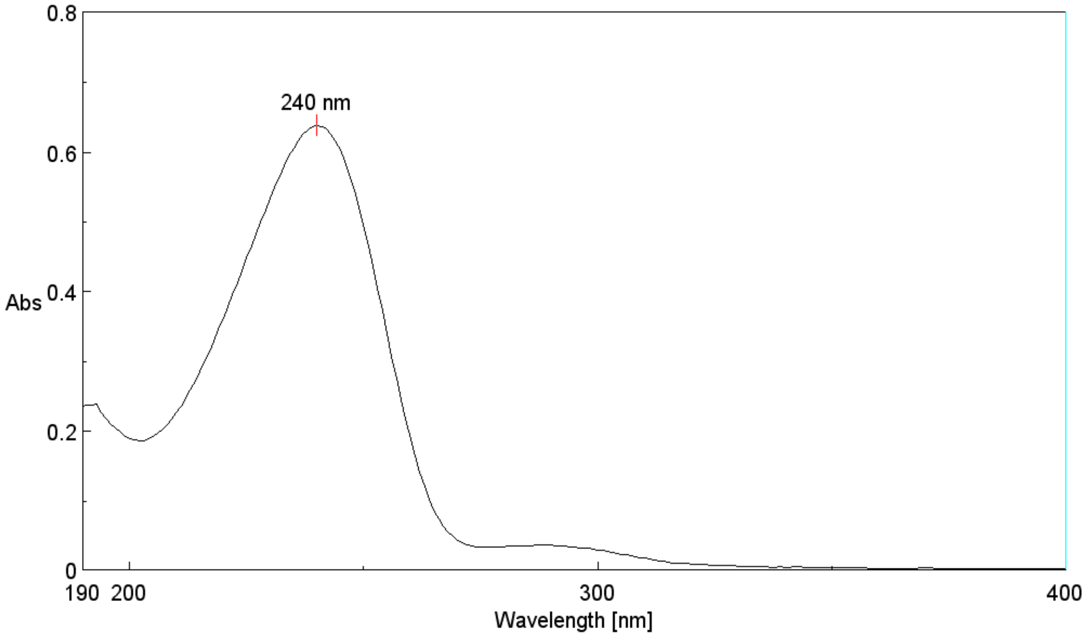

Figure S81. UV spectrum of compound **8**

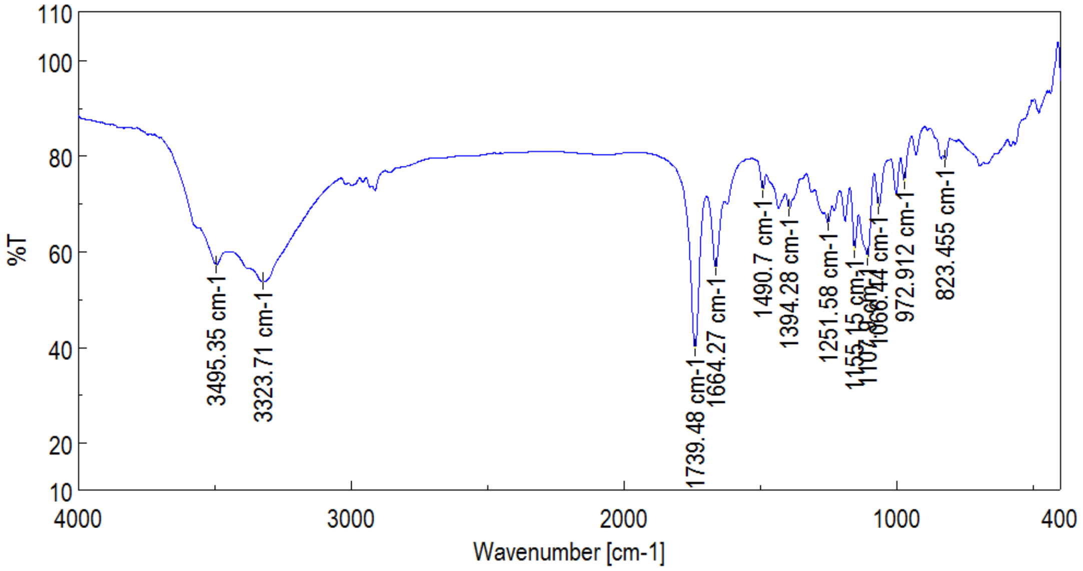

Figure S82. IR (KBr disc) spectrum of compound **8**

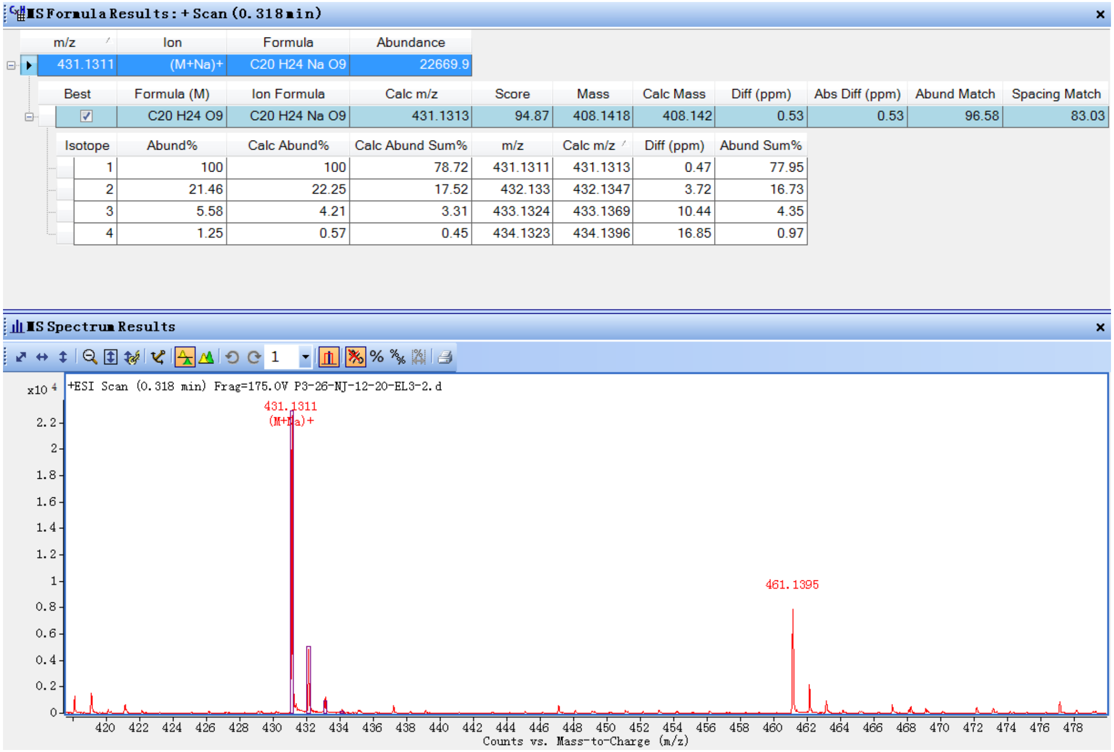

m/z	Ion	Formula	Abundance
431.1311	(M+Na)+	C20 H24 Na O9	22669.9

Best	Formula (M)	Ion Formula	Calc m/z	Score	Mass	Calc Mass	Diff (ppm)	Abs Diff (ppm)	Abund Match	Spacing Match
✓	C20 H24 O9	C20 H24 Na O9	431.1313	94.87	408.1418	408.142	0.53	0.53	96.58	83.03

Isotope	Abund%	Calc Abund%	Calc Abund Sum%	m/z	Calc m/z	Diff (ppm)	Abund Sum%
1	100	100	78.72	431.1311	431.1313	0.47	77.95
2	21.46	22.25	17.52	432.133	432.1347	3.72	16.73
3	5.58	4.21	3.31	433.1324	433.1369	10.44	4.35
4	1.25	0.57	0.45	434.1323	434.1396	16.85	0.97

Figure S83. HR-ESI-MS spectrum of compound **8**

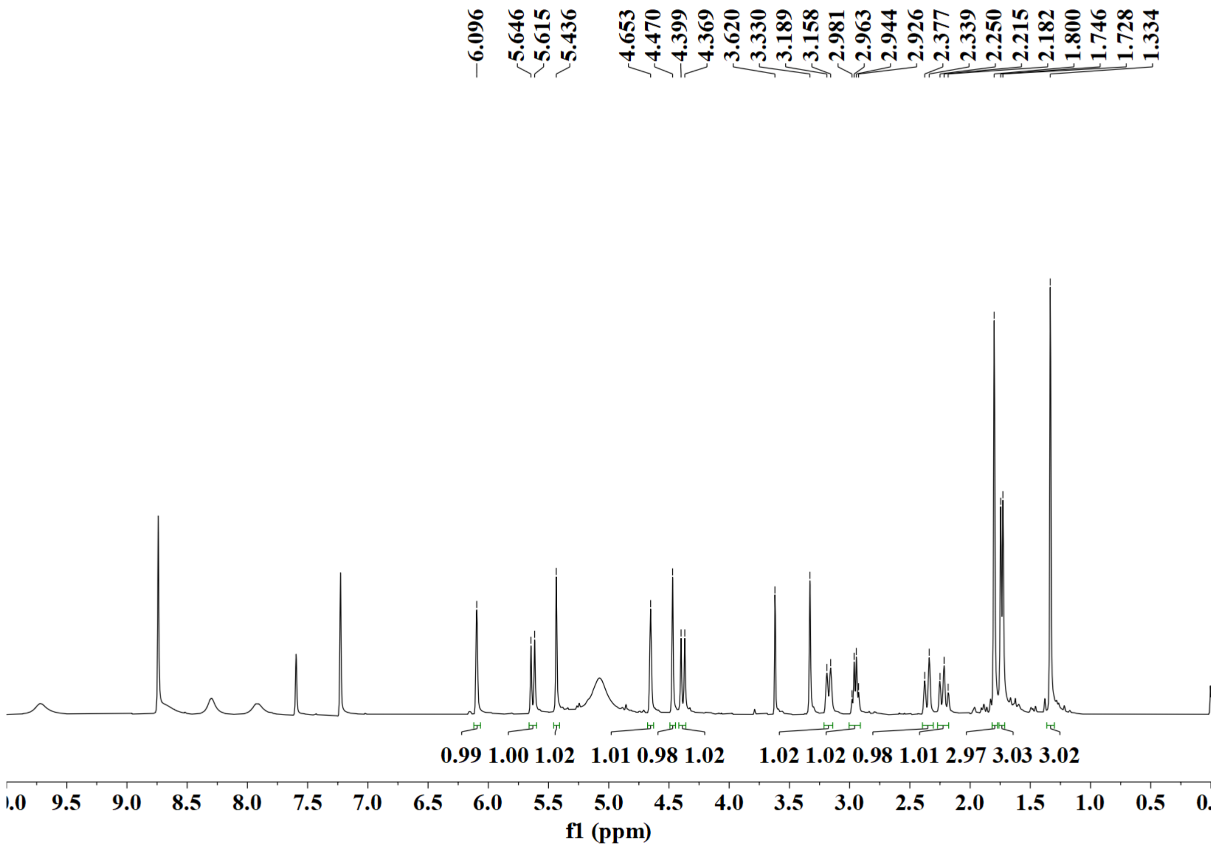

Figure S84. ^{1}H NMR spectrum of compound **8**

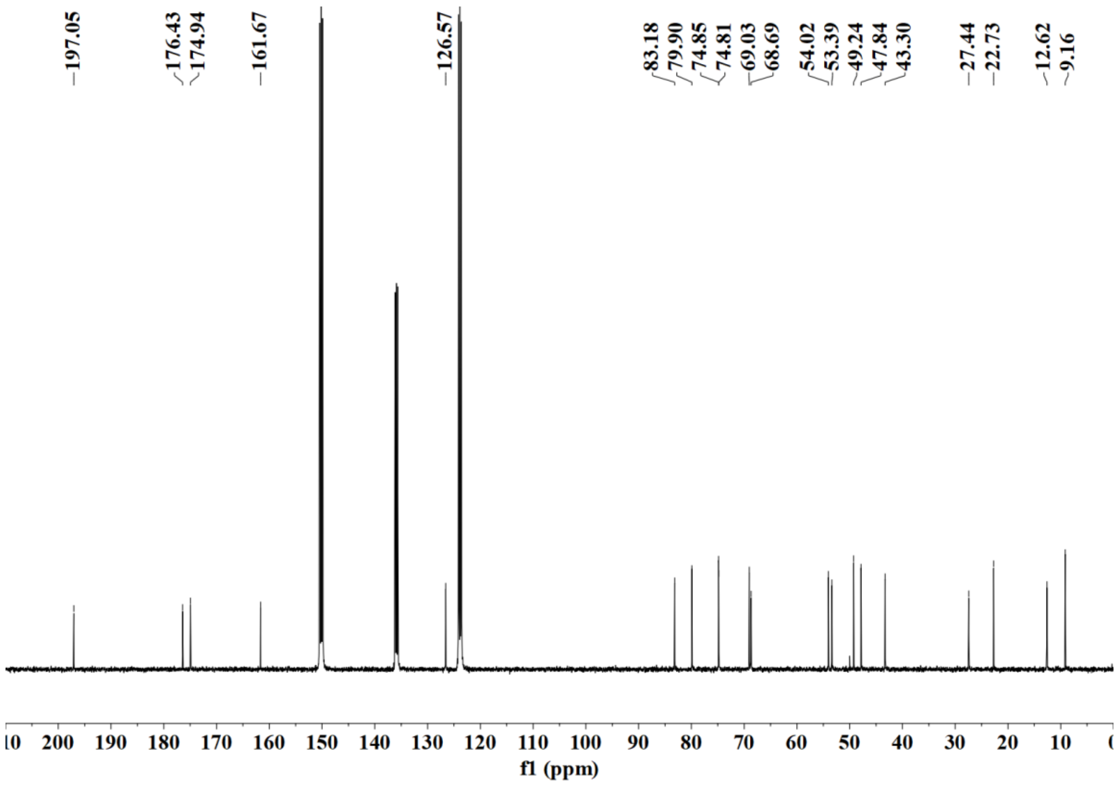

Figure S85. ^{13}C NMR spectrum of compound **8**

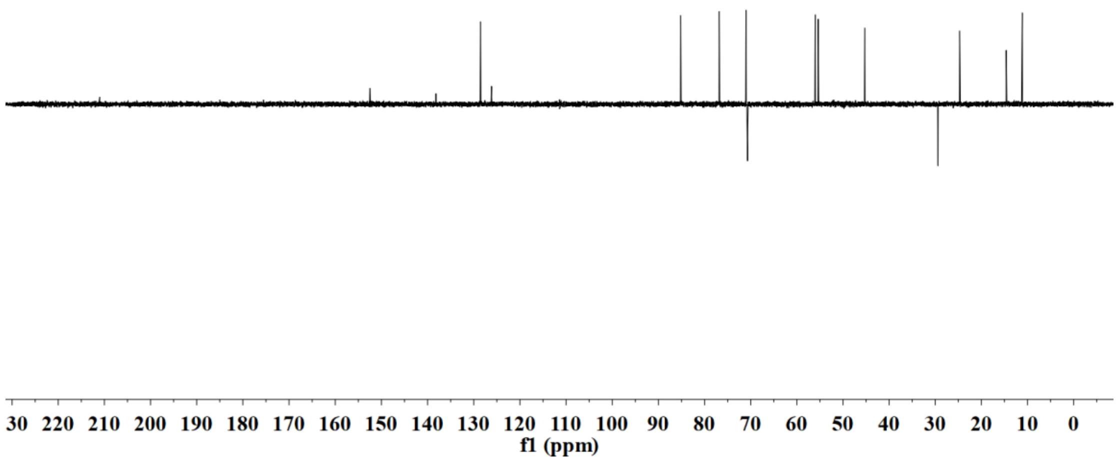

Figure S86. DEPT-135 spectrum of compound **8**

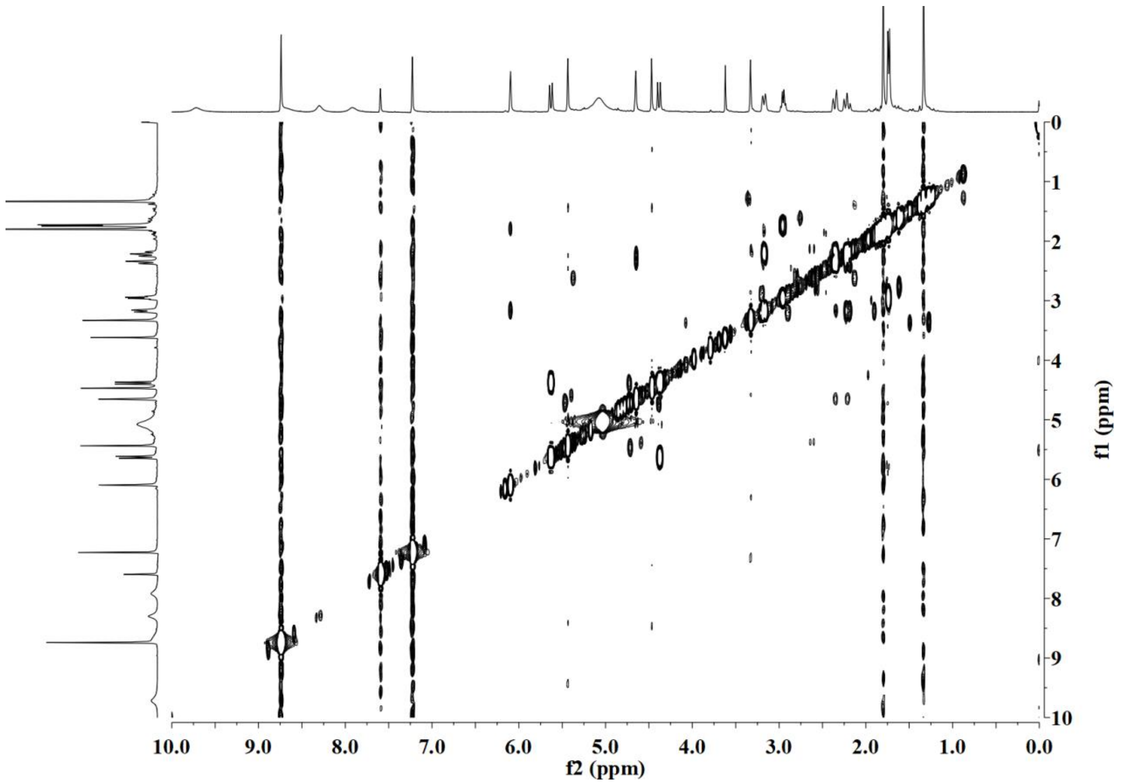

Figure S87. ^{1}H-^{1}H COSY spectrum of compound **8**

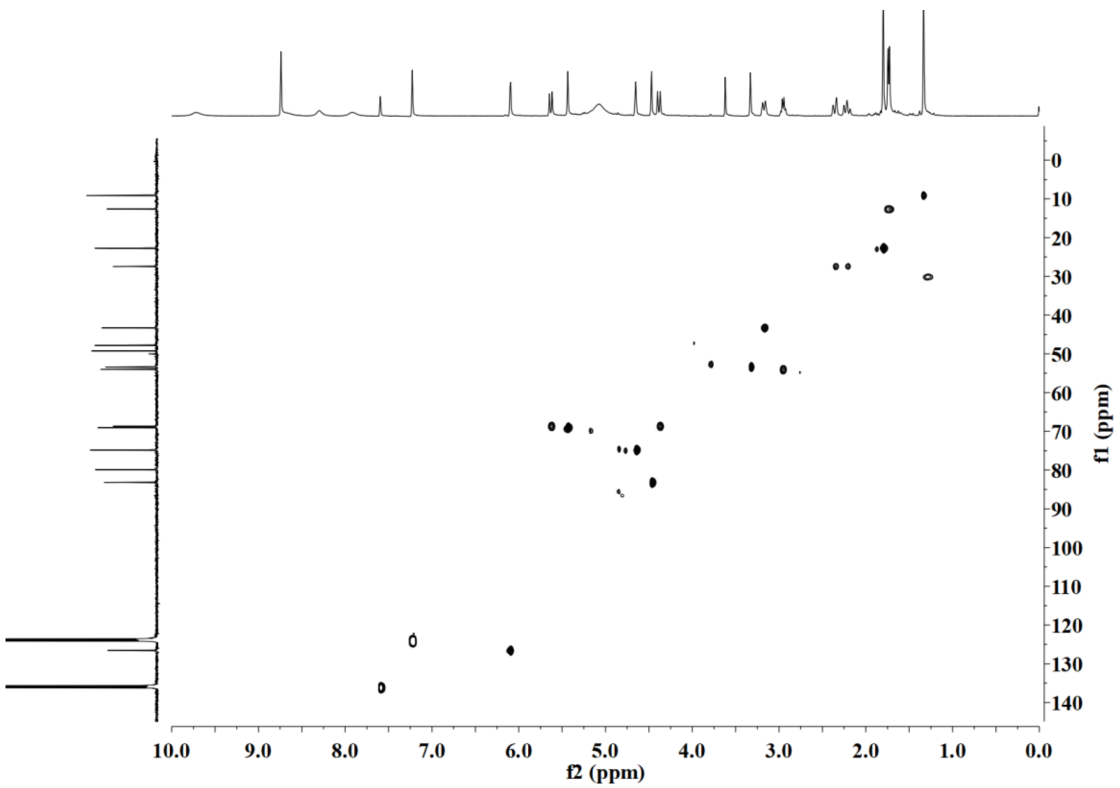

Figure S88. HSQC spectrum of compound **8**

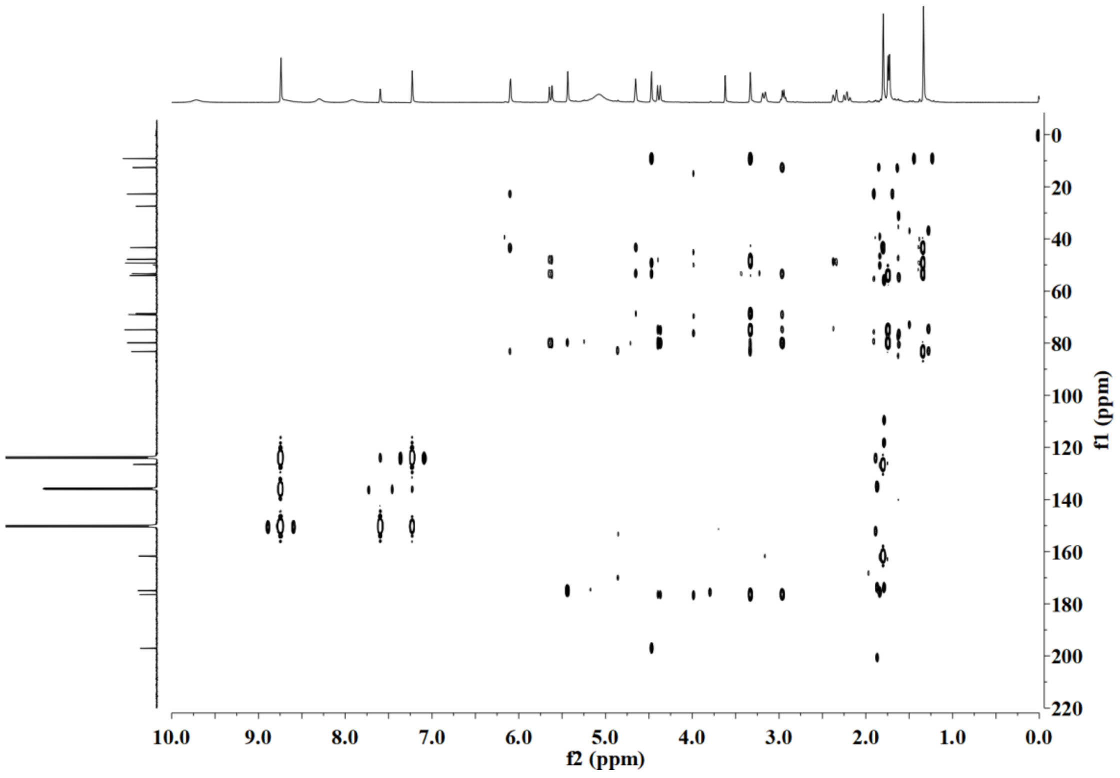

Figure S89. HMBC spectrum of compound **8**

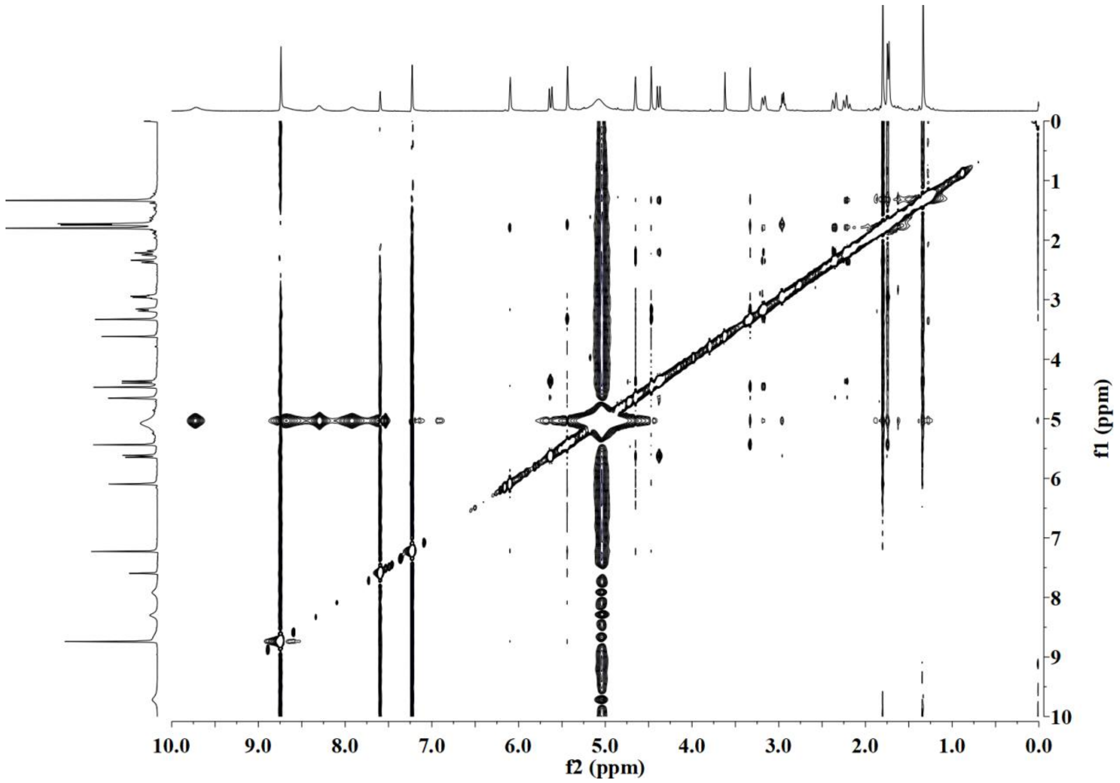

Figure S90. NOESY spectrum of compound **8**

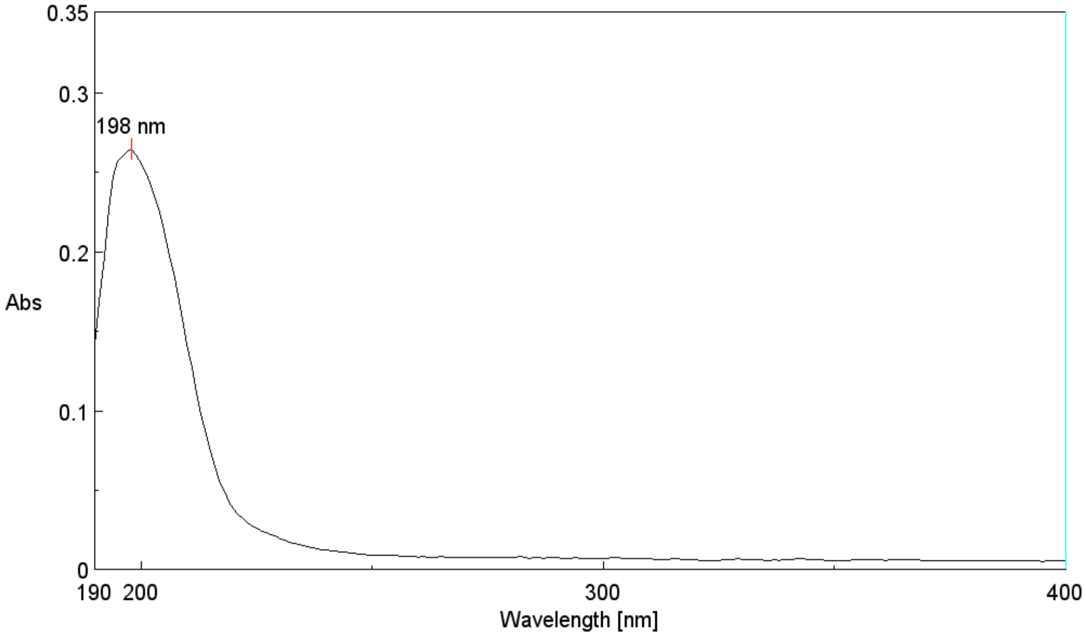

Figure S91. UV spectrum of compound **9**

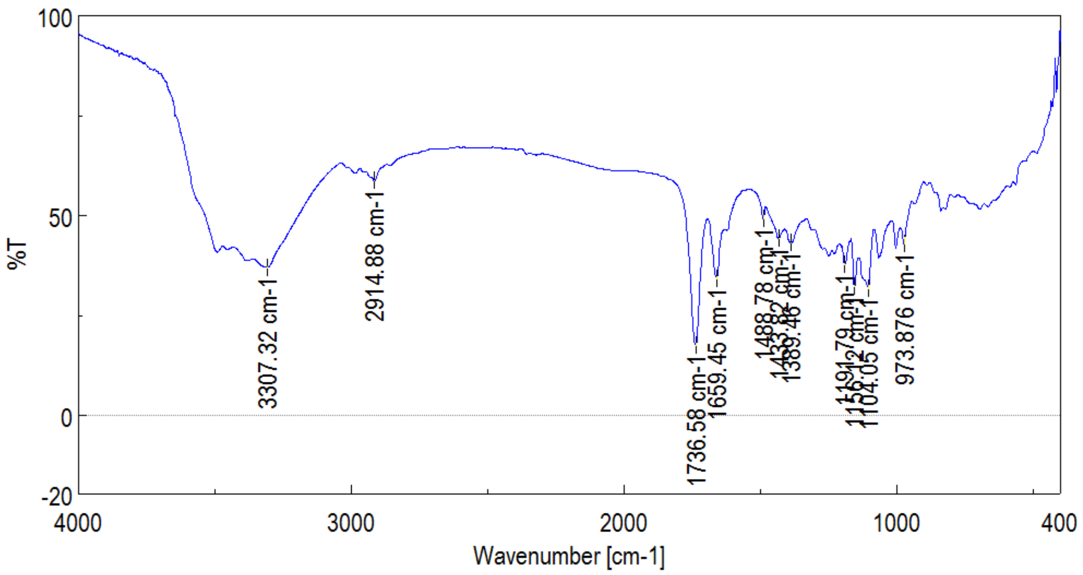

Figure S92. IR (KBr disc) spectrum of compound **9**

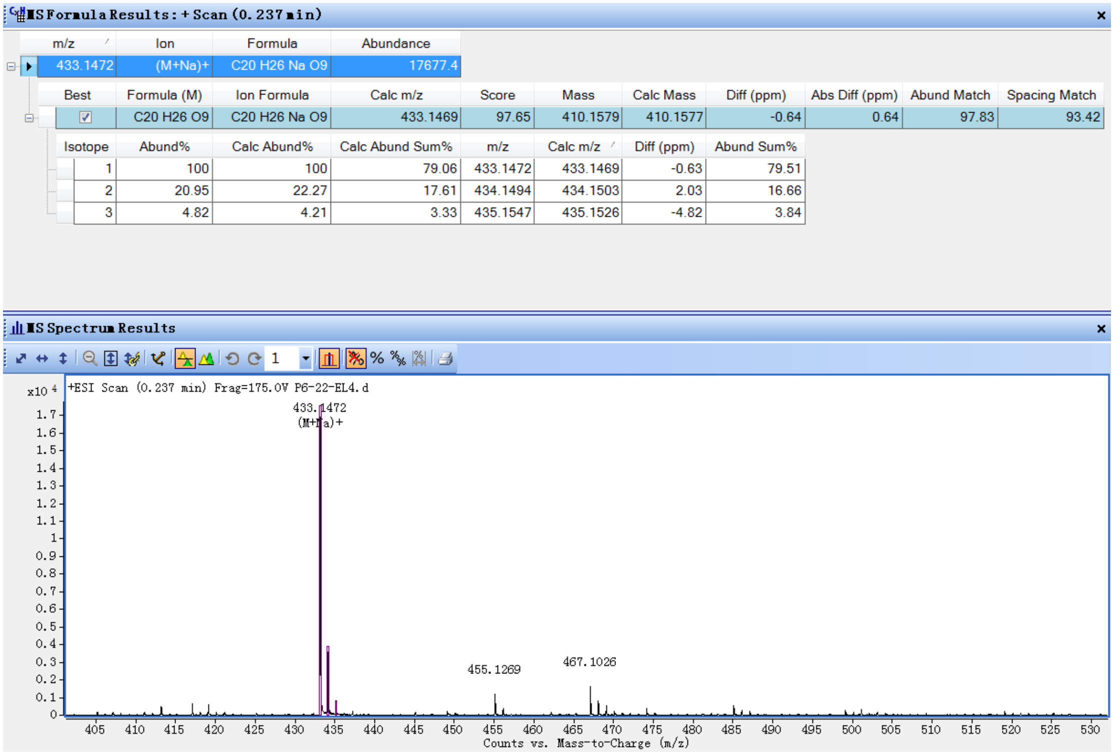

m/z	Ion	Formula	Abundance
433.1472	(M+Na)+	C20 H26 Na O9	17677.4

Best	Formula (M)	Ion Formula	Calc m/z	Score	Mass	Calc Mass	Diff (ppm)	Abs Diff (ppm)	Abund Match	Spacing Match
☑	C20 H26 O9	C20 H26 Na O9	433.1469	97.65	410.1579	410.1577	-0.64	0.64	97.83	93.42

Isotope	Abund%	Calc Abund%	Calc Abund Sum%	m/z	Calc m/z	Diff (ppm)	Abund Sum%
1	100	100	79.06	433.1472	433.1469	-0.63	79.51
2	20.95	22.27	17.61	434.1494	434.1503	2.03	16.66
3	4.82	4.21	3.33	435.1547	435.1526	-4.82	3.84

Figure S93. HR-ESI-MS spectrum of compound **9**

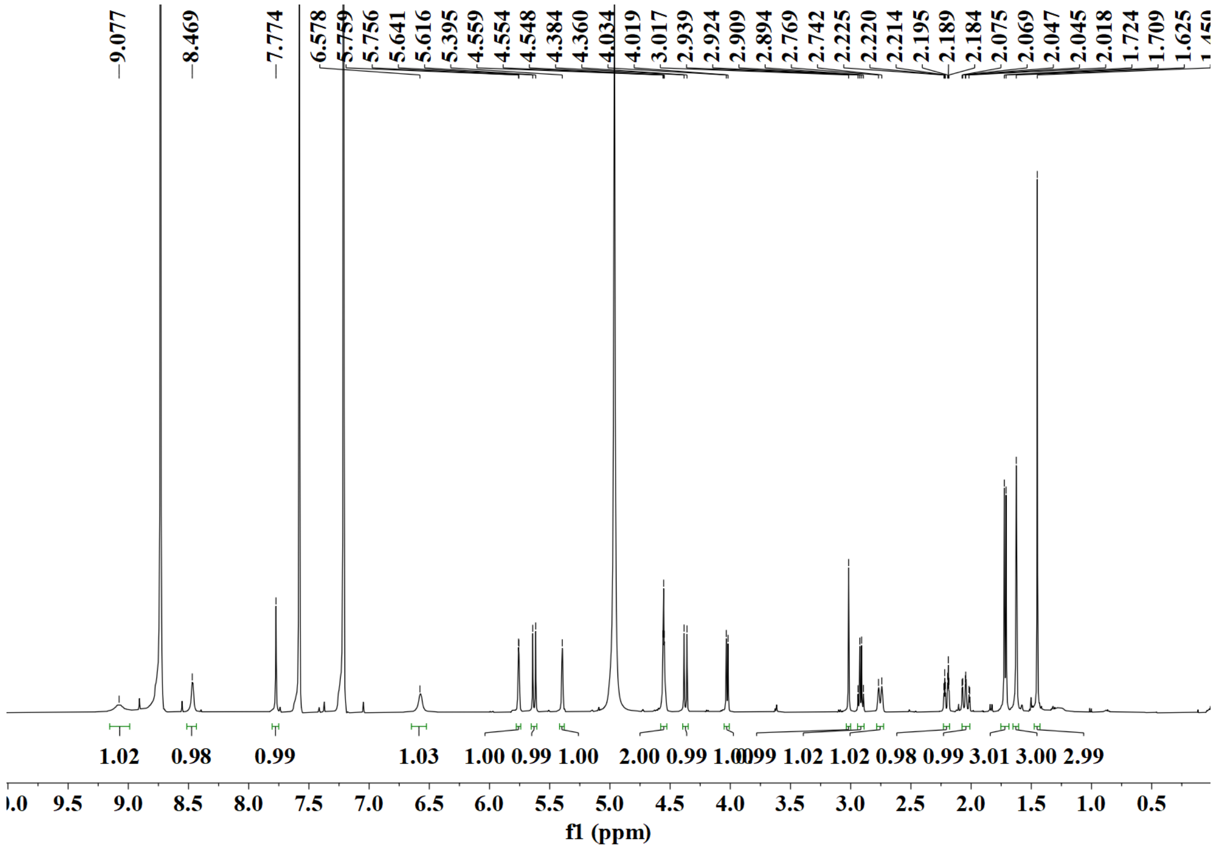

Figure S94. ^{1}H NMR spectrum of compound **9**

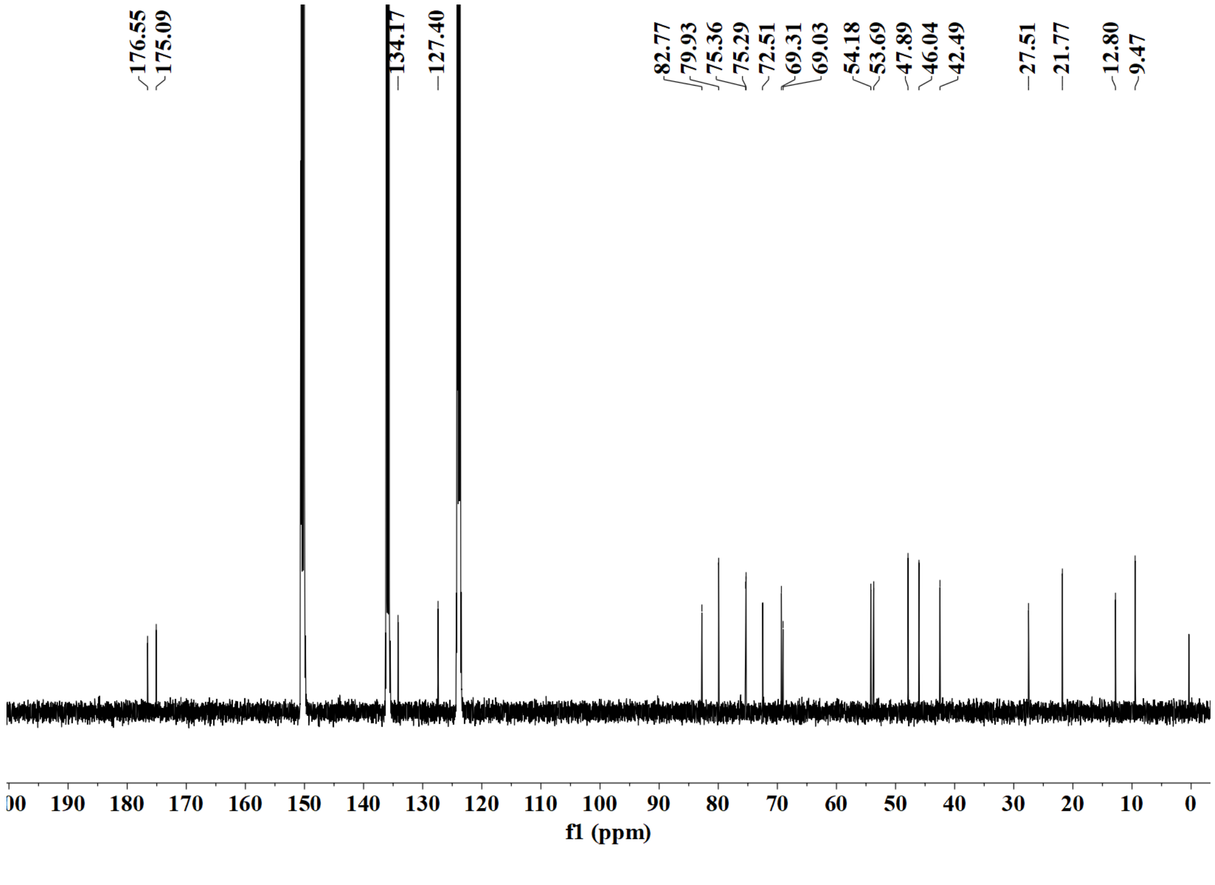

Figure S95. ^{13}C NMR spectrum of compound **9**

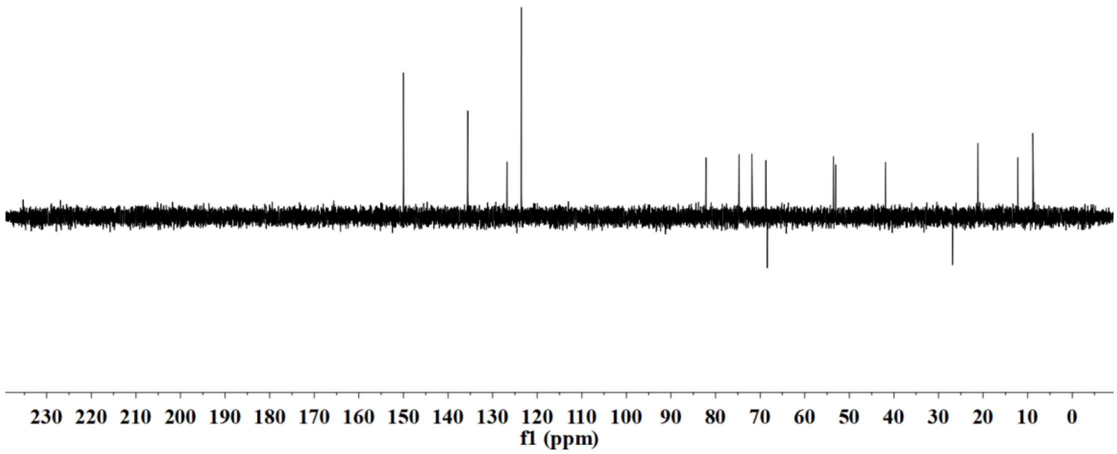

Figure S96. DEPT-135 spectrum of compound **9**

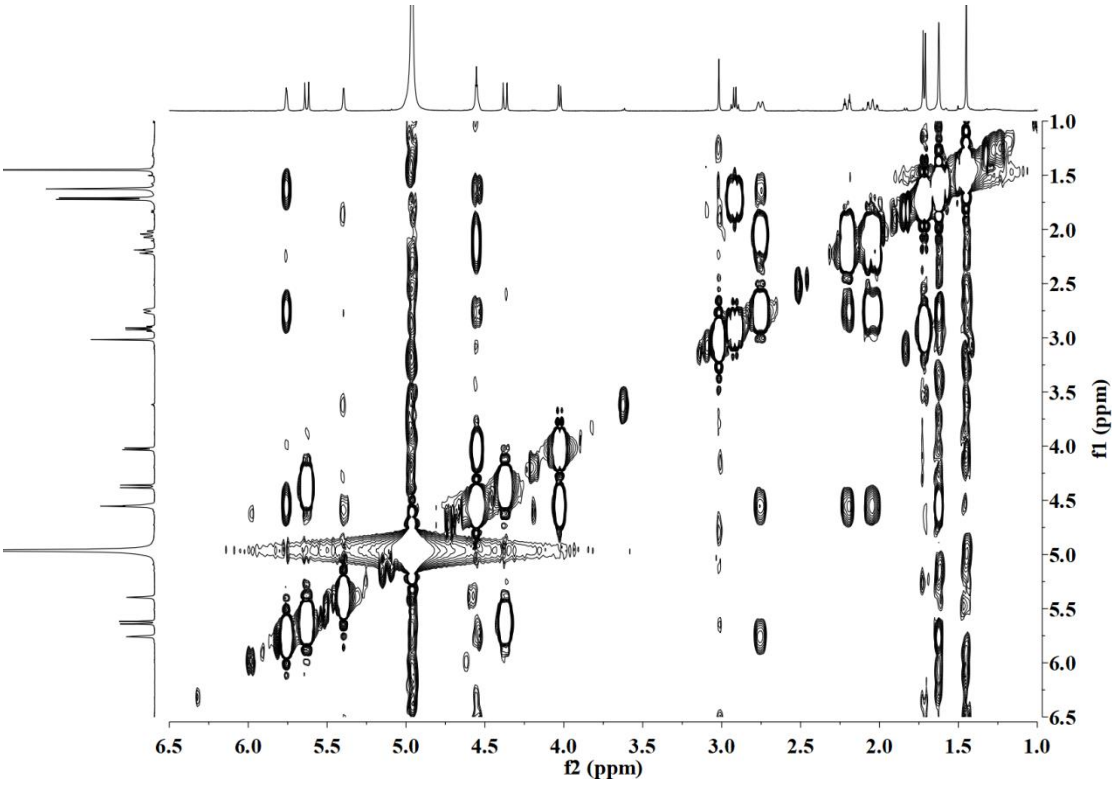

Figure S97. ^{1}H-^{1}H COSY spectrum of compound **9**

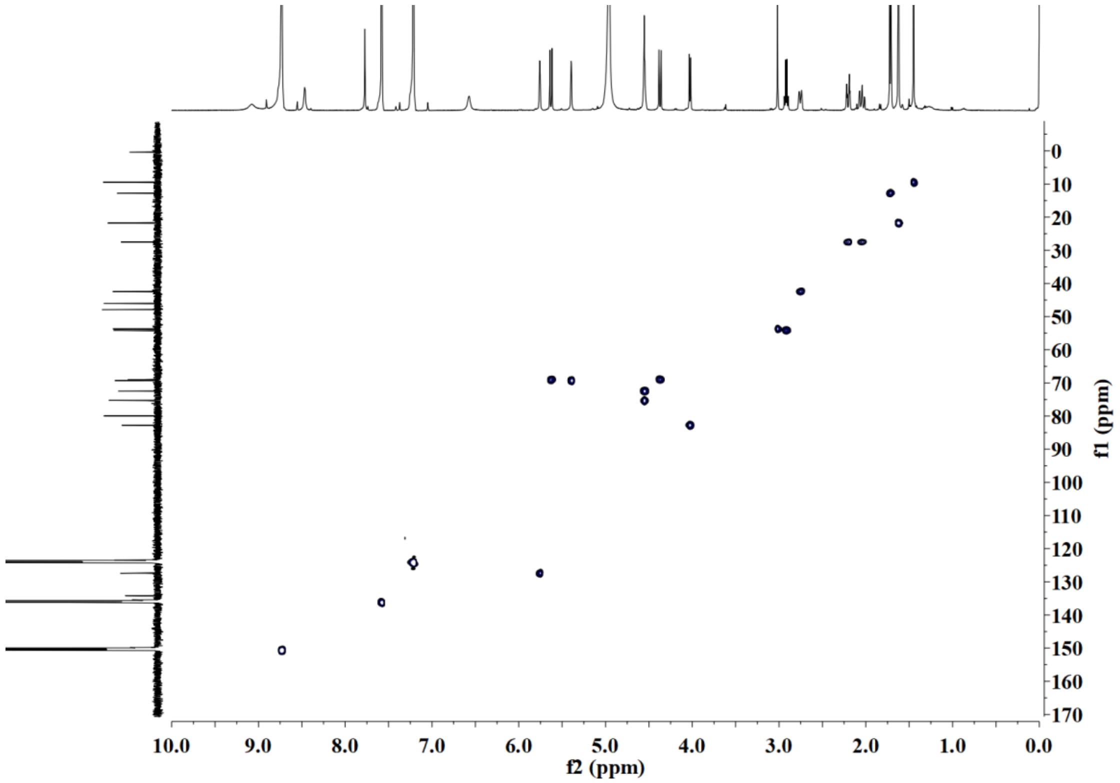

Figure S98. HSQC spectrum of compound **9**

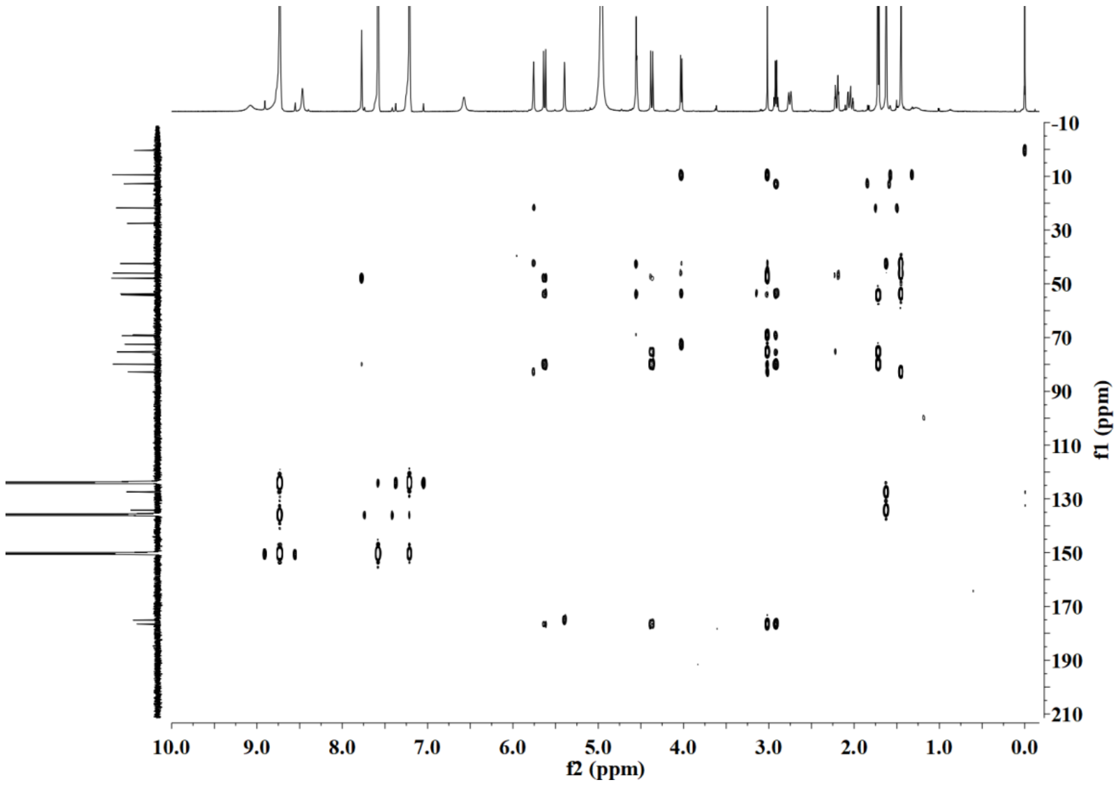

Figure S99. HMBC spectrum of compound **9**

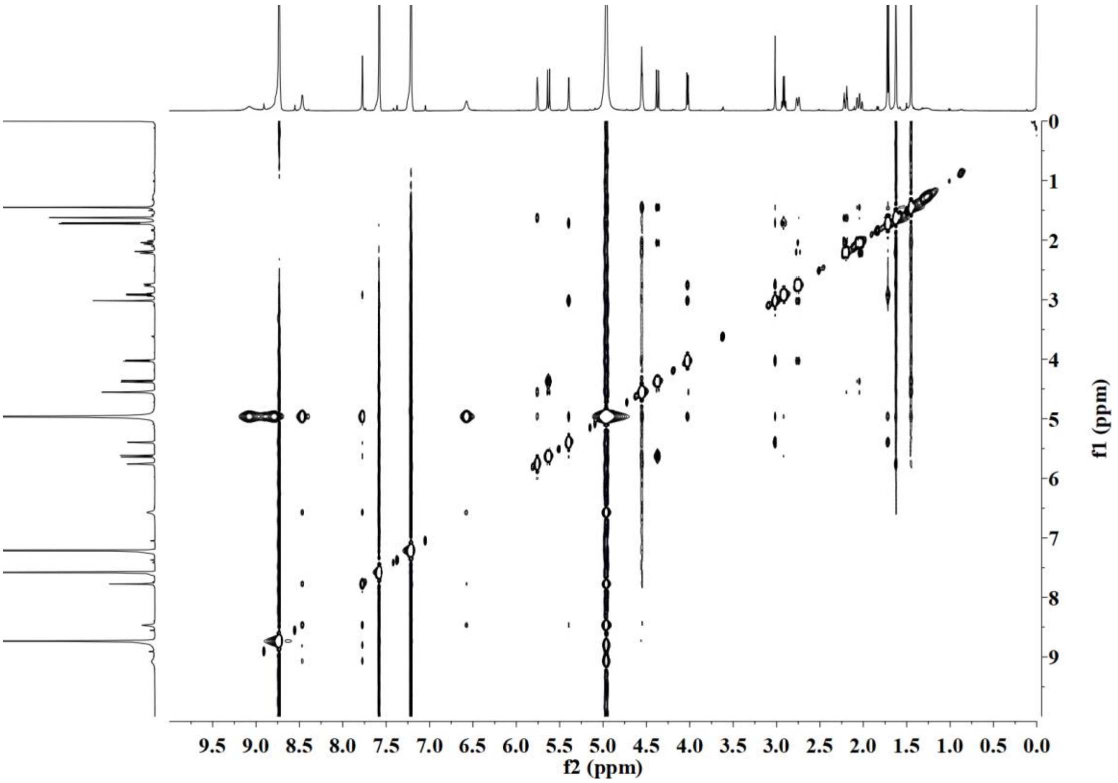

Figure S100. NOESY spectrum of compound **9**

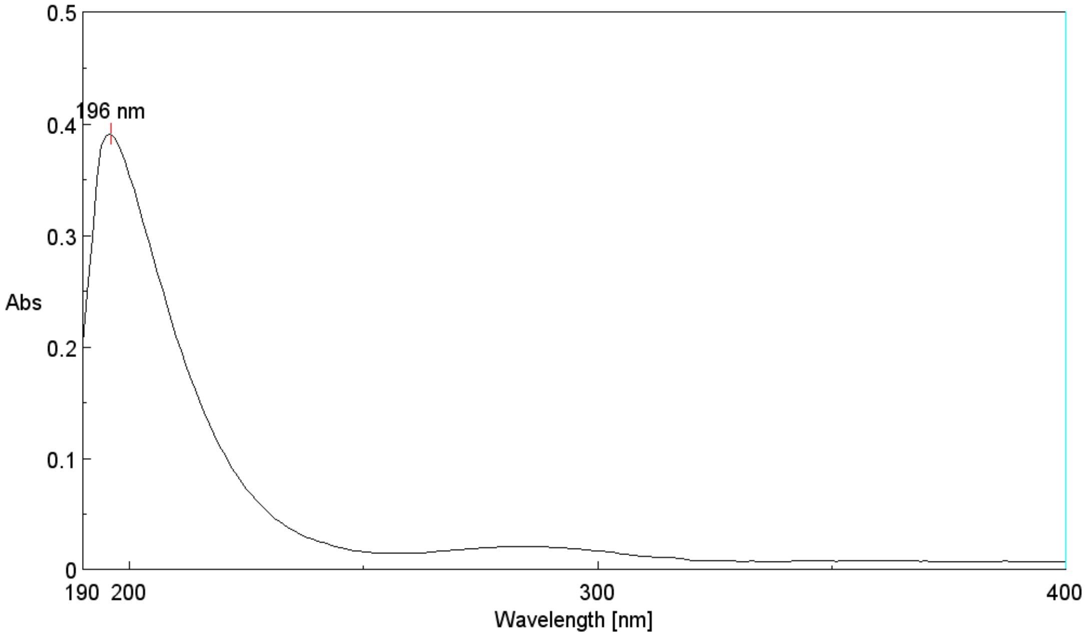

Figure S101. UV spectrum of compound **10**

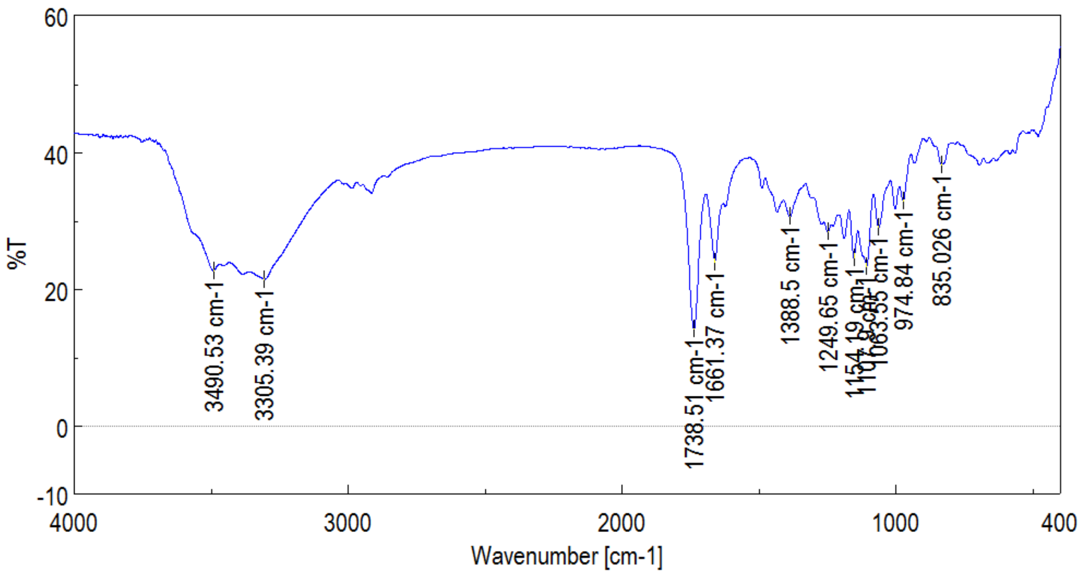

Figure S102. IR (KBr disc) spectrum of compound **10**

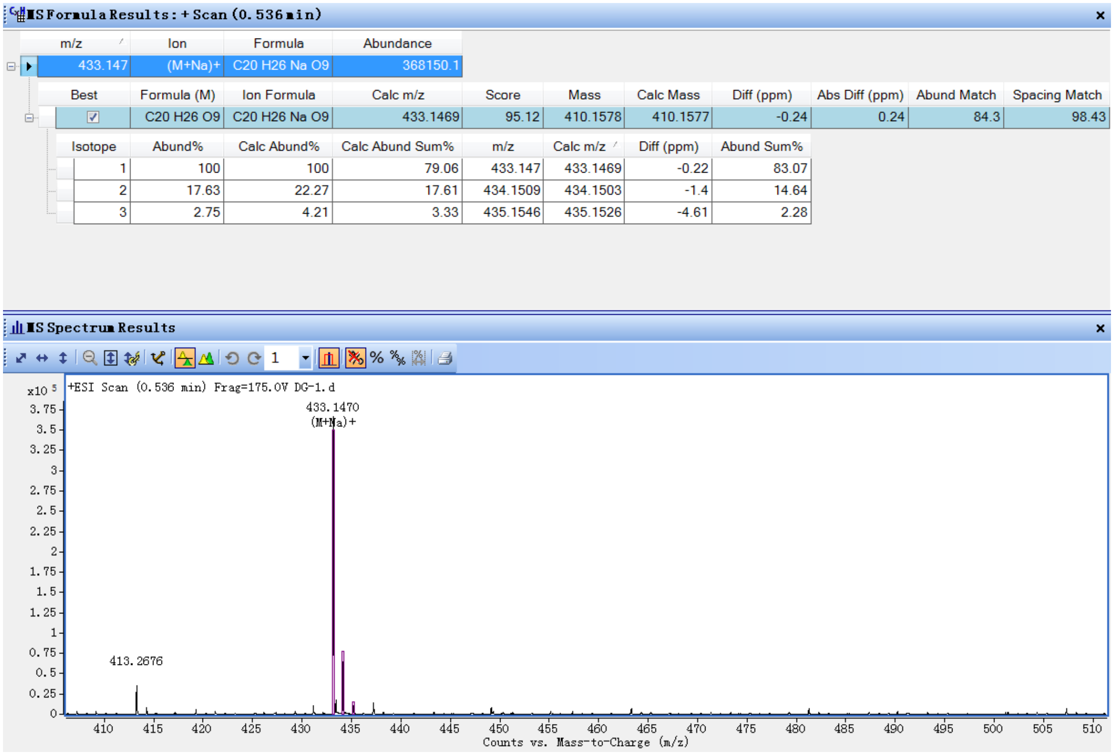

m/z	Ion	Formula	Abundance
433.147	(M+Na)+	C20 H26 Na O9	368150.1

Best	Formula (M)	Ion Formula	Calc m/z	Score	Mass	Calc Mass	Diff (ppm)	Abs Diff (ppm)	Abund Match	Spacing Match
✓	C20 H26 O9	C20 H26 Na O9	433.1469	95.12	410.1578	410.1577	-0.24	0.24	84.3	98.43

Isotope	Abund%	Calc Abund%	Calc Abund Sum%	m/z	Calc m/z	Diff (ppm)	Abund Sum%
1	100	100	79.06	433.147	433.1469	-0.22	83.07
2	17.63	22.27	17.61	434.1509	434.1503	-1.4	14.64
3	2.75	4.21	3.33	435.1546	435.1526	-4.61	2.28

Figure S103. HR-ESI-MS spectrum of compound **10**

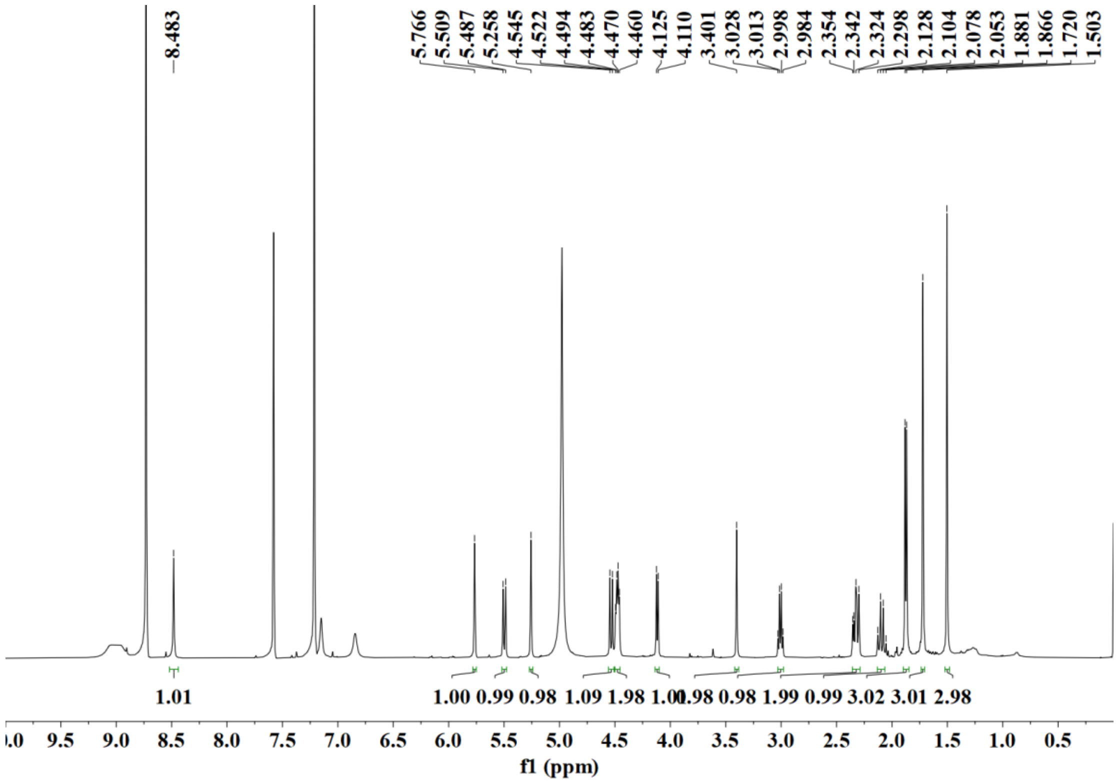

Figure S104. ^{1}H NMR spectrum of compound **10**

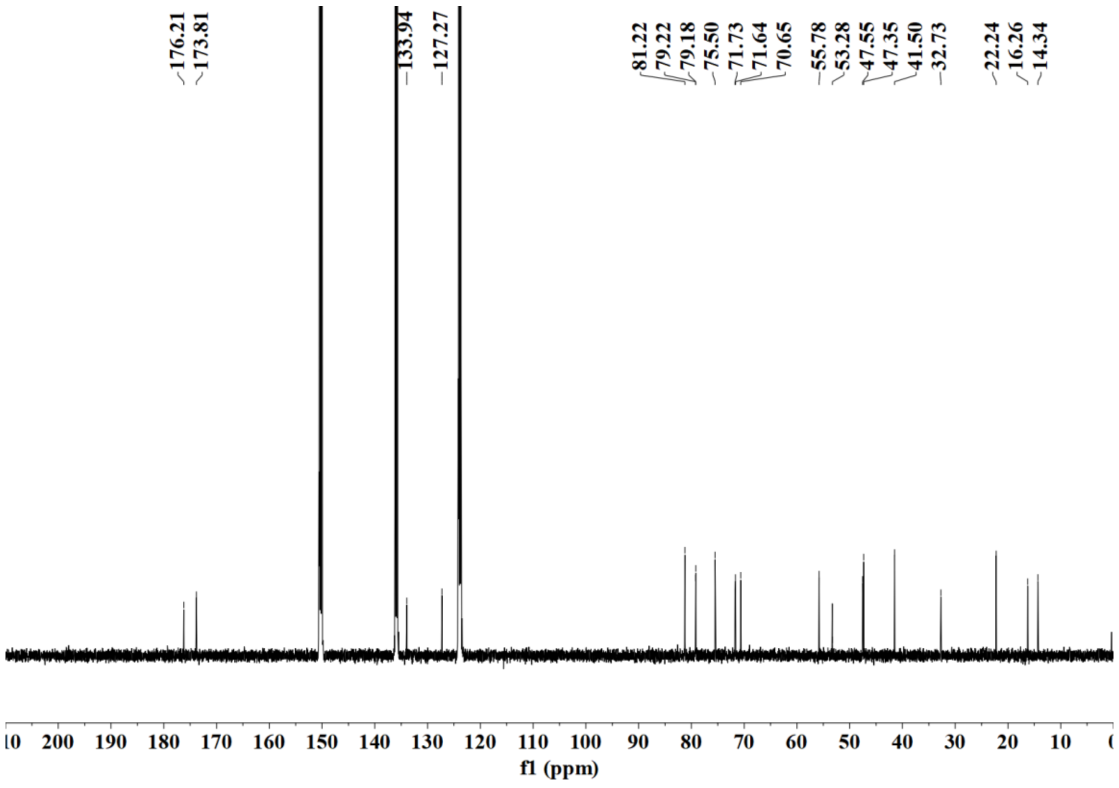

Figure S105. ^{13}C NMR spectrum of compound **10**

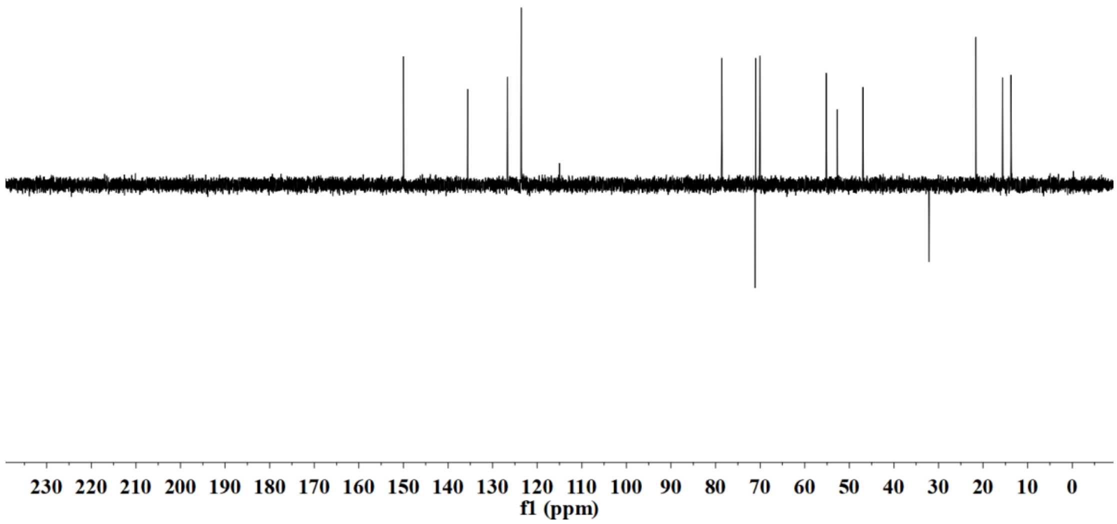

Figure S106. DEPT-135 spectrum of compound **10**

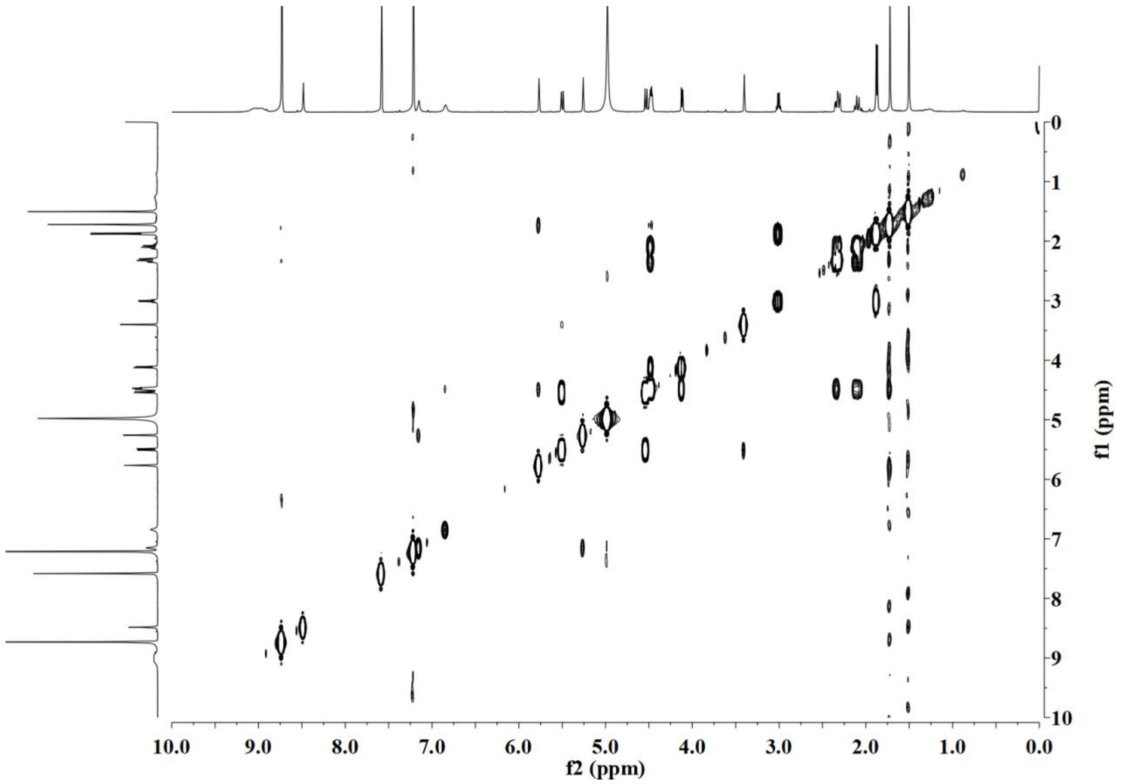

Figure S107. ^{1}H-^{1}H COSY spectrum of compound **10**

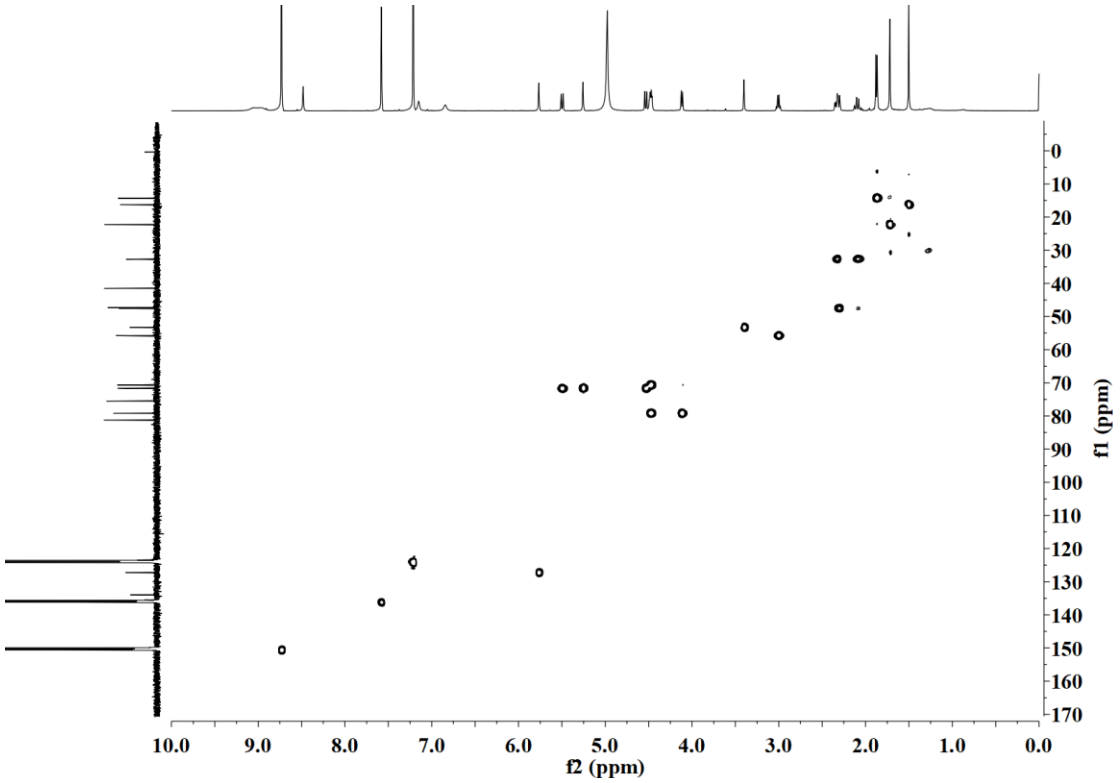

Figure S108. HSQC spectrum of compound **10**

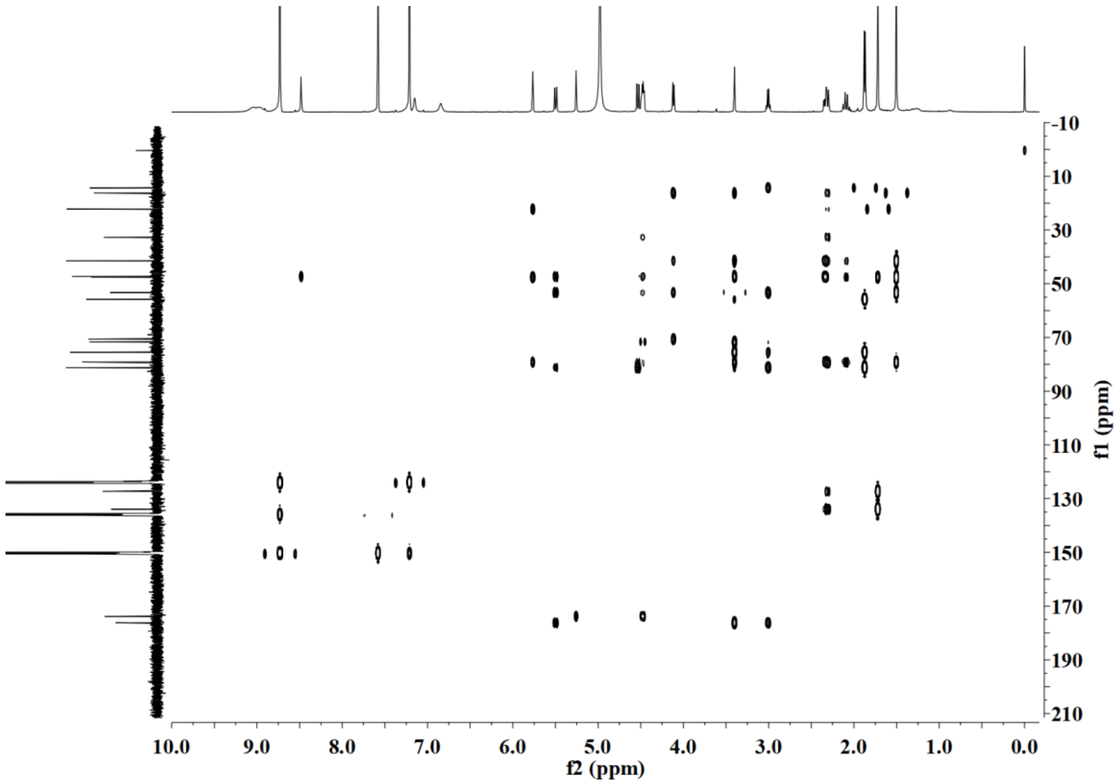

Figure S109. HMBC spectrum of compound **10**

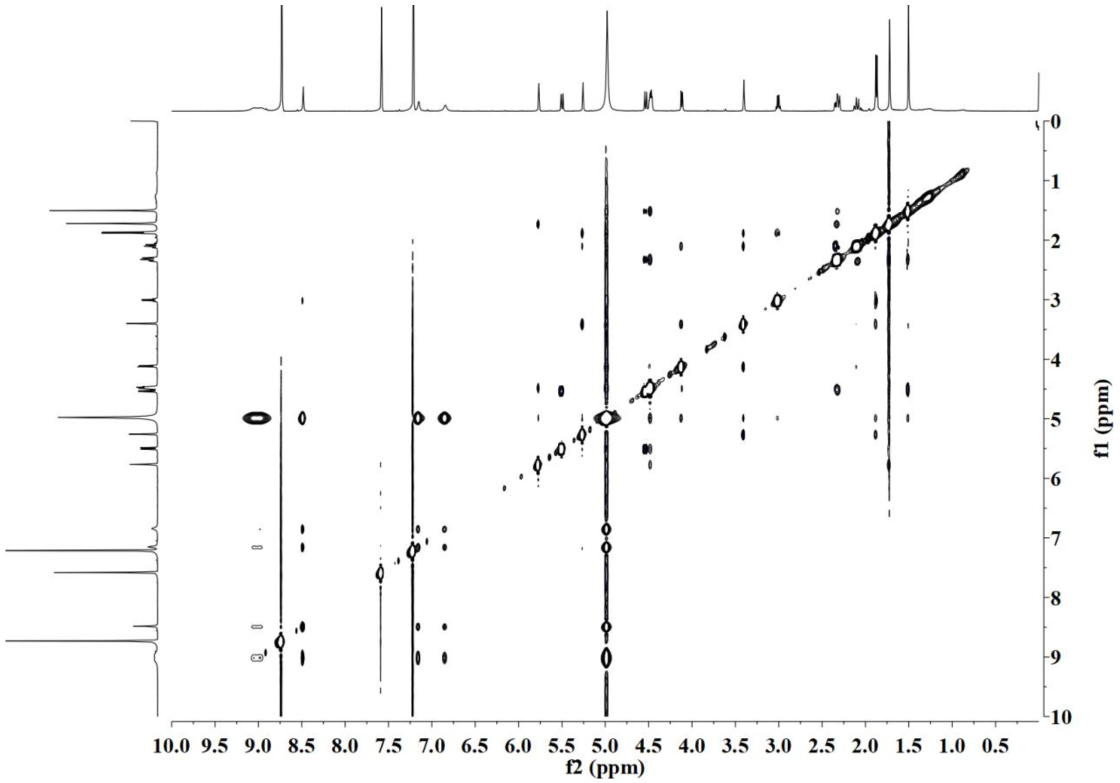

Figure S110. NOESY spectrum of compound **10**

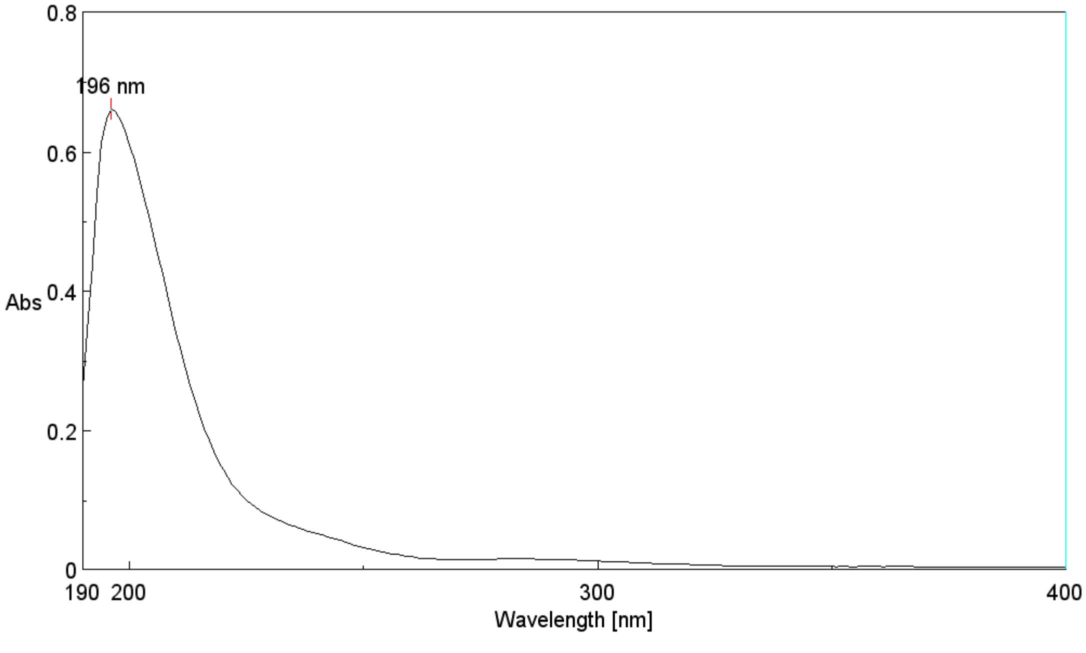

Figure S111. UV spectrum of compound **11**

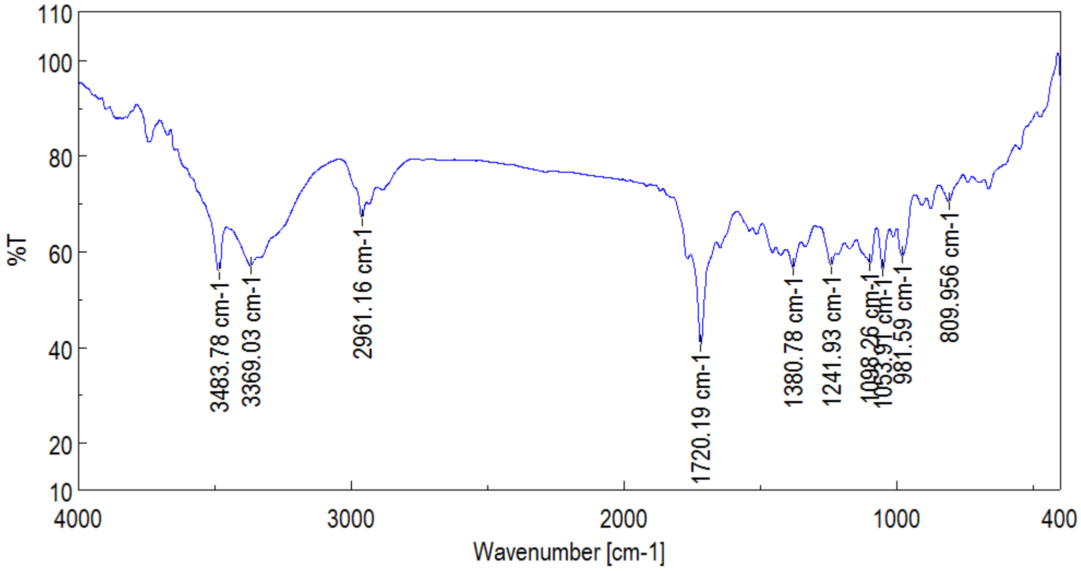

Figure S112. IR (KBr disc) spectrum of compound **11**

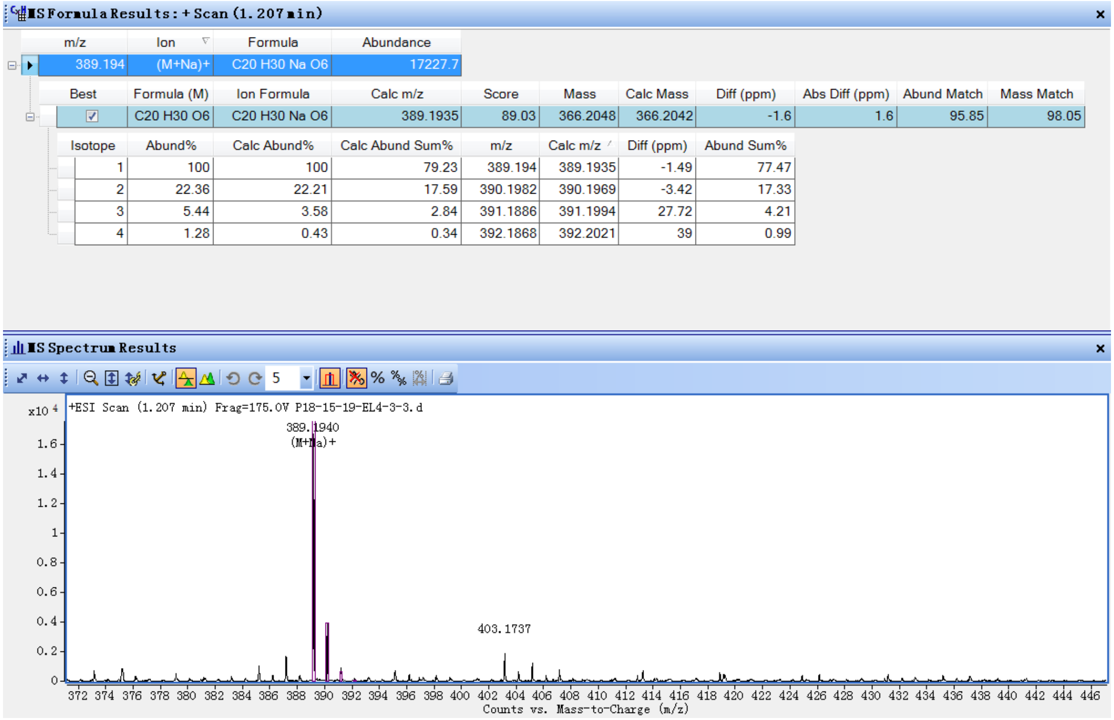

Figure S113. HR-ESI-MS spectrum of compound **11**

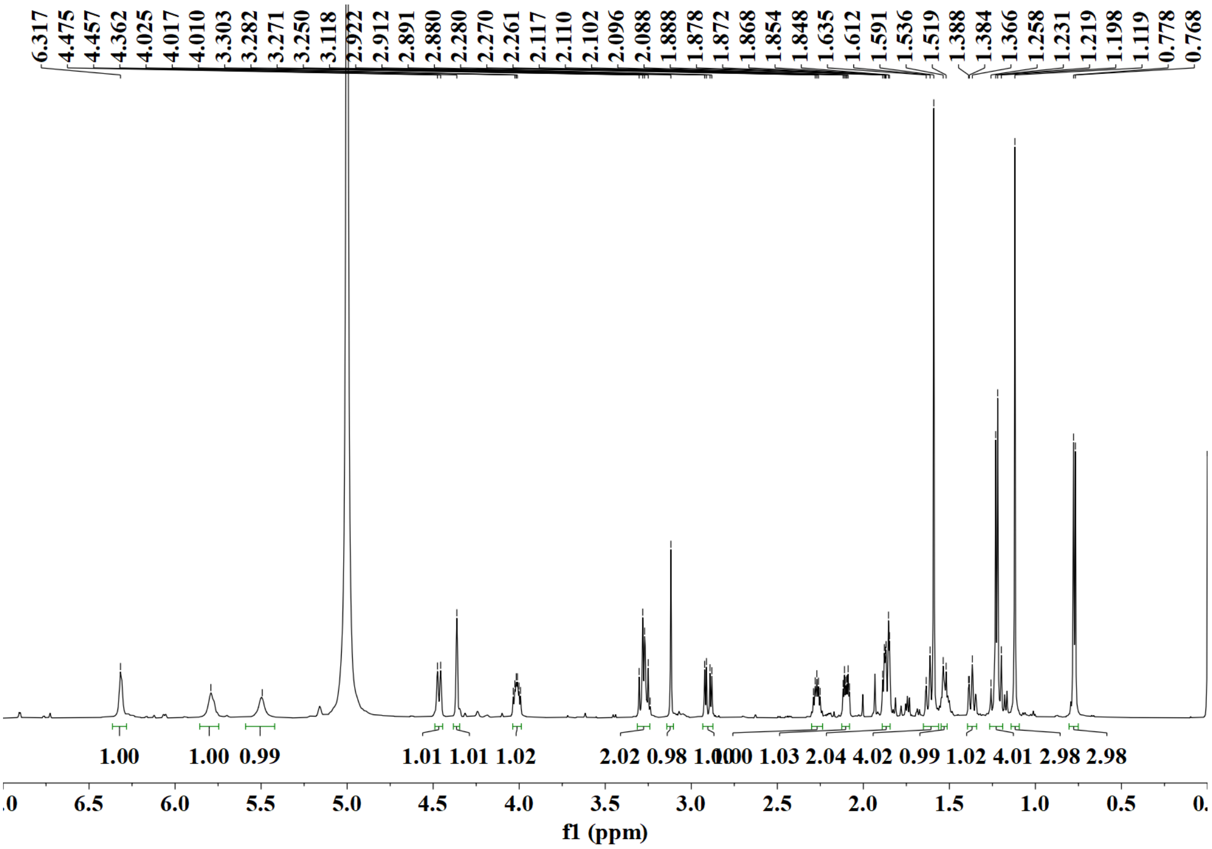

Figure S114. ^{1}H NMR spectrum of compound **11**

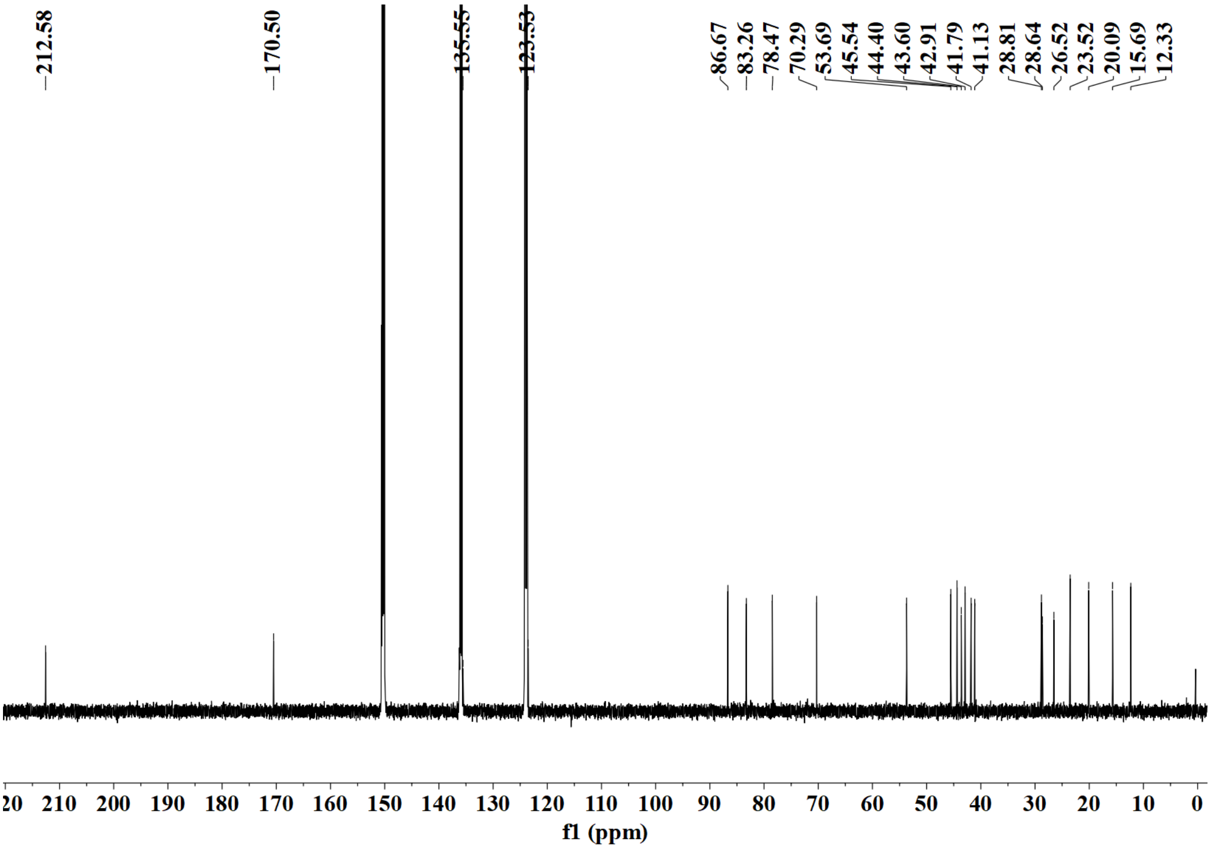

Figure S115. ^{13}C NMR spectrum of compound **11**

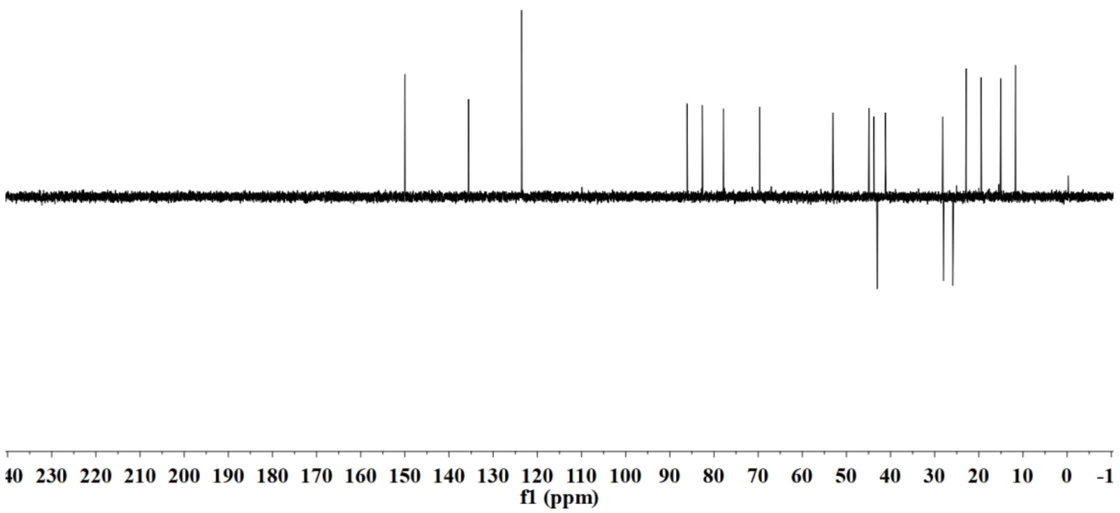

Figure S116. DEPT-135 spectrum of compound **11**

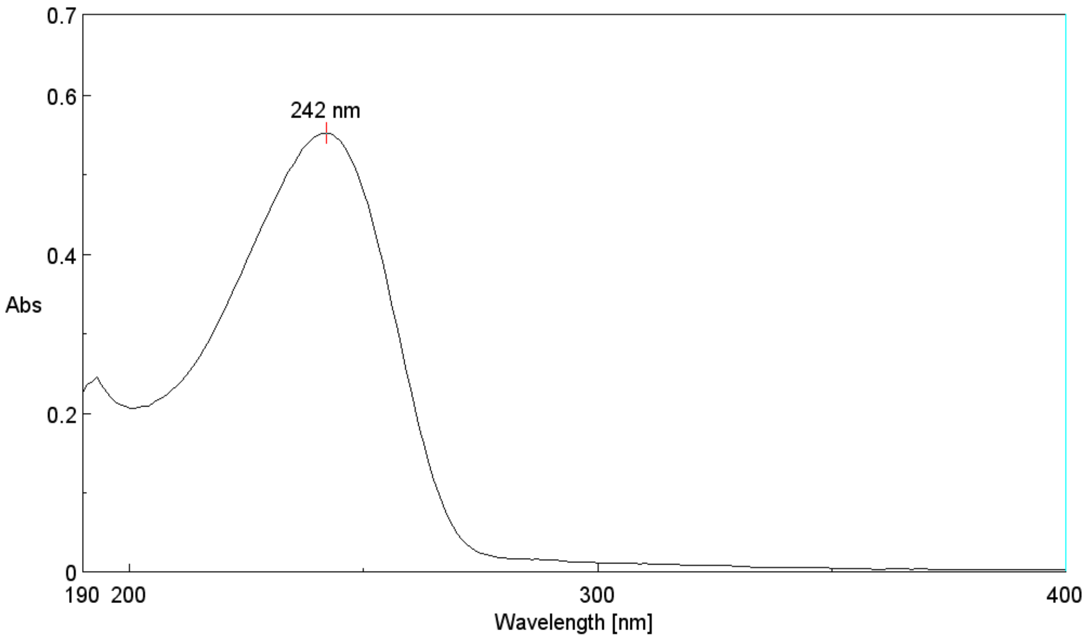

Figure S117. UV spectrum of compound **12**

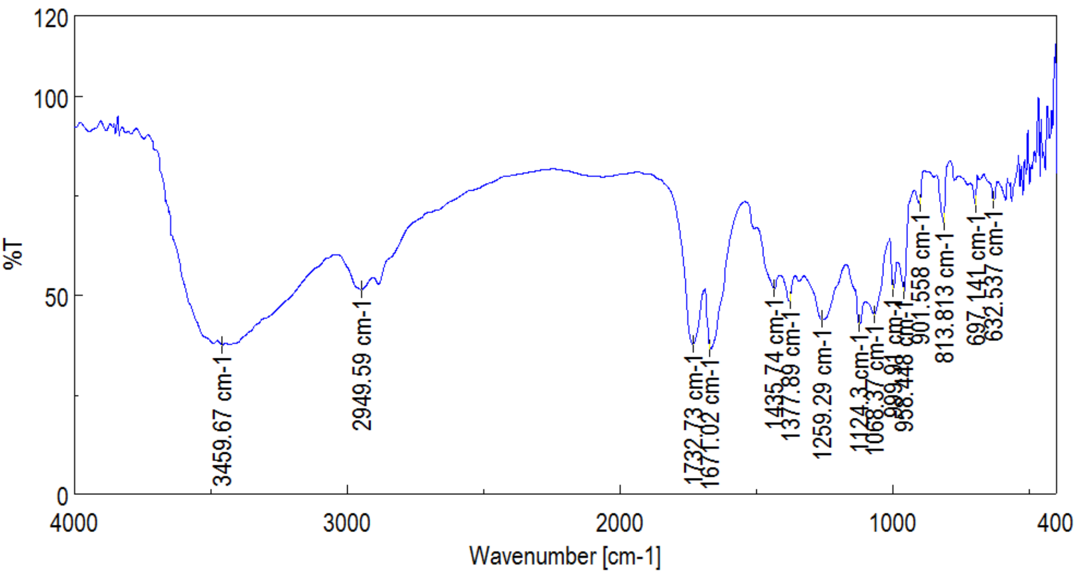

Figure S118. IR (KBr disc) spectrum of compound **12**

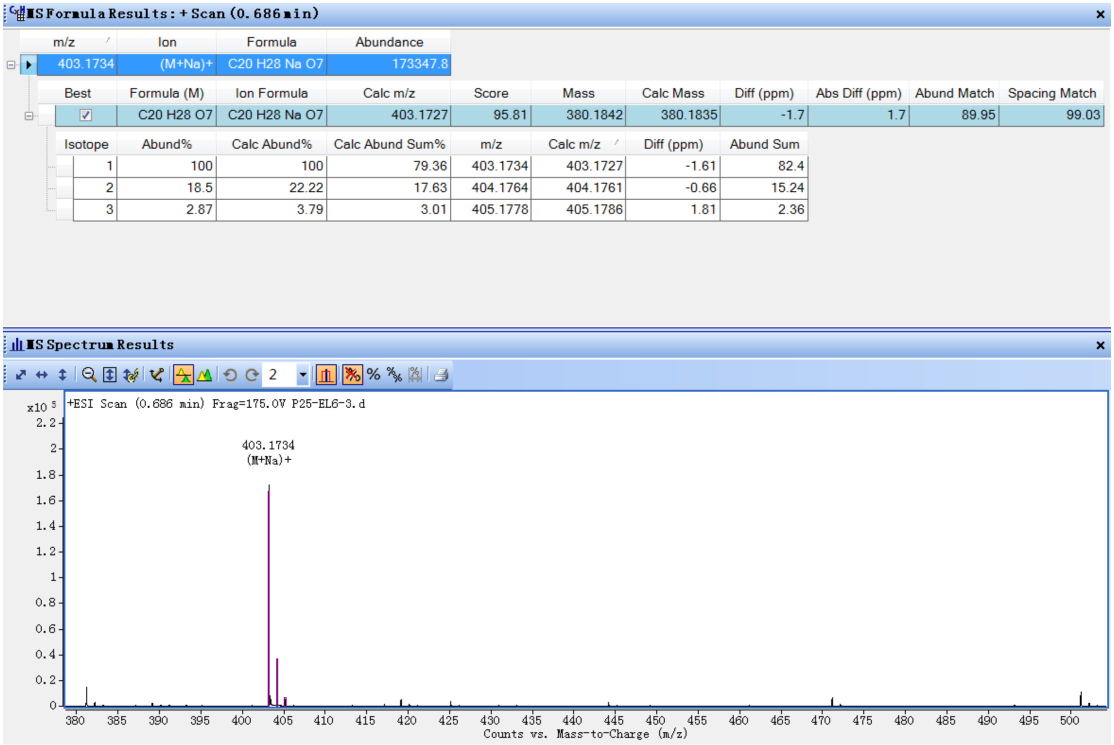

MS Formula Results: + Scan (0.686 min)

m/z	Ion	Formula	Abundance
403.1734	(M+Na)+	C20 H28 Na O7	173347.8

Best	Formula (M)	Ion Formula	Calc m/z	Score	Mass	Calc Mass	Diff (ppm)	Abs Diff (ppm)	Abund Match	Spacing Match
☑	C20 H28 O7	C20 H28 Na O7	403.1727	95.81	380.1842	380.1835	-1.7	1.7	89.95	99.03

Isotope	Abund%	Calc Abund%	Calc Abund Sum%	m/z	Calc m/z	Diff (ppm)	Abund Sum
1	100	100	79.36	403.1734	403.1727	-1.61	82.4
2	18.5	22.22	17.63	404.1764	404.1761	-0.66	15.24
3	2.87	3.79	3.01	405.1778	405.1786	1.81	2.36

Figure S119. HR-ESI-MS spectrum of compound **12**

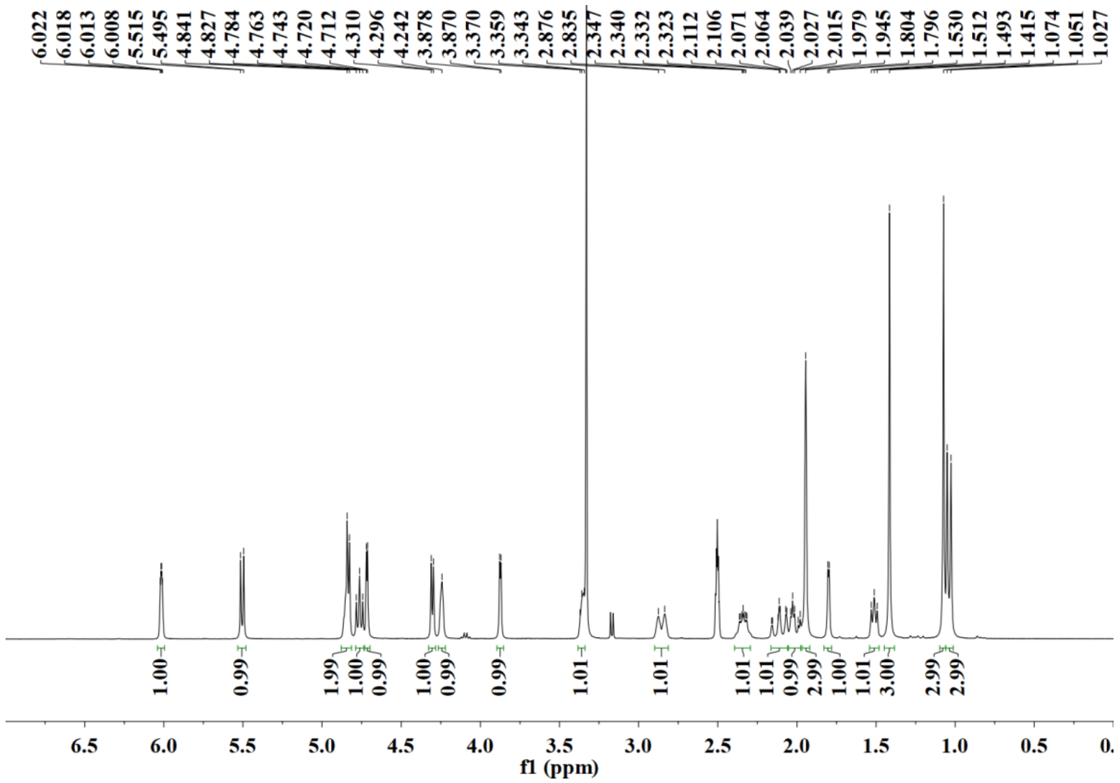

Figure S120. ^{1}H NMR spectrum of compound **12**

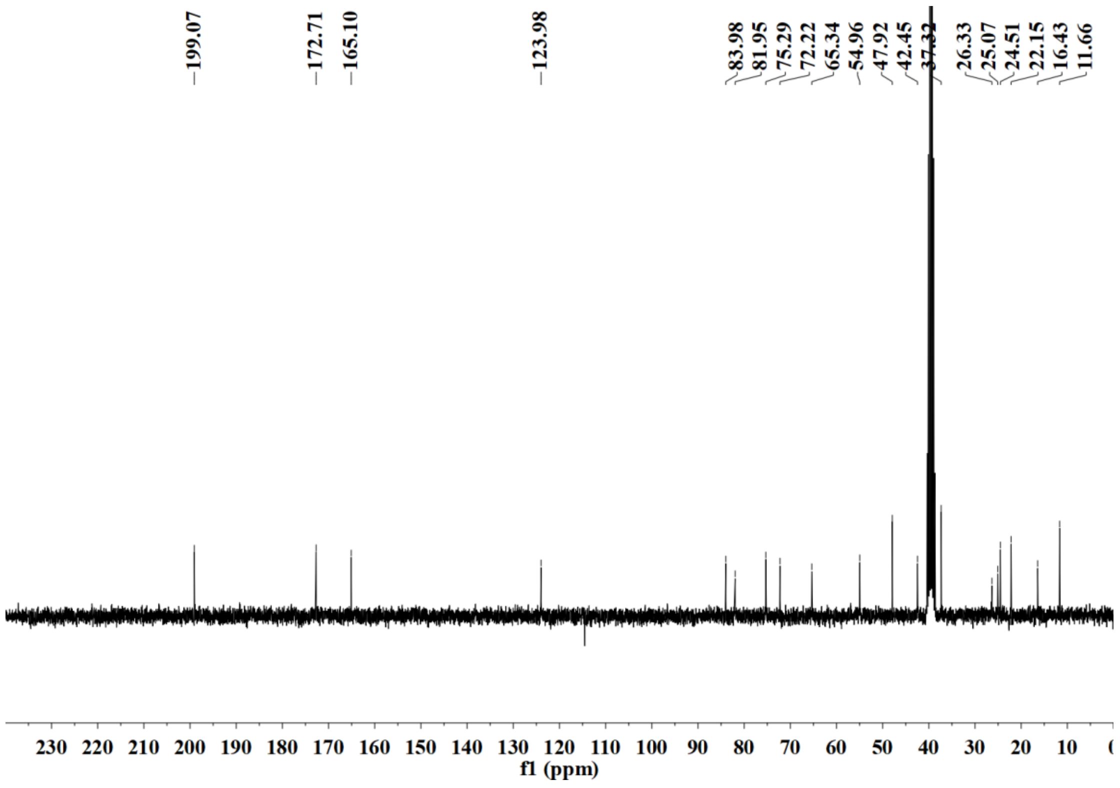

Figure S121. ^{13}C NMR spectrum of compound **12**

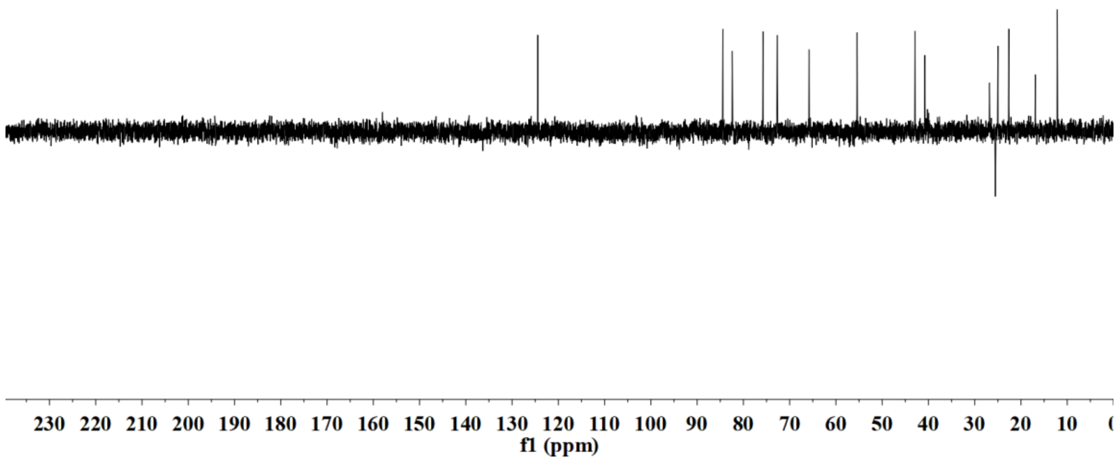

Figure S122. DEPT-135 spectrum of compound **12**

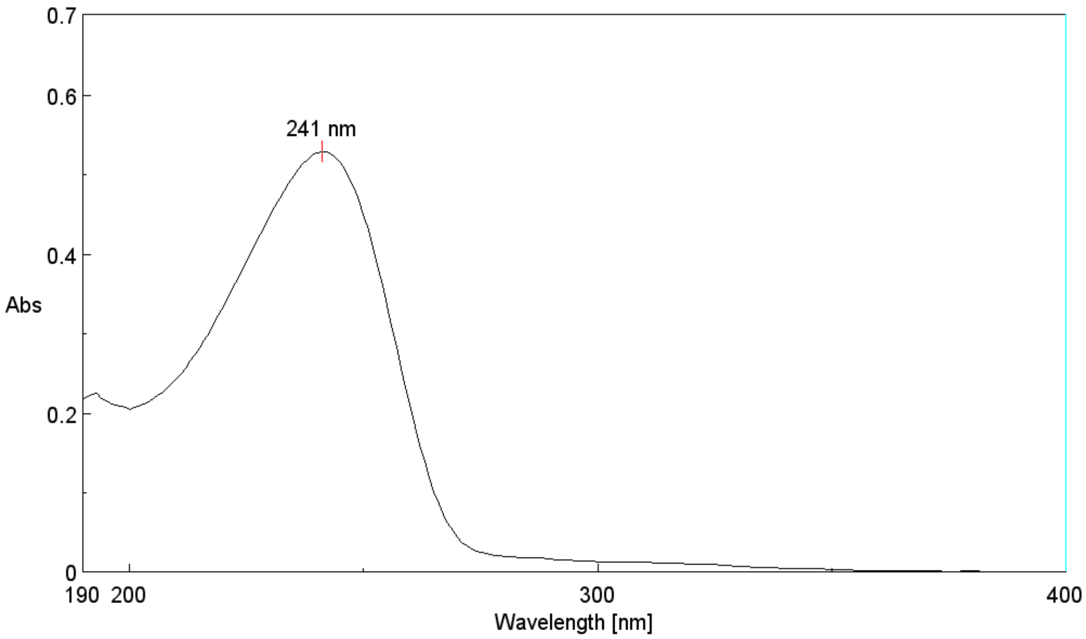

Figure S123. UV spectrum of compound **13**

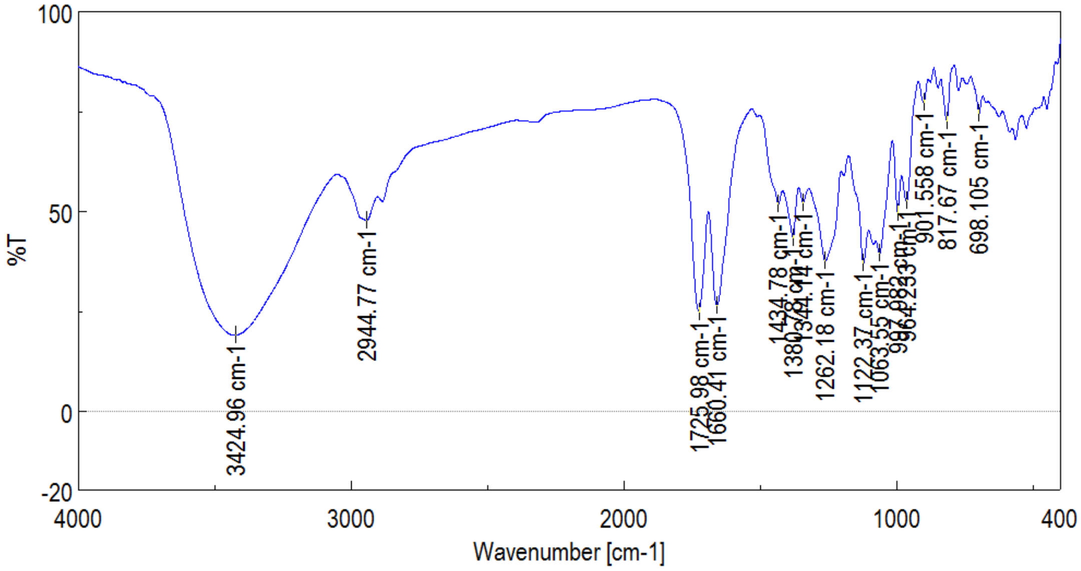

Figure S124. IR (KBr disc) spectrum of compound **13**

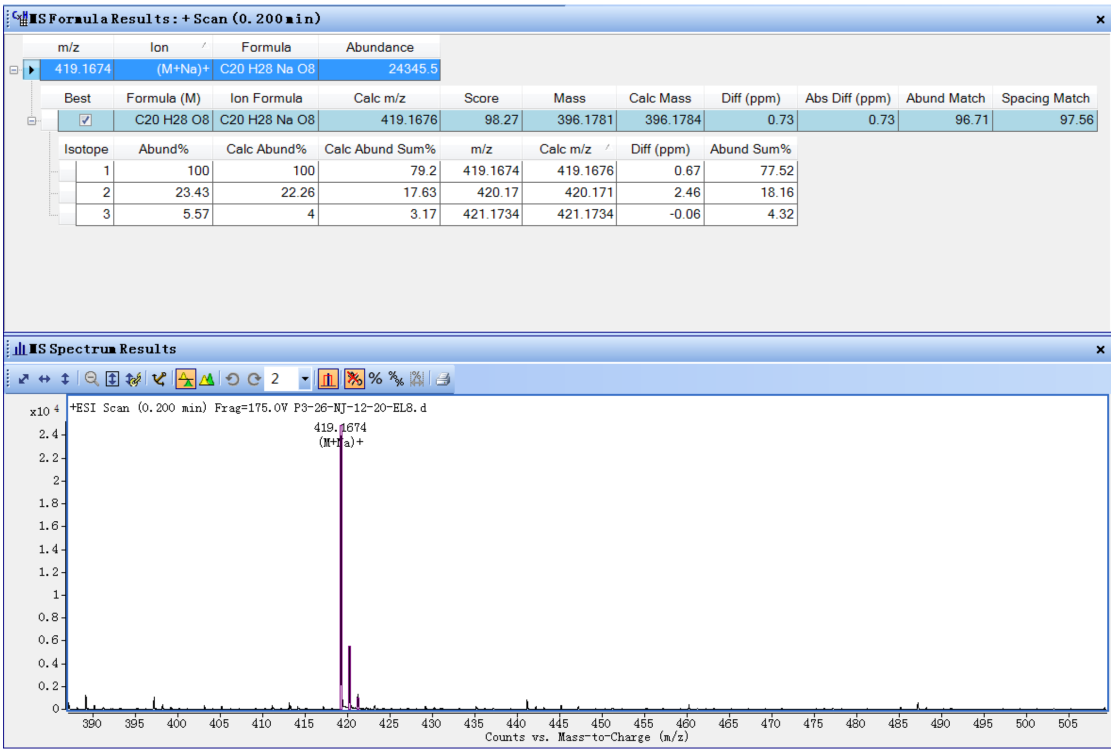

Figure S125. HR-ESI-MS spectrum of compound **13**

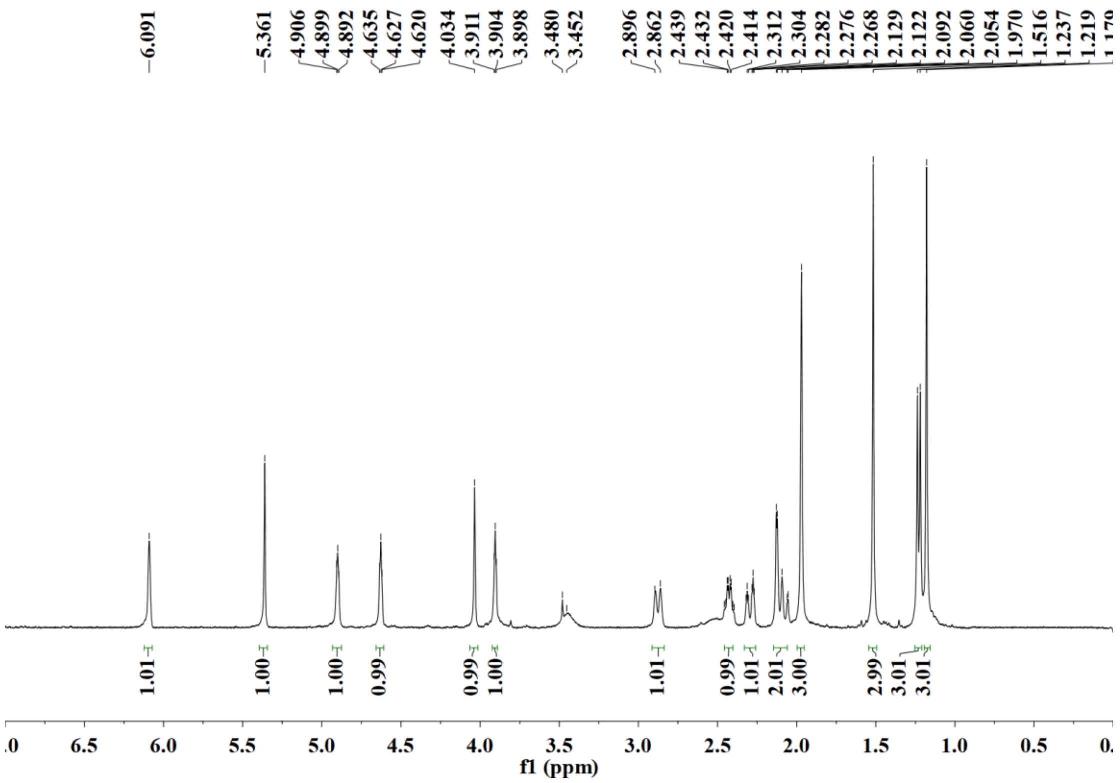

Figure S126. ^{1}H NMR spectrum of compound **13**

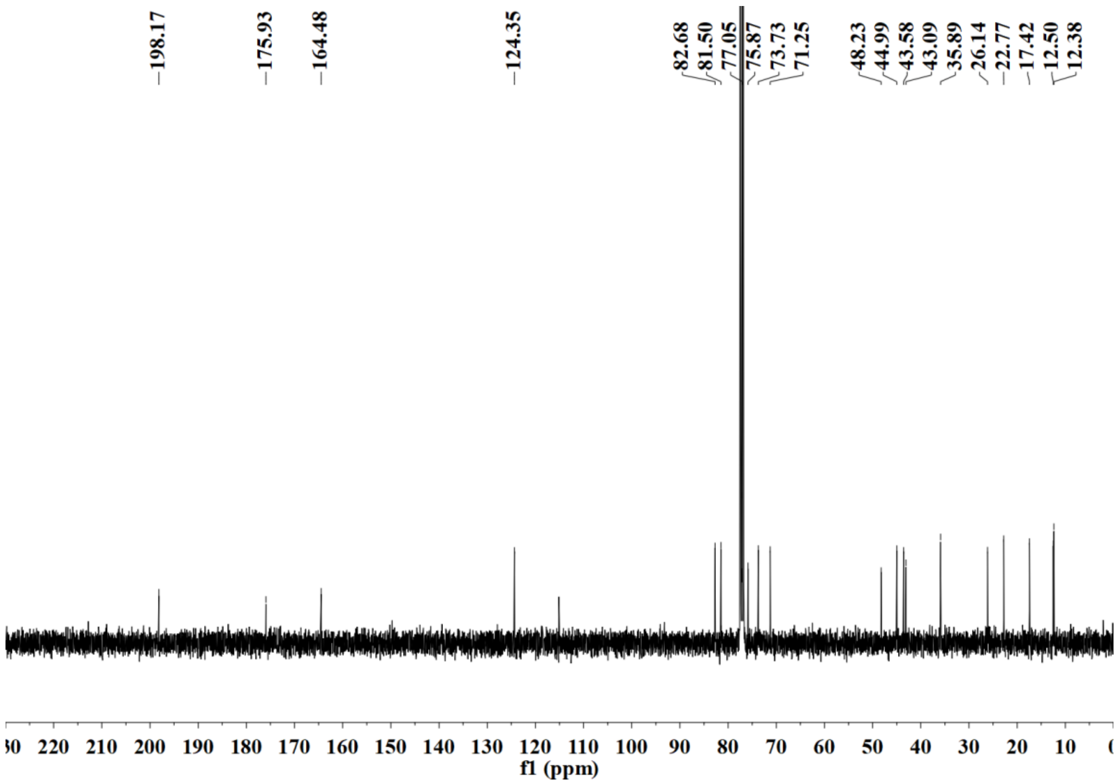

Figure S127. ^{13}C NMR spectrum of compound **13**

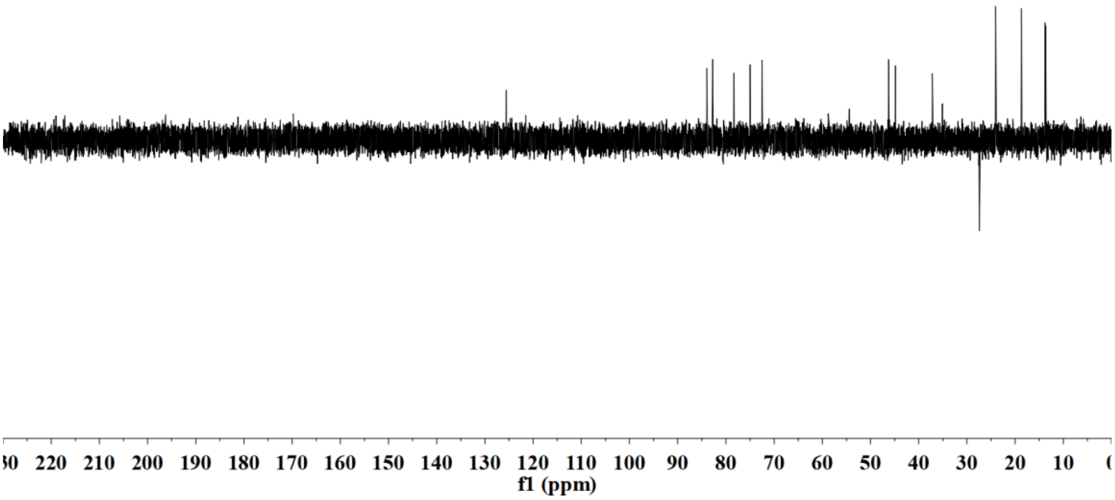

Figure S128. DEPT-135 spectrum of compound **13**

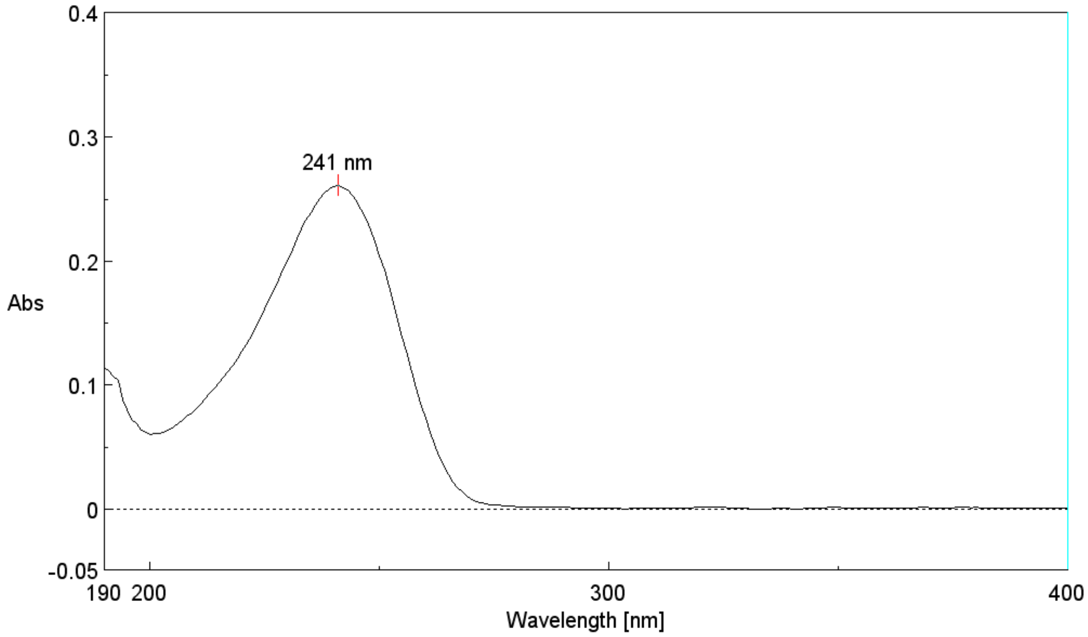

Figure S129. UV spectrum of compound **14**

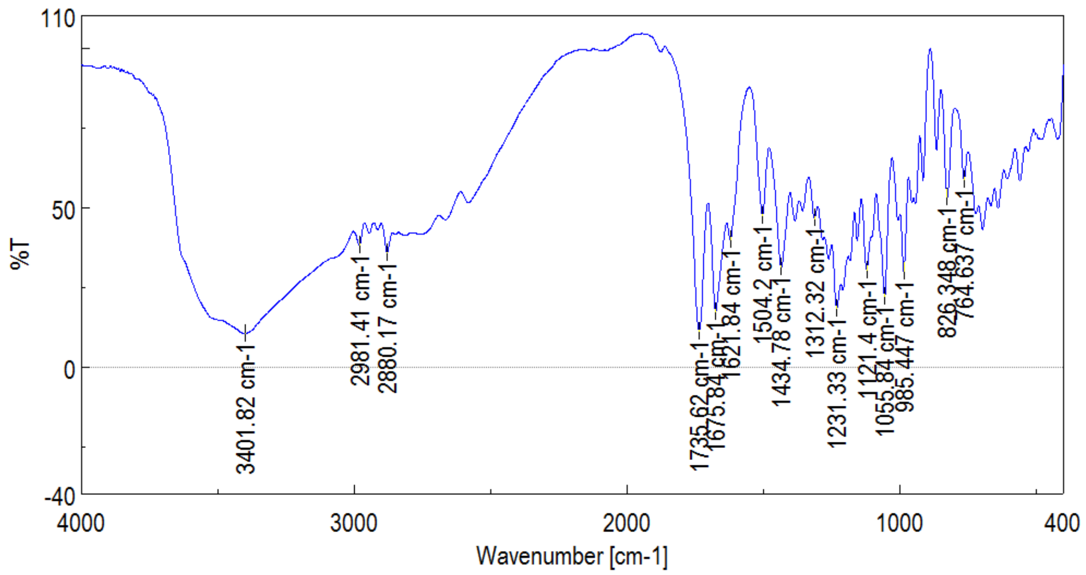

Figure S130. IR (KBr disc) spectrum of compound **14**

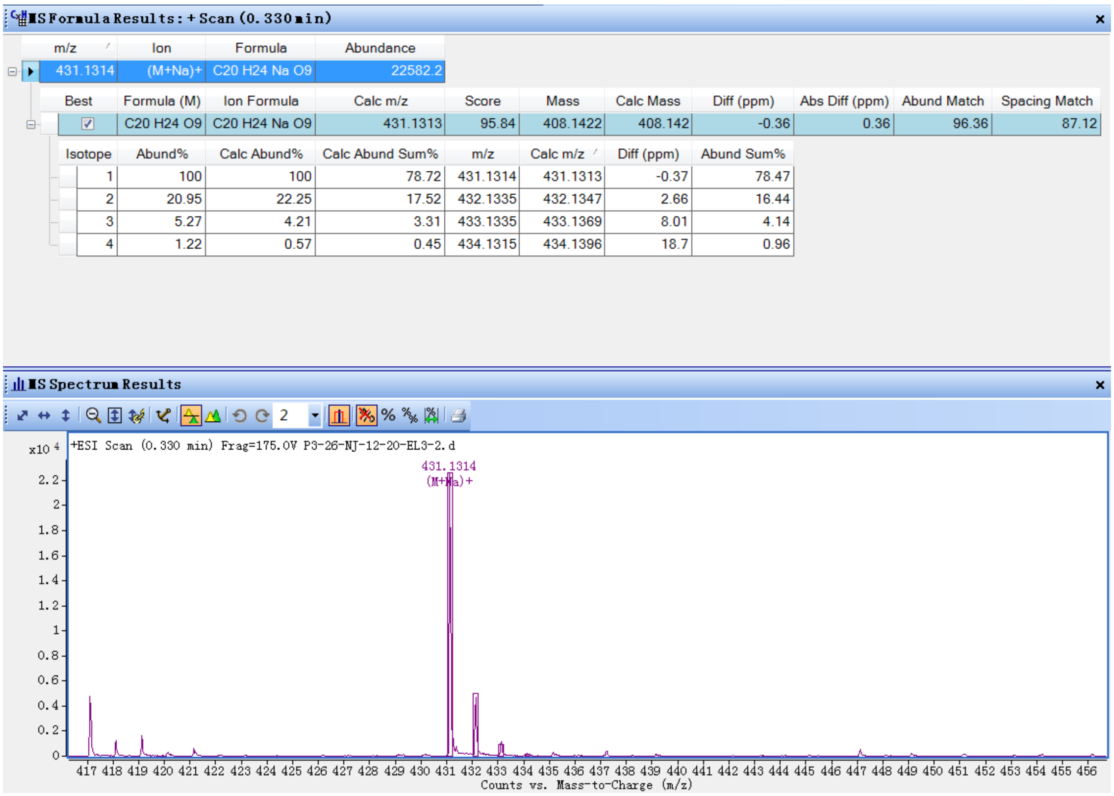

MS Formula Results: + Scan (0.330 min)

m/z	Ion	Formula	Abundance
431.1314	(M+Na)+	C20 H24 Na O9	22582.2

Best	Formula (M)	Ion Formula	Calc m/z	Score	Mass	Calc Mass	Diff (ppm)	Abs Diff (ppm)	Abund Match	Spacing Match
☑	C20 H24 O9	C20 H24 Na O9	431.1313	95.84	408.1422	408.142	-0.36	0.36	96.36	87.12

Isotope	Abund%	Calc Abund%	Calc Abund Sum%	m/z	Calc m/z	Diff (ppm)	Abund Sum%
1	100	100	78.72	431.1314	431.1313	-0.37	78.47
2	20.95	22.25	17.52	432.1335	432.1347	2.66	16.44
3	5.27	4.21	3.31	433.1335	433.1369	8.01	4.14
4	1.22	0.57	0.45	434.1315	434.1396	18.7	0.96

Figure S131. HR-ESI-MS spectrum of compound **14**

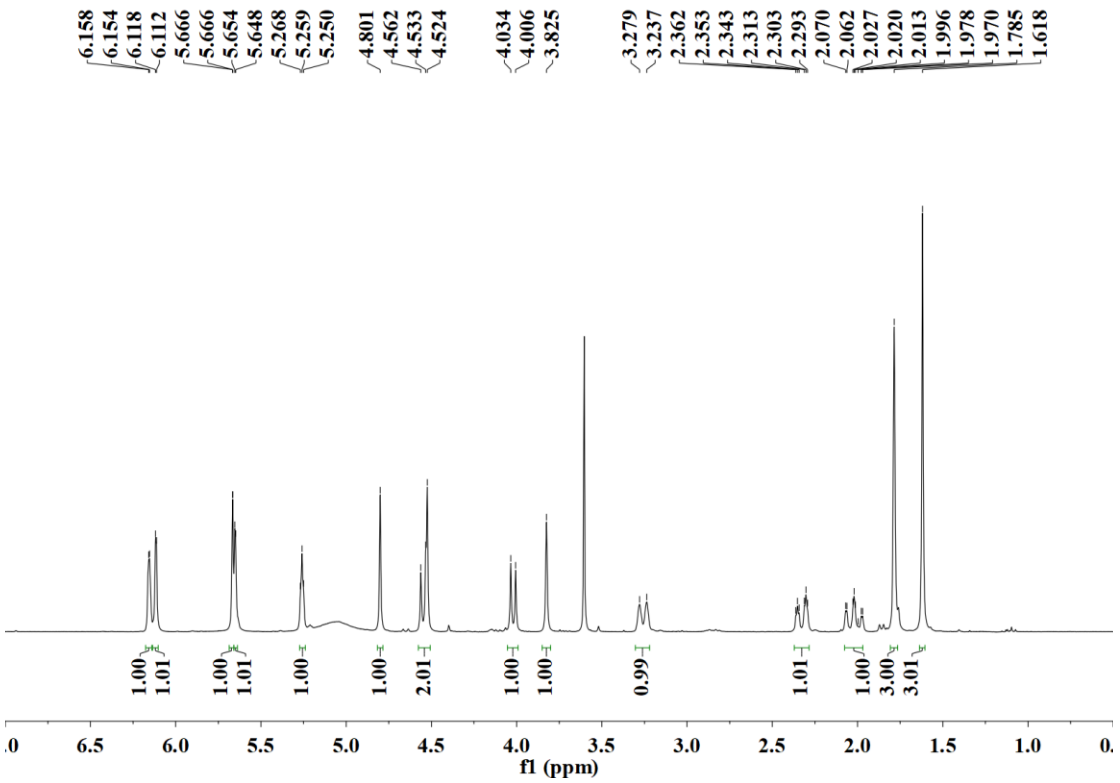

Figure S132. ^{1}H NMR spectrum of compound **14**

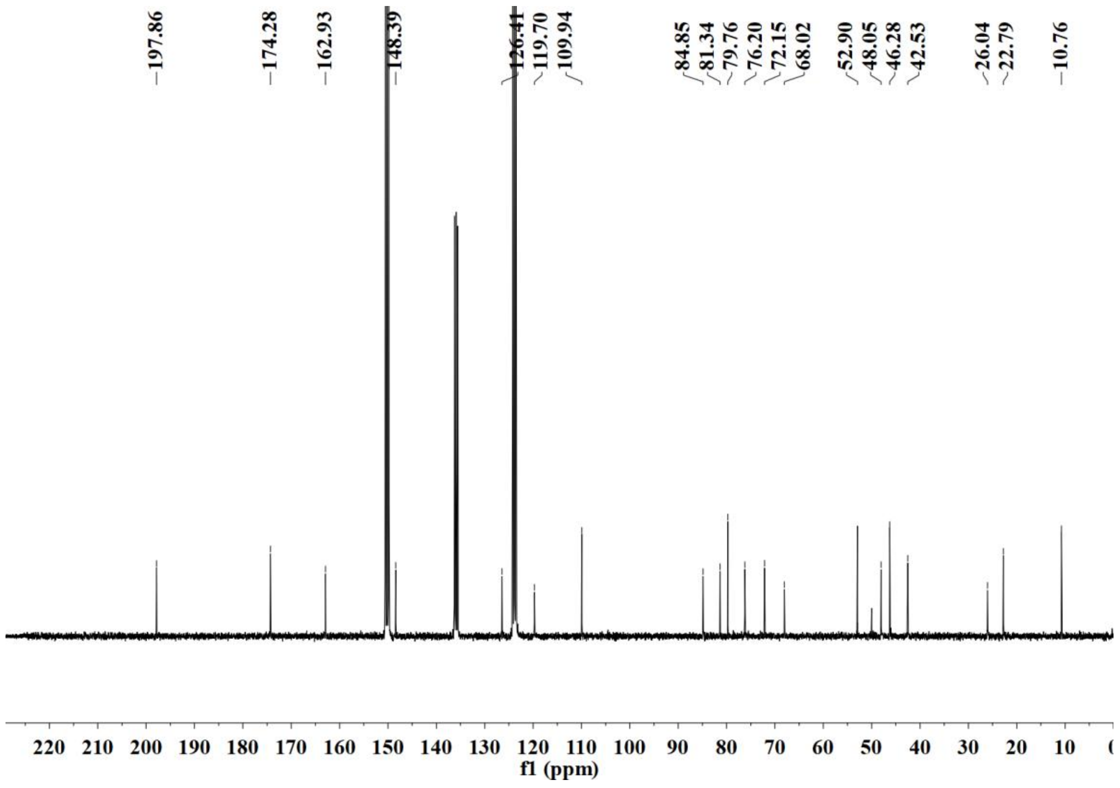

Figure S133. ^{13}C NMR spectrum of compound **14**

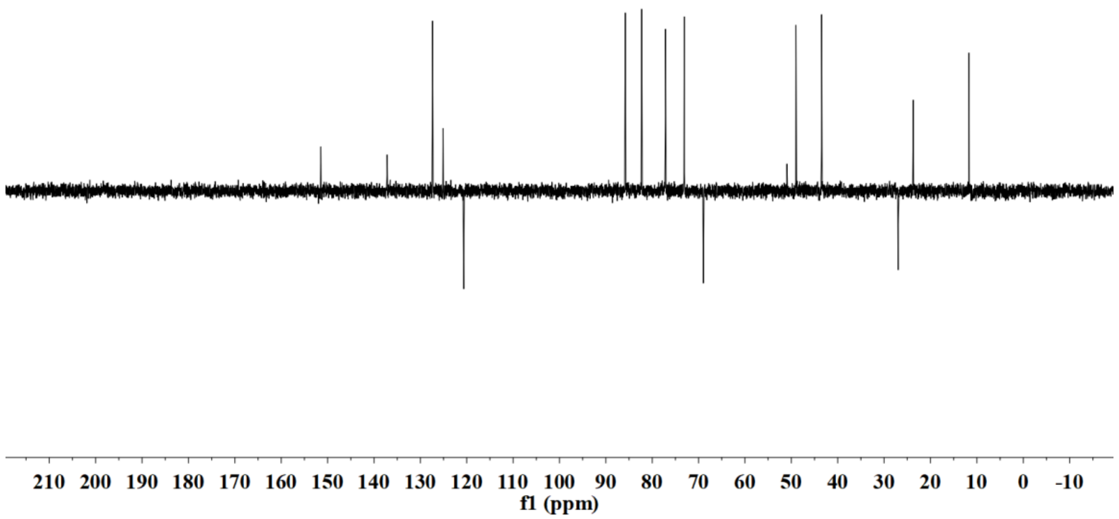

Figure S134. DEPT-135 spectrum of compound **14**
